# Evolution of seed characters and of dispersal modes in Aizoaceae

**DOI:** 10.3389/fpls.2023.1140069

**Published:** 2023-03-22

**Authors:** Alexander P. Sukhorukov, Maya V. Nilova, Maria Kushunina, Yuri Mazei, Cornelia Klak

**Affiliations:** ^1^ Department of Higher Plants, Biological Faculty, M.V. Lomonosov Moscow State University, Moscow, Russia; ^2^ Laboratory Herbarium (TK), Tomsk State University, Tomsk, Russia; ^3^ Department of Plant Physiology, Biological Faculty, M.V. Lomonosov Moscow State University, Moscow, Russia; ^4^ Department of General Ecology and Hydrobiology, Biological Faculty, M.V. Lomonosov Moscow State University, Moscow, Russia; ^5^ Bolus Herbarium, Department of Biological Sciences, University of Cape Town, Cape Town, South Africa

**Keywords:** Caryophyllales, anatomy, molecular phylogeny, pyrophytic habitat, reproductive biology

## Abstract

The family Aizoaceae includes ~1880 species and is one of the more diverse groups within Caryophyllales, particularly in arid areas in the western part of southern Africa. Most species are dwarf succulent-leaf shrubs. In response to the harsh climatic conditions prevalent where they occur, many representatives have evolved special reproductive adaptations. These include hygrochastic capsules (mostly found in Mesembryanthemoideae and Ruschioideae), burr-like indehiscent and one-seeded, winged diaspores, and fast germination of seeds after rain. We focused on anatomical features, evolutionary trends, and the ecological significance of various morpho-anatomical structures found in the seeds. The seeds of 132 species from 61 genera were studied, and 18 diagnostic characters were discovered. All studied characters were compared with those of other families from core Caryophyllales. The seed notch and embryo shape were added to the list of characteristics distinguishing major clades within the family. In addition, the presence of longitudinal ridges and a keel on the seed are additional characters of Aizooideae and combined Ruschioideae-Apatesieae, respectively. Puzzle-like borders of testa cells are a common trait in Ruschioideae and Mesembryanthemoideae. Most taxa in Aizoaceae have a thin seed coat, which is the ancestral state within the family. This may facilitate fast germination. We observed several shifts to a medium-thick or thick seed coat in members of Ruschioideae and Acrosanthoideae. These inhabit fire-prone environments (in vegetation types known as *fynbos* and *renosterveld*), where the thickened seed coat may protect against damage by fire. Multi-seeded fruits are the ancestral state within Aizoaceae, with several shifts to one-(two-)seeded xerochastic fruits. The latter are dispersed *via* autochory, zoochory, or anemochory. This trait has evolved mainly in less succulent subfamilies Acrosanthoideae, Aizooideae, and Sesuvioideae. In highly succulent subfamilies Ruschioideae and Mesembryanthemoideae, fruits are almost exclusively multi-seeded and hygrochastic with ombrohydrochoric dispersal. A reduction in the number of seeds within a dispersal unit is rare. Within Apatesieae and Ruschieae, there are also a few unusual genera whose fruits fall apart into one- to two-seeded mericarps (that are mainly dispersed by wind).

## 1 Introduction

Aizoaceae is one of the most diverse families in core Caryophyllales comprising ~1880 species in 122 genera (Hernández-Ledesma et al., 2015; Hartmann, 2017). The region with the highest diversity of Aizoaceae is southern Africa, especially the Greater Cape floristic region (Born et al., 2007; Manning and Goldblatt, 2012; Snijman, 2013). Some representatives are widespread in other tropical regions of the Old and New World, especially in Australia and Americas (Chinnock, 1983; Bohley et al., 2017; Klak et al., 2017b).

**Figure 1:**
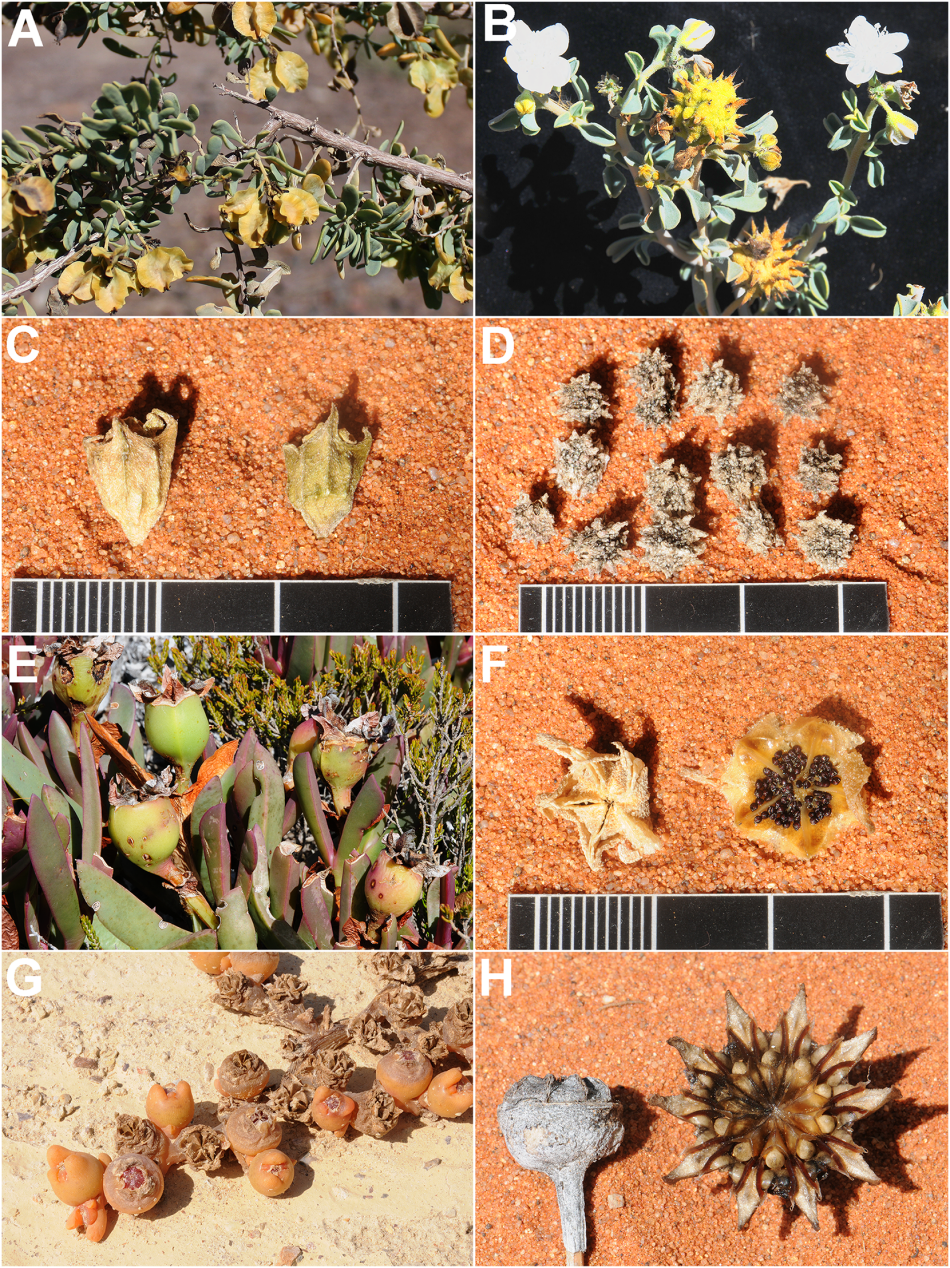
Xerochastic **(A-E)** and hygrochastic **(F-H)** fruits of selected Aizoaceae species. **(A)** *Anisostigma schenckii*, **(B)** *Tribulocarpus dimorphanthus*, **(C)** *Tetragonia tetragonoides*, **(D)** *Tetragonia echinata*, **(E)** *Carpobrotus acinaciformis*, **(F)** Top view of closed (l) and open (r) fruit, *Aizoanthemopsis hispanica*, **(G)** *Mesembryanthemum cryptanthum*, and **(H)** Side view of closed (l) and open (r) fruit, *Cheiridopsis alba-oculata*.

Five subfamilies are recognized: Acrosanthoideae Klak, Aizooideae (incl. Tetragonioideae Lindl.), Mesembryanthemoideae Ihlenf., Schwantes & Straka, Ruschioideae Schwantes, and Sesuvioideae Lindl. (Klak et al., 2017b; **Table 1**). The subfamilies and tribes are very different in size. Ruschieae Schwantes (Ruschioideae) is the most species-rich, while Acrosanthoideae is the smallest and most range-restricted and occurs only in the South-Western Cape of South Africa. Major clades within Aizoaceae can be circumscribed by several distinctive characteristics including the life form, pubescence, reproductive traits (especially the color of perianth segments), the shape of nectaries, morphology of fruits, position of the gynoecium, and the number of ovules (Hartmann, 1988; Chesselet et al., 2002; Klak et al., 2003; Hartmann, 2017). Additionally, many Aizoaceae members have evolved hygrochasy, where fruits open rapidly when moistened (e.g., Schmid, 1925; Ihlenfeldt and Gerbaulet, 1990; Hartmann, 1991; Kurzweil and Burgoyne, 2009; **Figure 1**).

**Table 1:** Number of genera and species in subfamilies and tribes of Aizoaceae, their geographic ranges (Klak and Bruyns, 2012; Hernández-Ledesma et al., 2015; Bohley et al., 2017; Klak et al., 2017a; Klak et al., 2020), and distinguishing seed characteristics.

Subfamily	Tribe	No. of genera	No. of species	Geographic range	Distinguishing states of seed characteristics
**Acrosanthoideae**	–	1	6	South-Western Cape (South Africa)	Seeds black (4:3), reniform (5:2), large (6:3), ridged (9:1); seed surface foveolate (12:1); embryo shape annular (16:0), with thick coat (17:2)
**Aizooideae**	–	7	124	Mediterranean and subtropical areas in Africa, Eurasia, Australasia, and South America	Three very different groups present: (1) Seeds black (4:3), reniform (5:2), ridged (9:1), usually foveolate (12:1) seeds without puzzle-like cell borders (11:0); waxes absent (13:0); testa thin to thick, with stalactites (18:1), [*Aizoanthemum*, *Aizoanthemopsis*, *Aizoon*]. (2) Seeds black (4:3) or brown (4:1), of diverse shape (5:2, 5:3, 5:4), ridged at seed margin (9:2) or not (9:0), puzzle-like cell borders present (11:1); not foveolate (12:0), waxes present (13:1); testa thin or moderately thick (17:0, 17:1), without or with stalactites (18:0, 18:1) [*Gunniopsis*]. (3) Seeds yellow-brown (4:0), pear-shaped (5:4), not ridged (9:0), puzzle-like cell borders absent (11:0), not foveolate (12:0), waxes absent (13:0), testa thin (17:0), without stalactites (18:0) [*Tetragonia*]
**Mesembryanthemoideae**	–	1	105	mainly southern Africa	Seeds usually brown (4:1), D-shaped (5:3), not keeled (7:0), notched (8:1), not ridged (9:0), with puzzle-like cell borders (11:1), usually not foveolate (12:0), waxes present (13:1), embryo bent (16:2), testa thin (17:0), stalactites absent or cell walls with white fortifications (18:0). *Mesembryanthemum nucifer* deviates from other species in many characteristic states
**Ruschioideae**	Apatesieae	5	11	Western and Northern Cape (South Africa)	Ruschioideae (in general): Seeds not ridged (9:0), usually with puzzle-like cell borders (11:1), not foveolate (12:0), with waxes (13:1) except some Apatesieae, dark stalactites usually absent (18:0) Seeds brown (4:1) or black (4:3); depressed-roundish (5:1) or reniform (5:2); medium-sized or large (6:2, 6:3); keeled (7:1) in *Carpanthea*, *Conicosia* and *Hymenogyne glabra*; seed notch absent (8:0) except *Skiatophytum*, ultrasculpture mamillate (10:3) except *Conicosia* with smooth surface (10:0), waxes absent (13:0) in *Hymenogyne* and *Skiatophytum* or present (13:1) in remainder of the genera, embryo annular (16:0) but r-shaped (16:1) in *Skiatophytum*
	Dorotheantheae	1	14	Winter rainfall area of South Africa	Seeds brown (4:1) or black (4:3); usually D-shaped (5:3), seed notch present (8:1), ultrasculpture papillate (10:2) or mamillate (10:3), puzzle-like cell borders usually absent (11:0), embryo r-shaped (16:1) or bent (16:2)
	Drosanthemeae	2	108	Namibia and South Africa	Seeds different in color and shape, seed notch usually present (8:1), ultrasculpture rugose (10:1), papillate (10:2) or mamillate (10:3), puzzle-like cell borders present (11:1), embryo straight (16:1), rarely annular (16:0)
	Ruschieae	103	~1585	mainly southern Africa	Seeds different in color and shape but mostly yellow-brown (4:0) or brown (4:1), D-shaped (5:3) or pear-shaped (5:4), seed notch present (8:1) especially in D-shaped seeds, but usually absent in ovate or pear-shaped seeds, ultrasculpture different but in most cases papillate (10:2) or mamillate (10:3), sometimes finger-like (10:5), puzzle-like cell borders present (11:1), embryo straight, r-shaped (16:1) or bent (16:2)
**Sesuvioideae**	Anisostigmateae	2	4	Namibia and East Africa	Sesuvioideae (in general): Seed notch absent (8:0), puzzle-like borders absent (11:0), waxes absent (13:0) in non-arillate seeds or at least in seeds with easily ruptured aril, embryo annular (16:0), rarely bent (16:2) Seed aril absent (3:0), seeds yellow-brown (4:0) or brown (4:1), ovate (5:0), large (6:3), keel absent (7:0), without notch (8:0), not ridged (9:0), surface smooth (10:0), testa thin (17:0), without stalactites (18:0)
	Sesuvieae	3	55	Worldwide in tropics and subtropics	Presence of seed aril (3:1); seeds black (4:3), rarely red (4:2), depressed-roundish or reniform (5:2); usually not keeled (7:0)

Seeds are also morphologically diverse in Aizoaceae. Their shape, size, and color are the most common traits used to distinguish both genera and species (e.g., Bittrich, 1986; Gerbaulet, 1997; Sukhorukov et al., 2018a). Some have evolved a funicular aril tightly adjoining the seed coat (Müller, 1909; Dnyansagar and Malkhede, 1963; Prakash, 1967). This feature is found in only three genera of Sesuvioideae: *Sesuvium* L. (incl. *Cypselea* Turpin), *Trianthema* L., and *Zaleya* Burm.f. (Hassan et al., 2005; Bohley et al., 2017; Sukhorukov et al., 2018a), whereas other Aizoaceae taxa lack it. The ultrasculpture of their surfaces has been studied by scanning electron microscopy (SEM) in a few genera (Sesuvioideae: Hassan et al., 2005; Hartmann et al., 2011; Sukhorukov et al., 2017; Sukhorukov et al., 2018a; Sukhorukov et al., 2021c; Aizooideae: Chinnock, 1983; Hassan et al., 2005; Mesembryanthemoideae: Bittrich, 1986; Kapranova, 1991; Ruschioideae: Klak, 2000; Earlé and Young, 2020). Their anatomy remains poorly studied, and limited data are available about *Mesembryanthemum cordifolium* L.f., Mesembryanthemoideae (Netolitzky, 1926, as *Aptenia cordifolia* (L.f.) Schwantes), *Tetragonia tetragonoides* (Pall.) Kuntze, Aizooideae (Netolitzky, 1926 as *T. expansa* Murray), and about many Sesuvioideae [*Zaleya pentandra* (L.) C.Jeffrey (Narayana, 1962, as *Trianthema pentandra* L.), *Trianthema portulacastrum* L. (Kapranova, 1991)] and many *Sesuvium* representatives (Sukhorukov et al., 2018a; Sukhorukov et al., 2019a). Studies on the development of ovules and seeds were conducted on *Mesembryanthemum crystallinum* L. (Mesembryanthemoideae) by Woodcock (1930).

Considering the enormous morphological diversity of the family and their different ecological preferences, detailed comparative analyses of their seeds would be highly desirable, probably revealing many additional implications for their evolution, ecology, and taxonomy. The aims of this study were: (1) to provide new data on the morphology, ultrasculpture, and anatomy of seeds for many taxa across the entire Aizoaceae; (2) to identify novel characteristics for the circumscription of major clades; of particular interest here are unique and complex dispersal mechanisms by water and wind; (3) to investigate the evolution of these dispersal modes, changes in seed and fruit characteristics, especially a correlation between fruit hygrochasy and some seed characteristics, and to trace them on a phylogenetic tree to determine whether different dispersal modes may have been facilitated by other functionally related characteristics; and (4) to understand evolutionary patterns in the ecology of Aizoaceae, using phylogeny to analyze the relation between the structure of the seed coat and habitat preferences of the species.

## 2 Materials and methods

### 2.1 Carpological examination

The capsules and fruits with mature seeds were mostly obtained from the authors’ collections housed in MW, LE, and BOL. Some samples were taken (with permission) from herbarium specimens and others from the Jerusalem Botanical Garden (Israel). The list of studied species is given in **Supplementary File 1**. Seed anatomy was investigated by preparing cross-sections on a Microm HM 355S rotary microtome (Thermo Fisher Scientific, USA). Before the sectioning, the seeds were soaked in a water:alcohol:glycerin (1:1:1) mixture, dehydrated in a graded series of ethanol solutions, and embedded in Technovit 7100 resin (Heraeus Kulzer, Germany). The cross-sections were examined by means of a Nikon Eclipse Ci microscope and photographed with a Nikon DS-Vi1 camera. Ultrasculpture of seeds was studied in the Laboratory of Electron Microscopy at M.V. Lomonosov Moscow State University, using a scanning electron microscope JSM–6380 (JEOL Ltd., Japan) at 15 kV after sputter coating with gold-palladium. For all carpological methods, two to four seeds (or fruits if they are indehiscent) were used. For choosing the minimum-maximum value of quantitative characters 6 and 17 (see below), we follow the methods described by (Zaika et al., 2020, with references therein). Fruit hygrochasy was checked by all species under study (**Supplementary File 2**), and it should apriori depend on seed number per fruit and seed-coat thickness.

Eighteen characters were distinguished in the present study. Three or more states are recognized for most of the characters.

1. The number of seeds in each fruit: 0, three or more; 1, one (indehiscent fruit); 2, a variable number (1 or 2); 3, two.
2. Fruit mericarps: 0, absent; 1, present.
3. Seed aril: 0, absent; 1, present.
4. Seed color: 0, yellowish-brown; 1, brown; 2, red; 3, black or reddish-black; 4, bicolored (pink and white).
5. Seed shape: 0, ovate; 1, depressed-roundish; 2, kidney-shaped (reniform); 3, D-shaped; 4, pear-shaped.
6. Seed size: 0, very small (0.3–0.7 mm); 1, small (0.8–1.1 mm); 2, medium (1.2–1.7 mm); 3, large (from 1.8 mm).
7. Seed keel: 0, absent; 1, present.
8. Seed notch: 0, absent or small; 1, clearly visible, reaching ^1^/_3_–^1^/_2_ of the diameter of the seed [if present, this notch looks like a stripe lacking prominent architecture and is located near the funicle toward the center of the seed].
9. Longitudinal ridges on the seed: 0, absent; 1, present on the whole surface; 2, present on the margin along the cotyledon.
10. Surface ultrasculpture of the seed: 0, smooth; 1, rugose; 2, colliculate (at least in some parts); 3, mamillate; 4, cristate; 5, finger-like.
11. Puzzle-like borders of testa cells: 0, absent or not visible; 1, present.
12. The surface of testa cells: 0, not foveolate; 1, foveolate (with small pits).
13. Waxes on the seed surface: 0, absent or scant; 1, present.
14. Shape of wax deposits: 0, flakes; 1, finger-like; 2, crystals; 3, not applicable due to absence of waxes.
15. Perisperm: 0, scanty; 1, present.
16. Embryo shape: 0, annular; 1, from straight to r-shaped; 2, bent.
17. Thickness of the seed-coat testa: 0, very thin (up to 20 µm); 1, medium (20–40 µm); 2, thick (more than 40 µm).
18. The seed-coat testa: 0, without stalactites; 1, with dark stalactites.

The states for each species (including the outgroups) were coded for reconstructions of ancestral characters and are available in the **Supplementary File 2**.

Among all the investigated characteristics, gross morphological characters 1–7 are well known. Micromorphological traits (8–18) were investigated to enrich our knowledge about the surface and anatomy of seeds, the presence of perisperm, and the shape of embryos.

### 2.2 Phylogenetic analyses

For most species used in our carpological analysis, sequence data could be retrieved from GenBank. In cases where sequences were not available, we substituted sequences of very similar species from the same genus (**Supplementary File 3**). Because we were interested only in major trends of evolution of seed characters across the entire family, we assumed that these characters are likely to be similar within a genus. The names in our tree are the species used to reconstruct the phylogeny. The relationships between the subfamilies and tribes were shown to be the same if only nuclear markers (Thiede, 2004; Thiede et al., 2007) or only chloroplast data were used (Klak et al., 2003). Analysis of sequences of the nuclear gene ‘chloroplast-expressed glutamine synthetase’ (ncpGS) as well as ITS revealed extensive paralogy within the larger clades (Klak et al., 2013; Klak et al., 2017b). We therefore used sequences only from the following four chloroplast gene regions: the *trnS*-*trnG* intergenic region (Hamilton, 1999), the *trn*L-F region (consisting of the adjacent *trn*L intron and *trn*L-F intergenic spacer) (Taberlet et al., 1991), the *rps16* gene region (Oxelman et al., 1997) and the *rpl16* intron (Jordan et al., 1996; Kelchner and Clark, 1997). The latter three gene regions were found to be both variable enough to obtain resolution and are conserved enough to be aligned across the entire Aizoaceae. In addition, we added the highly variable *trnS*-*trnG* intergenic region to provide additional resolution within the Ruschieae (Klak et al., 2013). Due to the lack of suitable sequence data, very species-rich genera *Antimima* N.E.Br., *Ruschia* Schwantes, *Drosanthemum* Schwantes, *Delosperma* N.E.Br., and *Lampranthus* N.E.Br. were each represented only by at most three species in the phylogenetic analysis, although we sampled more species for the carpological analysis. Sequences were aligned by eye.

The combined chloroplast data was investigated by means of RaxML (Stamatakis, 2006) and CIPRES Portal version 2.2 (Miller et al., 2010), which yielded a maximum likelihood tree with bootstrap support for its nodes.

### 2.3 Ancestral-state reconstructions and dispersal systems

Morphological information was extracted from our new data, from our field records made over several seasons, and from herbarium specimens. We used a consensus tree for the ancestral reconstruction of characters, with Fitch parsimony as implemented in the Ancestral State Reconstruction Package in Mesquite version 3.04 (Maddison and Maddison, 2009). Characters were treated as unordered. For those species replaced in our tree by closely related species from the same genus, we employed the results of our investigation to code the characters. With respect to hygrochasy and the number of seeds per capsule, we confirmed that substituted species in the phylogeny had states identical to those in the species investigated in our study. The only discrepancy was probably in the case of the thickness of the seed-coat testa (character 17), where we found considerable variation among species.

We reconstructed three characters: hygrochasy, the number of seeds per fruit (character 1), and the thickness of the seed-coat testa (character 17). We coded hygrochasy as absent (0), present (1), and with a rapid response to soaking [valves (fruit parts) clearly open after soaking] or present (2), but valves open only very slightly because expanding tissue in the fruits is much reduced.

### 2.4 Statistical analysis

One hundred thirty-two species of Aizoaceae were classified by the paired group (UPGMA) algorithm of cluster analysis constructed on a Gower similarity matrix (Gower, 1971) based on a distribution of 18 morphological and anatomical seed characteristics (**Supplementary File 2**). This approach allows one to recognize the species that group on the basis of similarly evolved characters but it does not provide a true phylogenetic context. Principal component analysis was applied to ordinate species in the space of seed characters. Ordination diagram allows to assess not only resemblance between different species by seed morphological characteristics but also to distinguish those seed characteristics that affect most of the variation among Aizoaceae and to relate them to specific groups of species.

In our previous papers, multivariate analyses provided good support for a nonstochastic distribution of the characteristics in major clades of the entire Molluginaceae (Sukhorukov et al., 2018b), of the genus *Microtea* Sw., Microteaceae Schäferh. & Borsch (Sukhorukov et al., 2019c), and of the genus *Glinus* L. (Sukhorukov et al., 2021b).

To assess the relation between the hygrochastic fruit and two characteristics of seeds, namely size and thickness of the coat testa, we calculated the significance of differences in seed size and seed coat testa thickness between species with and without hygrochasy by the Mann–Whitney test.

Calculations and visualization were performed in PAST version 4.11 (Hammer et al., 2001).

## 3 Results

### 3.1 Seed characters that define larger clades in Aizoaceae

Our work revealed high diversity of seed characteristics defining major taxonomic groups in Aizoaceae (see **Table 1** and **Supplementary File 2**). Below we highlight the main findings about the 18 characteristics we investigated.

**Figure 2:**
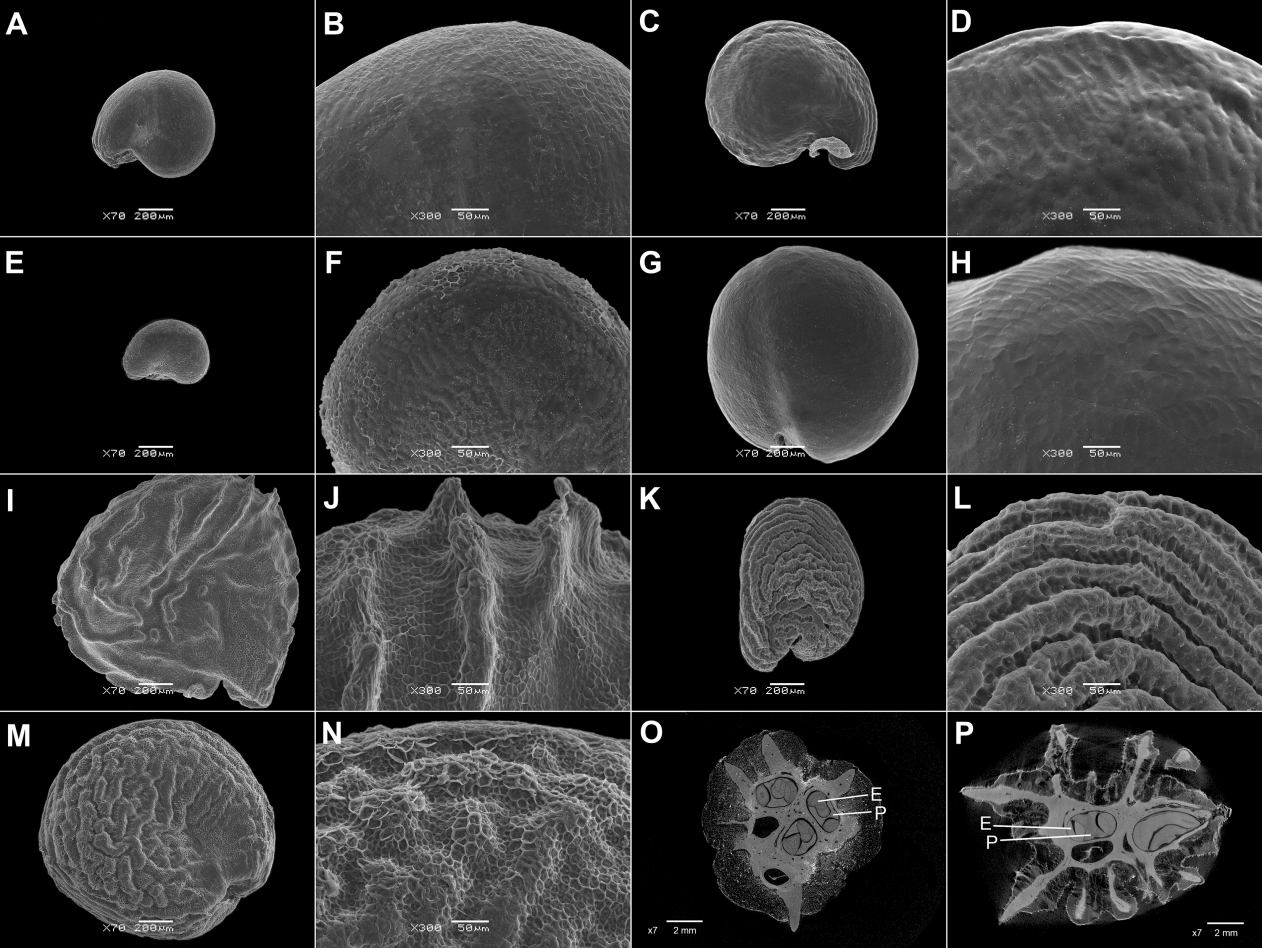
SEM micrographs or tomograms of selected Sesuvioideae species. **(A, B)** A seed of *Sesuvium maritimum* (covered with an aril), **(C, D)** a seed of *Sesuvium curassavicum*, **(E, F)** a seed of *Sesuvium mezianum* (covered with an aril), **(G, H)** a seed of *Trianthema hereroense*, **(I, J)** a seed of *Trianthema portulacastrum* (covered with an aril), **(K, L)** a seed of *Trianthema crystallinum* (covered with an aril), **(M, N)** a seed of *Zaleya pentandra* (covered with an aril), **(O)** a tomogram of a *Tribulocarpus somalensis* fruit with three visible seeds, and **(P)** a tomogram of a *Tribulocarpus dimorphanthus* fruit with two visible seeds. Magnification: **(A, C, E, G, I, K, M)** 70×; **(B, D, F, H, J, L, N)** 300×; **(O, P)** 7×. P, perisperm; E, embryo.

**Figure 3:**
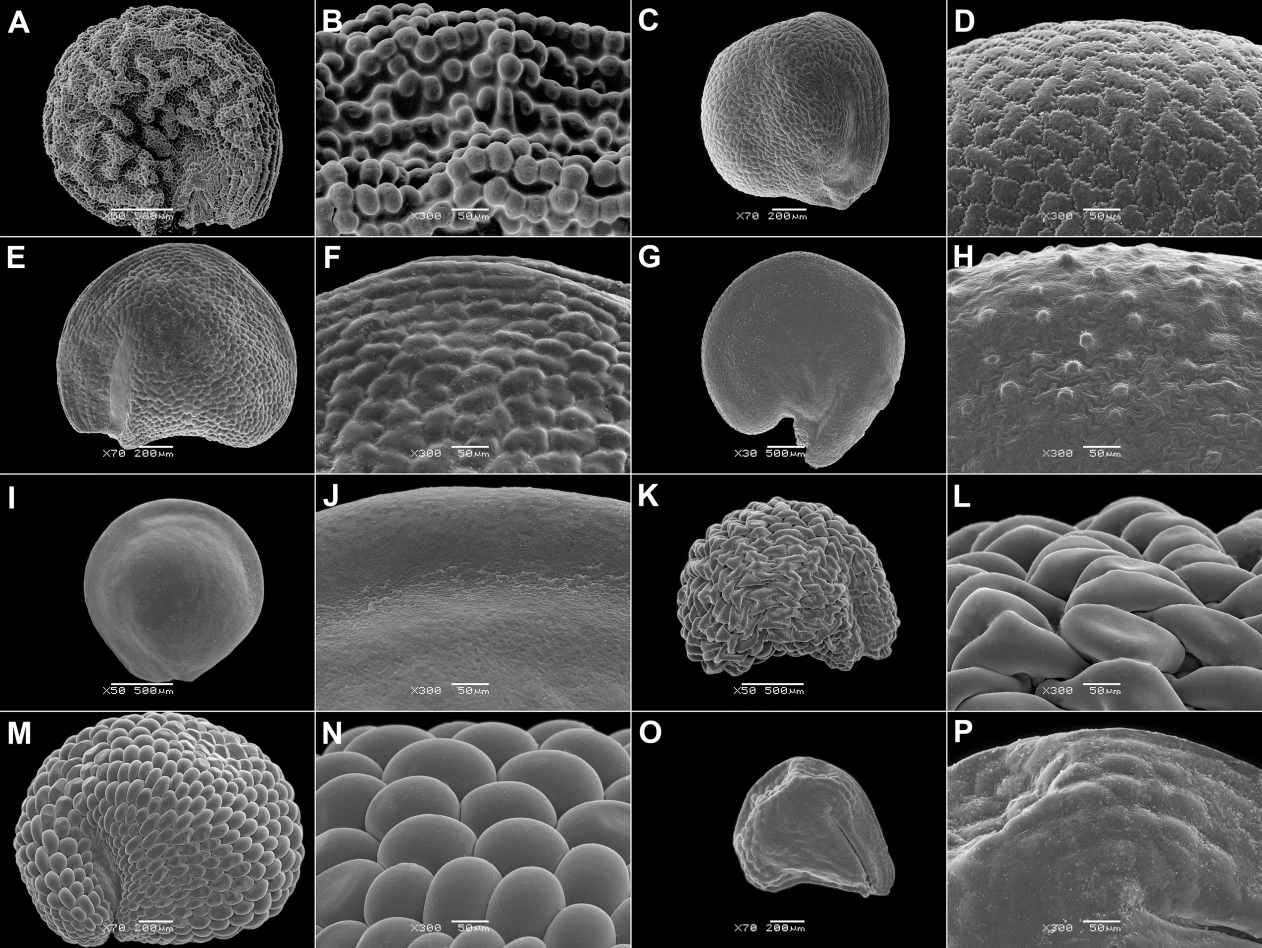
SEM micrographs of seeds of selected Acrosanthoideae and Ruschioideae (Apatesieae, Dorotheantheae) species. **(A, B)** *Acrosanthes anceps*, **(C, D)** *Apatesia helianthoides*, **(E, F)** *Carpanthea pomeridiana*, **(G, H)** *Hymenogyne glabra*, **(I, J)** *Conicosia elongata*, **(K, L)** *Skiatophytum tripolium*, **(M, N)** *Cleretum bruynsii*, and **(O, P)** *Cleretum bellidiforme*. Magnification: **(A, I, K)** 50×; G: 30×; **(C, E, M, O)** 70×; **(B, D, E, H, J, I, N, P)** 300×.

**Figure 4:**
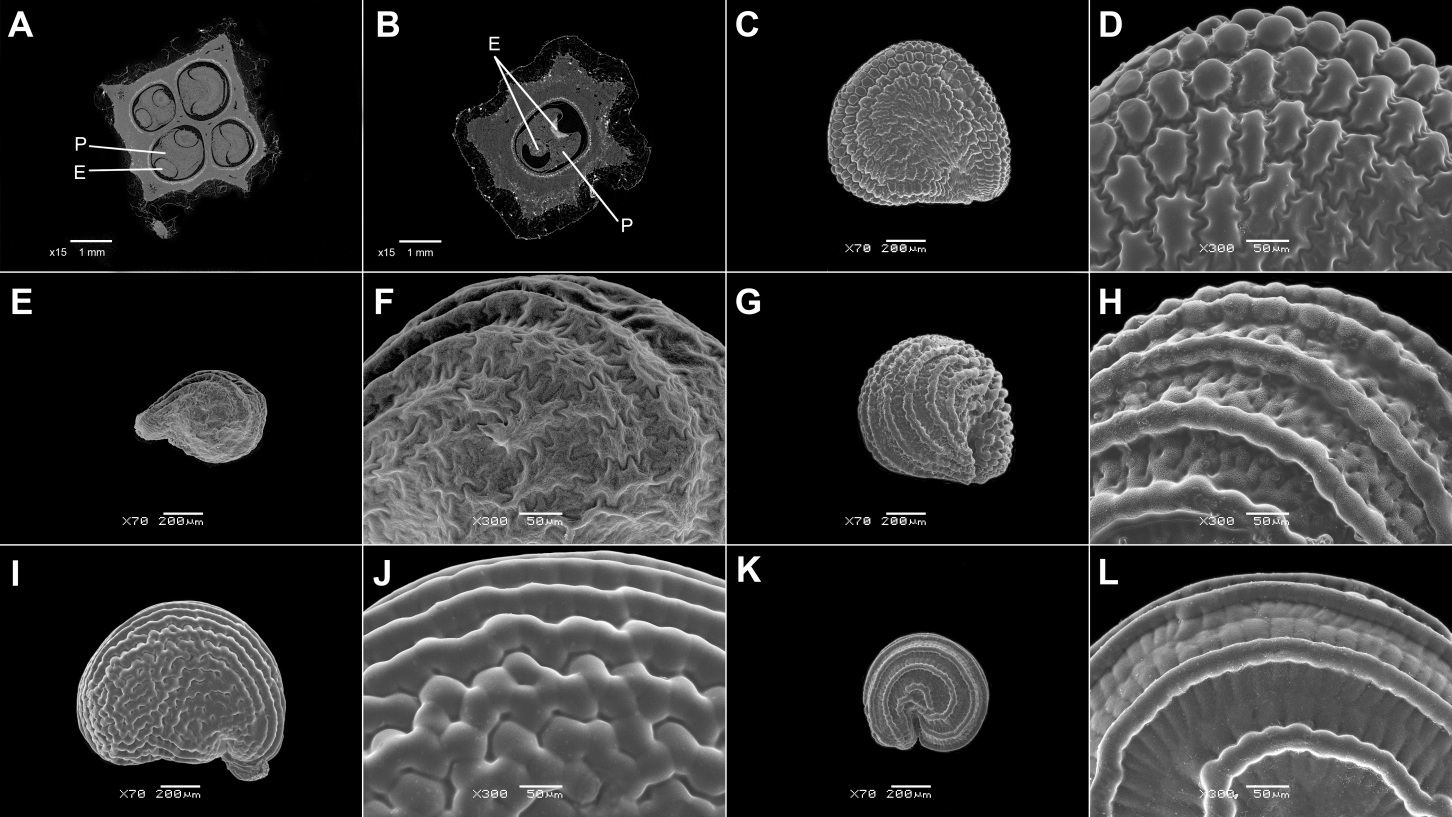
SEM micrographs or tomograms of selected Aizooideae species. **(A)** A tomogram of a *Tetragonia echinata* fruit with four visible seeds, **(B)** a tomogram of a *Tetragonia saligna* nut-like fruit, **(C, D)** a seed of *Gunniopsis papillata*, **(E, F)** a seed of *Gunniopsis calva*, **(G, H)** a seed of *Aizoanthemopsis hispanica*, **(I, J)** a seed of *Aizoon paniculatum*, and **(K, L)** a seed of *Aizoanthemum mossamedense*. Magnification: **(A, B)** 15×; **(C, E, G, I, K)** 70×; **(D, F, H, J, L)** 300×. P, perisperm; E, embryo.

**Figure 5:**
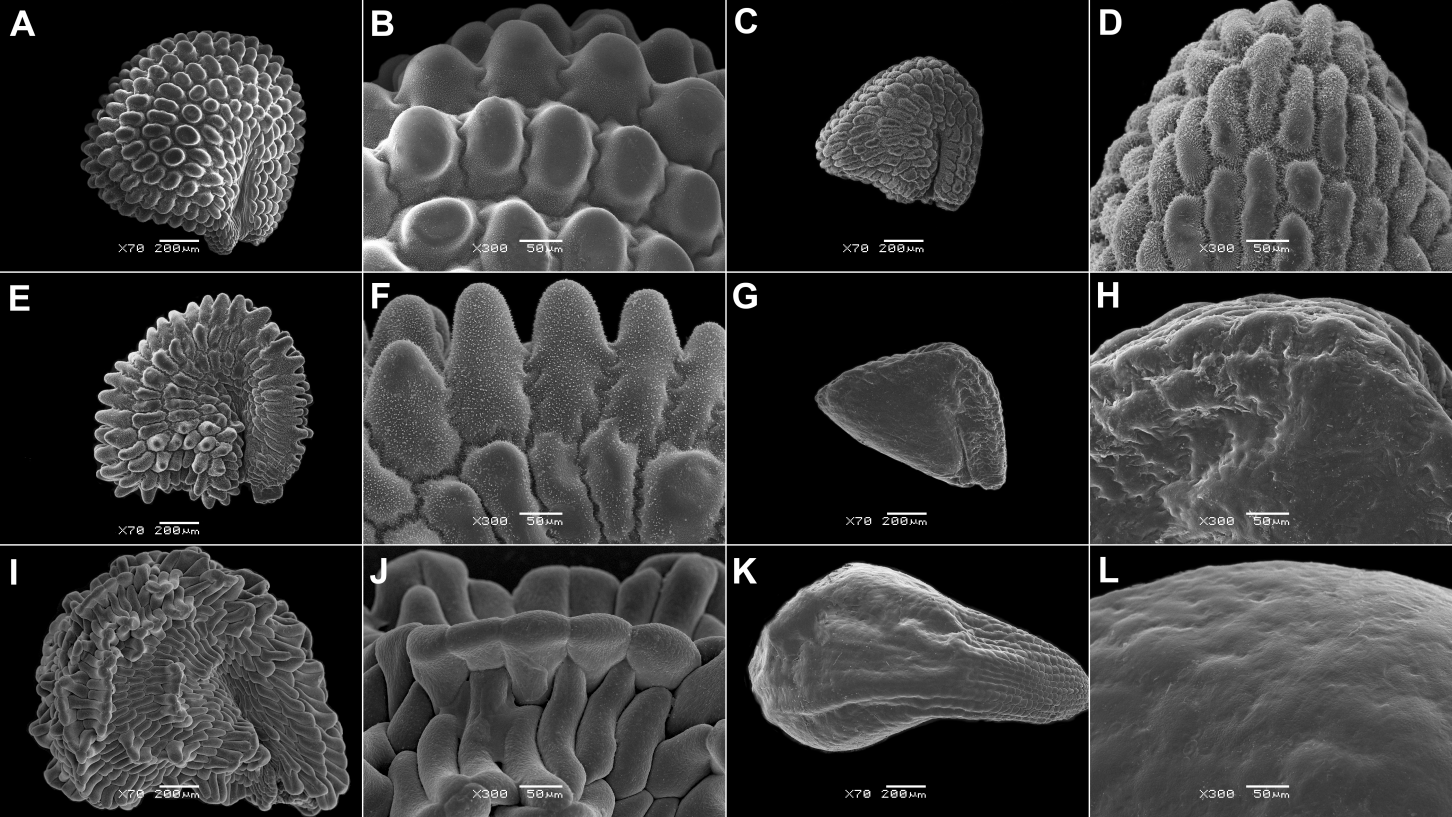
SEM micrographs of seeds of selected Mesembryanthemoideae species. **(A, B)** *Mesembryanthemum cordifolium*, **(C, D)** *M. aitonis*, **(E, F)** *M. lignescens*, **(G, H)** *M. nodiflorum*, **(I, J)** *M. rabiei*, and **(K, L)** *M. nucifer*. Magnification: **(A, C, E, G, I, K)** 70×; **(B, D, F, H, J, L)** 300×.

**Figure 6:**
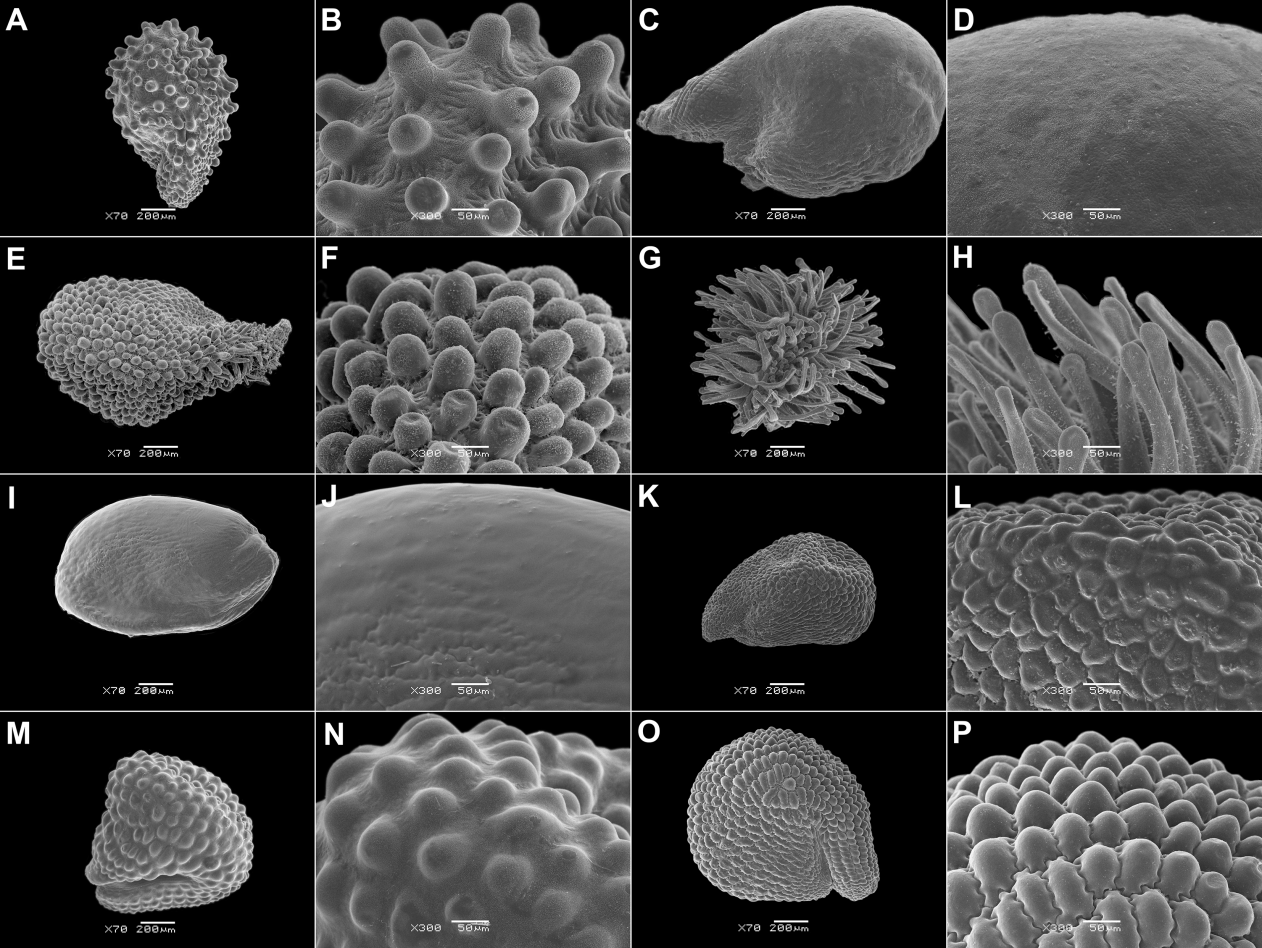
SEM micrographs of seeds of selected Ruschioideae-Ruschieae species. **(A, B)** *Acrodon parvifolius*, **(C, D)** *Antimima ventricosa*, **(E, F)** *Astridia longifolia*, **(G, H)** *Braunsia geminata*, **(I, J)** *Carpobrotus edulis*, **(K, L)** *Cheiridopsis peculiaris*, **(M, N)** *Circandra serrata*, and **(O, P)** *Cylindrophyllum hallii*. Magnification: **(A, C, E, G, I, K, M, O)** 70×; **(B, D, F, H, J, L, M, P)** 300×.

1. Most Aizoaceae have multi-seeded fruits (**Figure 1**; **Supplementary File 2**). A reduction in the number to one or to a variable number (1–2) occurred several times independently across Aizoaceae. Two-seeded fruits are predominantly found in *Trianthema* L. and *Zaleya* Burm.f. (Sesuvioideae-Sesuvieae).

2. A special case of multi-seeded fruits is the unique type where the fruit consists of one- or two-seeded mericarps. These are found only in Apatesieae and in *Ruschianthemum gigas* (Dinter) Friedrich (Ruschieae), both belonging to Ruschioideae.

3. A one- to multilayered parenchymatous seed aril is present only in *Sesuvium* L., *Trianthema*, and *Zaleya* (Sesuvioideae-Sesuvieae; **Figure 2**; **Supplementary File 4**). The aril is absent in the seeds from synaptospermic fruits like the burrs of *Tribulocarpus dimorphanthus* (Pax) S.Moore and *T. somalensis* (Engl.) Sukhor. (Sesuvioideae-Anisostigmateae).

4. Yellowish-brown, brown, and rarely red seeds are most widespread in Mesembryanthemoideae and some Ruschioideae members. Black or reddish-black seeds are common in almost all Sesuvioideae-Sesuvieae (*Trianthema*, *Zaleya*, many *Sesuvium*), some Aizooideae (*Aizoon* L., *Aizoanthemum* Dinter ex Friedrich, and *Aizoanthemopsis* Klak), and Ruschioideae (Apatesieae, rarely some Ruschieae, e.g., *Ruschia caroli* (L.Bolus) Schwantes, *R. dichroa* (Rolfe) L.Bolus, and *Lampranthus watermeyeri* (L.Bolus) Schwantes). Bicolored seeds are found only in *Drosanthemum dejagerae* L.Bolus. Seeds in synaptospermic burrs (*Tribulocarpus* S.Moore), indehiscent fruits (*Tetragonia* L.), and nuts (except *Aizoon fruticosum* L.f.) are yellow or brown.

5. Ovate seeds have evolved rarely. Remarkably, this state is also present in the unusual fruits dispersed *via* anemochory or zoochory [as in synaptospermic diaspores of *Tribulocarpus* (Sesuvioideae-Anisostigmateae, **Figure 1B**), soft fruits of *Carpobrotus* N.E.Br. (Ruschioideae, **Figure 1E**), and in winged diaspores of *Anisostigma* Schinz (Sesuvioideae-Anisostigmateae, **Figure 1A**)]. Depressed-roundish seeds are also rare and have evolved in some Sesuvioideae (*Trianthema*; **Figures 2G, I, K**) and Ruschioideae (Apatesieae; **Figure 3C**). Kidney-shaped seeds are most common in *Sesuvium* (Sesuvioideae; **Figures 2A, C, E**) and *Aizoon*, *Aizoanthemum*, and *Aizoanthemopsis* (Aizooideae; **Figure 4**; **Supplementary File 4**). In contrast, D-shaped seeds are usual in Mesembryanthemoideae (**Figure 5**) and in some Ruschioideae-Ruschieae. Pear-shaped seeds seem to be specific to Ruschioideae (Ruschieae and Drosanthemeae; **Figure 6**). Nonetheless, pear-shaped seeds are also uniquely present in *Mesembryanthemum nucifer* (Ihlenf. & Bittrich) Klak (Mesembryanthemoideae).

6. Seed size varies significantly. Very small seeds are typical of Ruschioideae (**Figure 6**; **Supplementary Files 4**, **5**). Three *Sesuvium* species (*S. humifusum* (Turpin) Bohley & G.Kadereit, *S. mezianum* (K.Müll.) Bohley &G.Kadereit, and *S. rubriflorum* (Urb.) Bohley & G.Kadereit, formerly in *Cypselea*) also have tiny seeds (0.3–0.4 mm long): an unusual characteristic for Sesuvioideae. The largest seeds are found in Sesuvioideae-Anisostigmateae and in Ruschioideae-Apatesieae (**Supplementary Files 2**, **4**).

7. Keeled seeds are very rare and are found in *Trianthema hereroense* Schinz (**Figure 2G**), *T. triquetrum* Willd. (Sesuvioideae), *Conicosia elongata* (Haw.) N.E.Br., and *Hymenogyne glabra* (Aiton) Haw. (the latter two are both in Ruschioideae-Apatesieae; **Figures 3G, I**). In almost all cases, the keel is present in red or black seeds, which have a more robust seed coat (>20 μm thick).

8. A seed notch is found in almost all Mesembryanthemoideae (except *M. nucifer*) and many Ruschioideae but is absent in Acrosanthoideae, Aizooideae, and Sesuvioideae (**Supplementary Files 4**, **5**; **Figures 3**, **5**, **6**). It has an appearance of a stripe without prominent sculpturing and is located near the funicle toward the center of the seed.

9. Only a few species have ridges on the surface of their seeds. Ridges located only at the margin were found in *Gunniopsis* Pax, whereas other Aizooideae (*Aizoon*, *Aizoanthemum*, and *Aizoanthemopsis*) have ridges covering almost the entire surface (**Figure 4**). Small ridges on the surface have independently evolved in *Acrosanthes* Eckl. & Zeyh. (Acrosanthoideae) and many *Trianthema* (Sesuvioideae).

10. Smooth seeds are always found in indehiscent diaspores: obligatory one-seeded (*Anisostigma*) and synaptospermic (*Tribulocarpus* and *Tetragonia*) fruits where all the diaspore’s covers tightly adhere to each other. They are also present in *Conicosia* (Apatesieae, Ruschioideae; **Figures 3I, J**), which has one-seeded mericarps. Among species with multi-seeded capsules, smooth seeds are mostly found in Ruschieae (Ruschioideae) and correlate with the small size of the seeds (see character 6) and a thin seed coat (see character 17). Rugose surfaces are rare. Nevertheless, colliculate and papillate surfaces are common, especially in Mesembryanthemoideae (**Figure 5**) and Ruschioideae-Ruschieae (**Figure 6**; **Supplementary File 5**) with red or reddish-black seeds. Cristate and finger-like outgrowths have evolved several times, but only within Mesembryanthemoideae (**Figures 5F, J**) and Ruschioideae.

11. Puzzle-like borders of testa cells are common in Mesembryanthemoideae and Ruschioideae, especially in brown, red, or black seeds (**Supplementary Files 2**, **4**). They are also present in *Gunniopsis* (Aizooideae; **Figures 4D, F**) but not in other members of this subfamily.

12. A foveolate surface of the testa is exceptionally rare. It is found in some Aizooideae (*Aizoon*, *Aizoanthemum*, and *Aizoanthemopsis*), *Mesembryanthemum rabiei* (L.Bolus) Klak (Mesembryanthemoideae; **Figure 5J**), and *Acrosanthes* (Acrosanthoideae; **Figure 3B**). In species with finger-like cell projections, e.g., in *Glottiphyllum* N.E.Br., this character cannot be assessed precisely.

13. Waxes are mostly present on the seed surface in Mesembryanthemoideae and Ruschioideae, except for Apatesieae. Within Aizooideae, waxes are found in *Gunniopsis* and *Aizoanthemopsis* (**Figure 7**). All synaptospermic fruits (*Tetragonia*, Aizooideae; and *Tribulocarpus*, Sesuvioideae) lack waxes. In other Sesuvioideae, the presence of waxes cannot be strictly confirmed owing to the aril usually tightly covering the seed. Nevertheless, in cases where the aril is brittle and easily removable – and therefore the surface of the seed is clearly visible (e.g., in *Sesuvium portulacastrum* (L.) L.) – no waxes were found.

**Figure 7:**
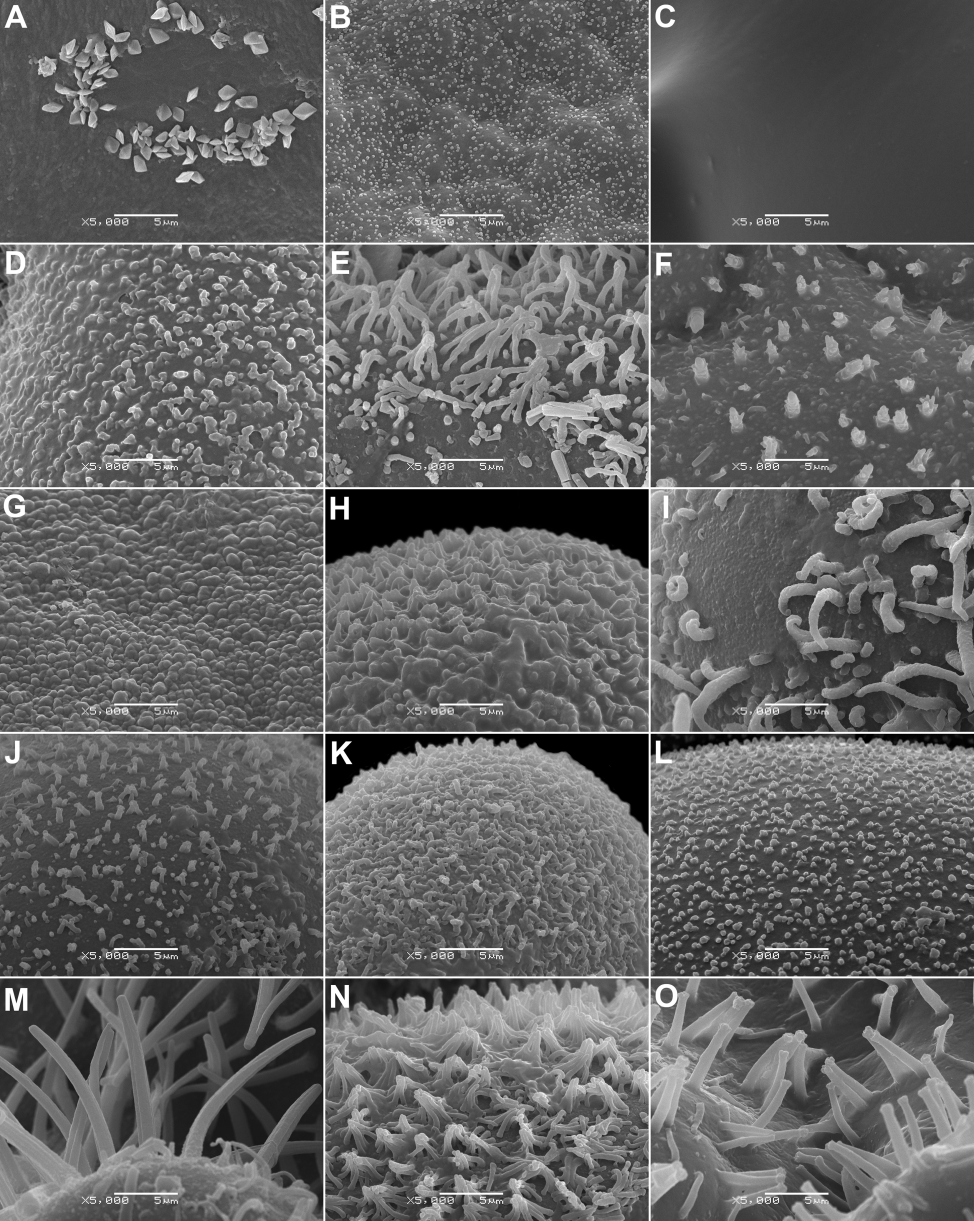
SEM micrographs of a seed surface of selected Aizoaceae species at 5000× magnification showing wax crystals of different shapes or glabrous surface. **(A)** *Gunniopsis papillata* (prismatic crystals), **(B)** *Gunniopsis calva* (flake-like crystals), **(C)** *Aizoon paniculatum* (no wax deposits), **(D)** *Aptenia cordifolia* (flake-like crystals), **(E)** *Mesembryanthemum aitonis* (finger-like crystals), **(F)** *Mesembryanthemum lignescens* (flake-like crystals), **(G)** *Conicosia elongata* (flake-like crystals), **(H)** *Acrodon parvifolius* (flake-like crystals), **(I)** *Astridia longifolia* (finger-like crystals), **(J)** *Disphyma crassifolium* (flake-like crystals), **(K)** *Eberlanzia gravida* (flake-like crystals), **(L)** *Esterhuysenia drepanophylla* (flake-like crystals), **(M)** *Glottiphyllum linguiforme* (finger-like crystals), **(N)** *Khadia acutipetala* (flake-like crystals), and **(O)** *Namaquanthus vanheerdei* (finger-like crystals).

**Figure 8:**
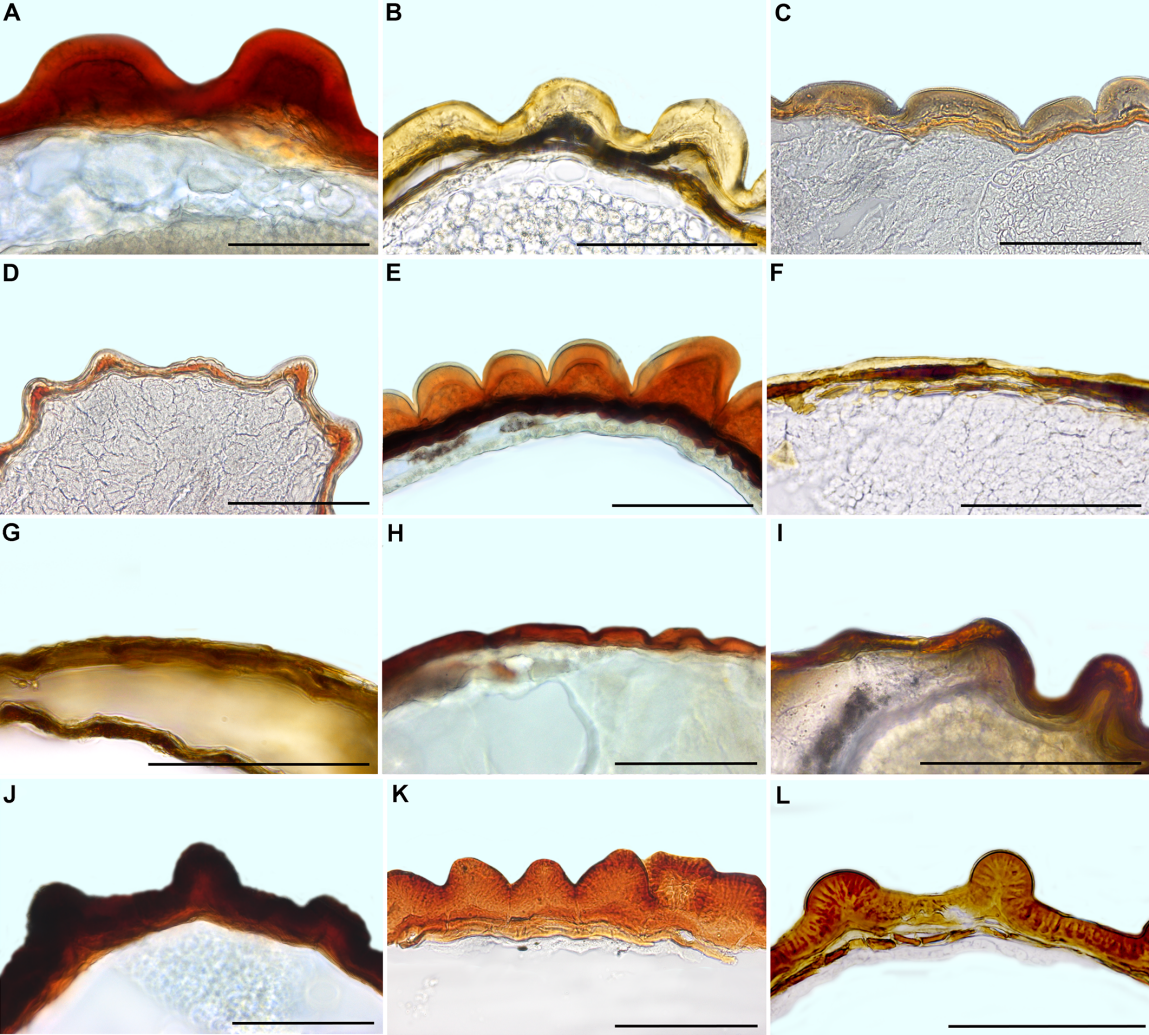
Seed coat cross-sections of selected Mesembryanthemoideae **(A–F)** and Aizooideae **(G–L)** species. **(A)** *Mesembryanthemum cordifolium*, **(B)** *M. aitonis*, **(C)** *M. crystallinum*, **(D)** *M. nodiflorum*, **(E)** *M. rabiei*, **(F)** *M. nucifer*, **(G)** *Tetragonia echinata*, **(H)** *Gunniopsis papillata*, **(I)** *Gunniopsis calva*, **(J)** *Aizoanthemopsis hispanica*, **(K)** *Aizoon paniculatum*, **(L)** *Aizoanthemum mossamedense.* Scale bar – 100 µm.

**Figure 9:**
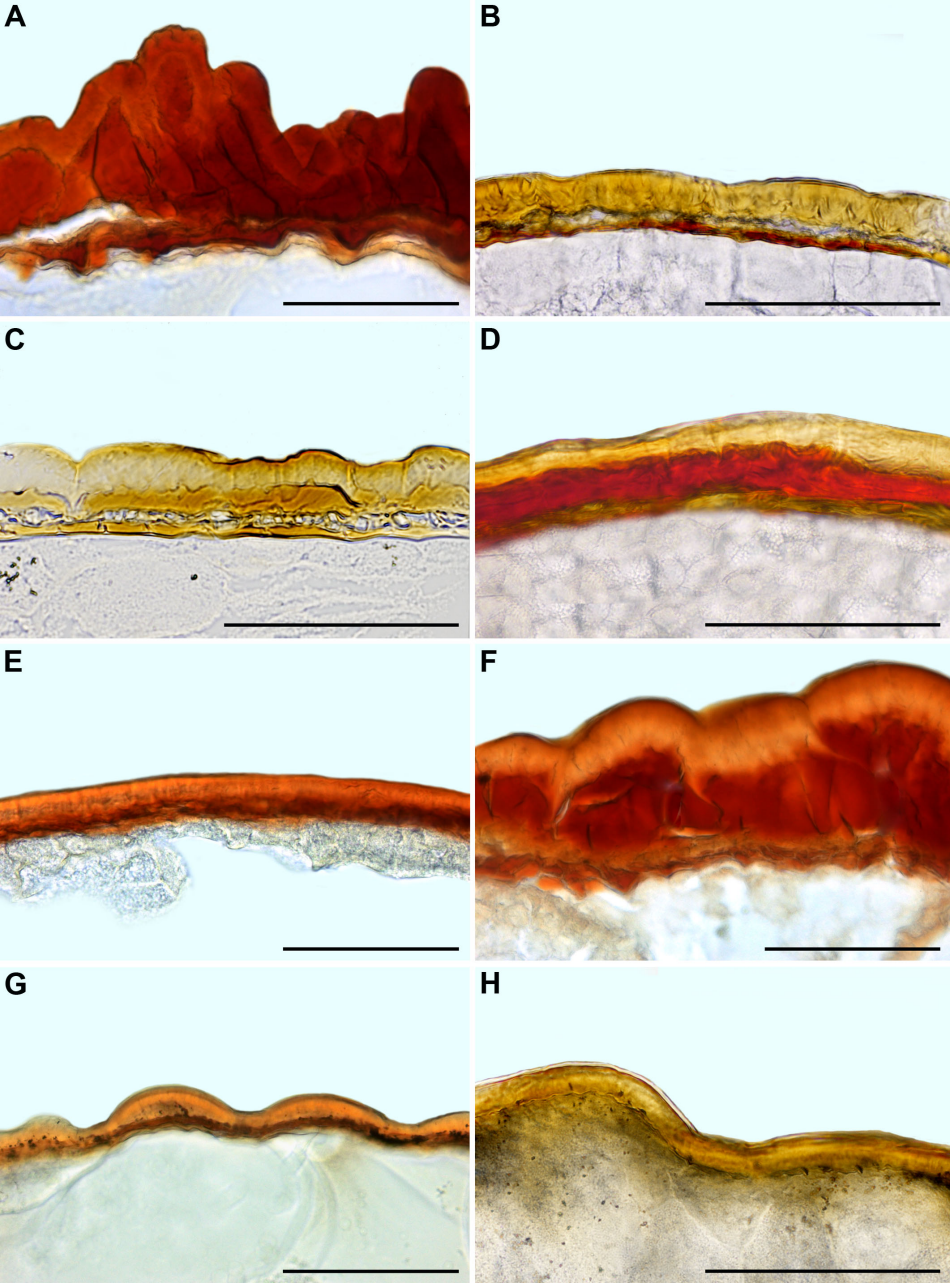
Seed coat cross-sections of selected Acrosanthoideae **(A)** and Ruschioideae **(B–H)** species. **(A)** *Acrosanthes anceps*, **(B)** *Apatesia helianthoides*, **(C)** *Carpanthea pomeridiana*, **(D)** *Hymenogyne glabra*, **(E)** *Conicosia elongata*, **(F)** *Skiatophytum tripolium*, **(G)** *Cleretum bruynsii*, **(H)** *Cleretum bellidiforme*. Scale bar – 100 µm.

**Figure 10:**
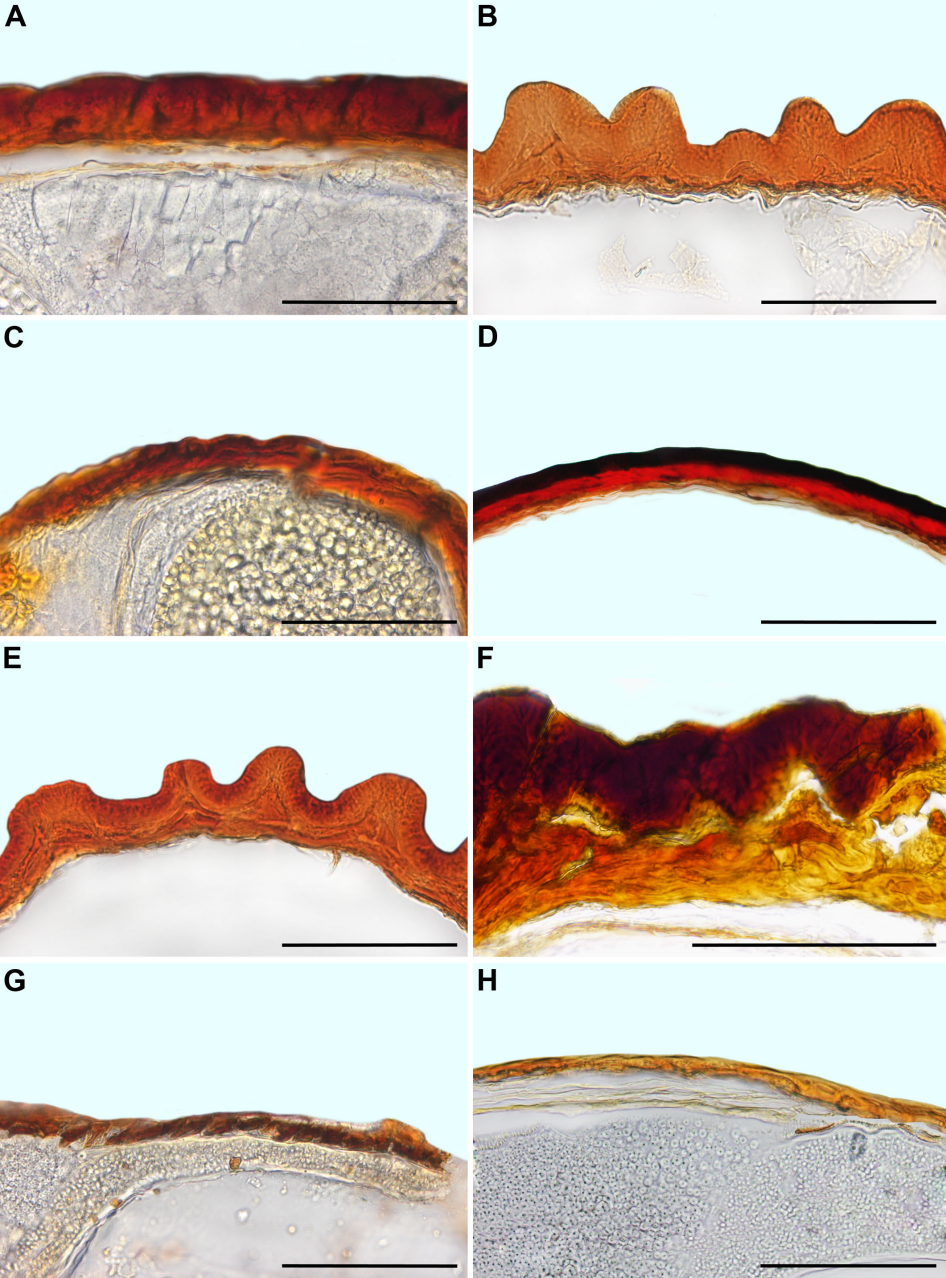
Seed coat cross-sections of selected Sesuvioideae species. **(A)** *Sesuvium maritimum*, **(B)** *Sesuvium curassavicum*, **(C)** *Sesuvium mezianum*, **(D)** *Trianthema hereroense*, **(E)** *Trianthema crystallinum*, **(F)** *Zaleya govindia*, **(G)** *Tribulocarpus somalensis*, **(H)** *Tribulocarpus dimorphanthus*. Scale bar – 100 µm.

14. Wax deposits (if present) are flake-like or finger-like or very rarely crystal-like (only in *Gunniopsis papillata* Chinnock, **Figure 7A**).

15. A perisperm is present in all representatives except *Anisostigma* (Sesuvioideae-Anisostigmateae), which has a very small amount of the perisperm (almost invisible to the naked eye).

16. An annular embryo is found in Acrosanthoideae, Ruschioideae (Apatesieae and Drosanthemeae), Sesuvioideae (*Trianthema*, *Zaleya*, *Tribulocarpus*), and many *Sesuvium* taxa), and some Aizooideae. Mesembryanthemoideae and the rest of Ruschioideae have straight to r-shaped or bent embryos.

17. Thickness of the seed coat/testa shows much variation. Tiny seeds have a thin seed coat, and this trait is common among many Ruschioideae (except Apatesieae) and Mesembryanthemoideae (**Figures 8**, **9**; **Supplementary Files 6**, **7**). Seeds in species with synaptospermic burrs (*Tribulocarpus*, **Figure 1B**), indehiscent multi-seeded fruits (*Tetragonia*, **Figures 1C, D**), and nuts (*Anisostigma*) also have a thin seed coat (**Figures 8**, **10**). The thickness of seed coats with colliculate and finger-like sculpturing shows much variation, and its lower and upper limits are indicated in **Supplementary File 2**. In larger genera, e.g., *Trianthema*, *Mesembryanthemum*, and *Gunniopsis*, the thickness of the seed coat also differs among species.

18. Dark stalactites in outer walls of testa cells are rare. They are found only in some *Trianthema* (Sesuvioideae), *Gunniopsis*, all *Aizoon*, *Aizoanthemum*, *Aizoanthemopsis* (Aizooideae), *Erepsia dunensis* (Sond.) Klak, *Carpobrotus* spp. (Ruschieae, Ruschioideae), and *Apatesia helianthoides* N.E.Br. (Apatesieae, Ruschioideae) (**Supplementary Files 6**, **7**). In Mesembryanthemoideae and Ruschioideae, some representatives possess white fortifications in outer cell walls (stalactite-like substances), which are mentioned in **Supplementary File 2**.

### 3.2 Phylogeny and reconstruction of ancestral characteristics

**Figure 11:**
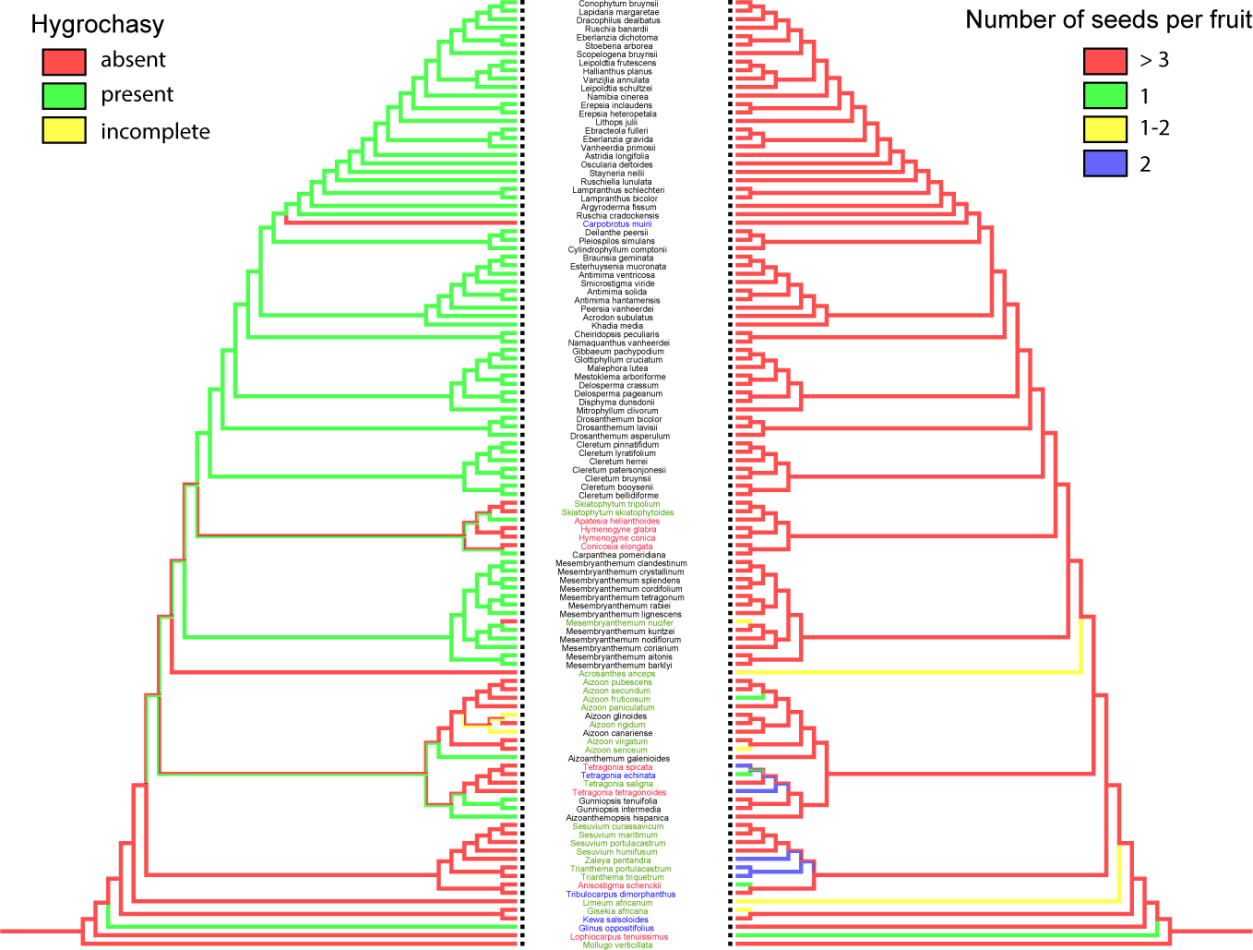
Ancestral character reconstruction of hygrochasy and number of seeds per fruit. Species names were highlighted in different colors indicating their mode of dispersal: black: ombrohydrochory, blue: zoochory, green: autochory, red: anemochory.

In total, we included 345 sequences from four chloroplast gene regions in this analysis (**Supplementary File 3**). The major clades retrieved by the maximum likelihood analysis (**Supplementary File 8**) were congruent with the results of previous molecular studies on Aizoaceae (Klak et al., 2013; Klak et al., 2017a) and will therefore not be discussed here. We used this tree for reconstructions of states of the ancestral characters using the parsimony algorithm.

a. Hygrochasy. The minimum number of changes (using parsimony) for this three-state character is 12 steps (**Figure 11**). The ancestral state is the absence of hygrochasy, which is seen in all members of Sesuvioideae. The reconstruction shows ambiguity regarding multiple gain versus repeated loss of hygrochasy in Aizooideae and Apatesieae (Ruschioideae). On the other hand, for Mesembryanthemoideae and the Dorotheantheae + Ruschieae clade, hygrochasy is the ancestral state with rare loss of hygrochasy in *M. nucifer* (Mesembryanthemoideae) and in *Carpobrotus* (Ruschieae). Relatively few species show reduced hygrochasy, where capsules do not open fully due to much reduced expanding tissue in the valves.

b. The seed number per fruit. The minimum number of changes for this four-state character is 14 steps (**Figure 11**). The ancestral state is multi-seeded fruits, with repeated shifts to one-, variable (1–2), or two-seeded fruits. Sesuvioideae and Aizooideae show the greatest diversity of the number of seeds, whereas Mesembryanthemoideae and Ruschioideae possess almost exclusively multi-seeded fruits, where the seeds are dispersed *via* ombrohydrochory, i.e., are splashed out by raindrops. The seed number is reduced only in xerochastic fruits, which show the highest variation of dispersal modes, i.e., anemochory, zoochory, and autochory.

**Figure 12:**
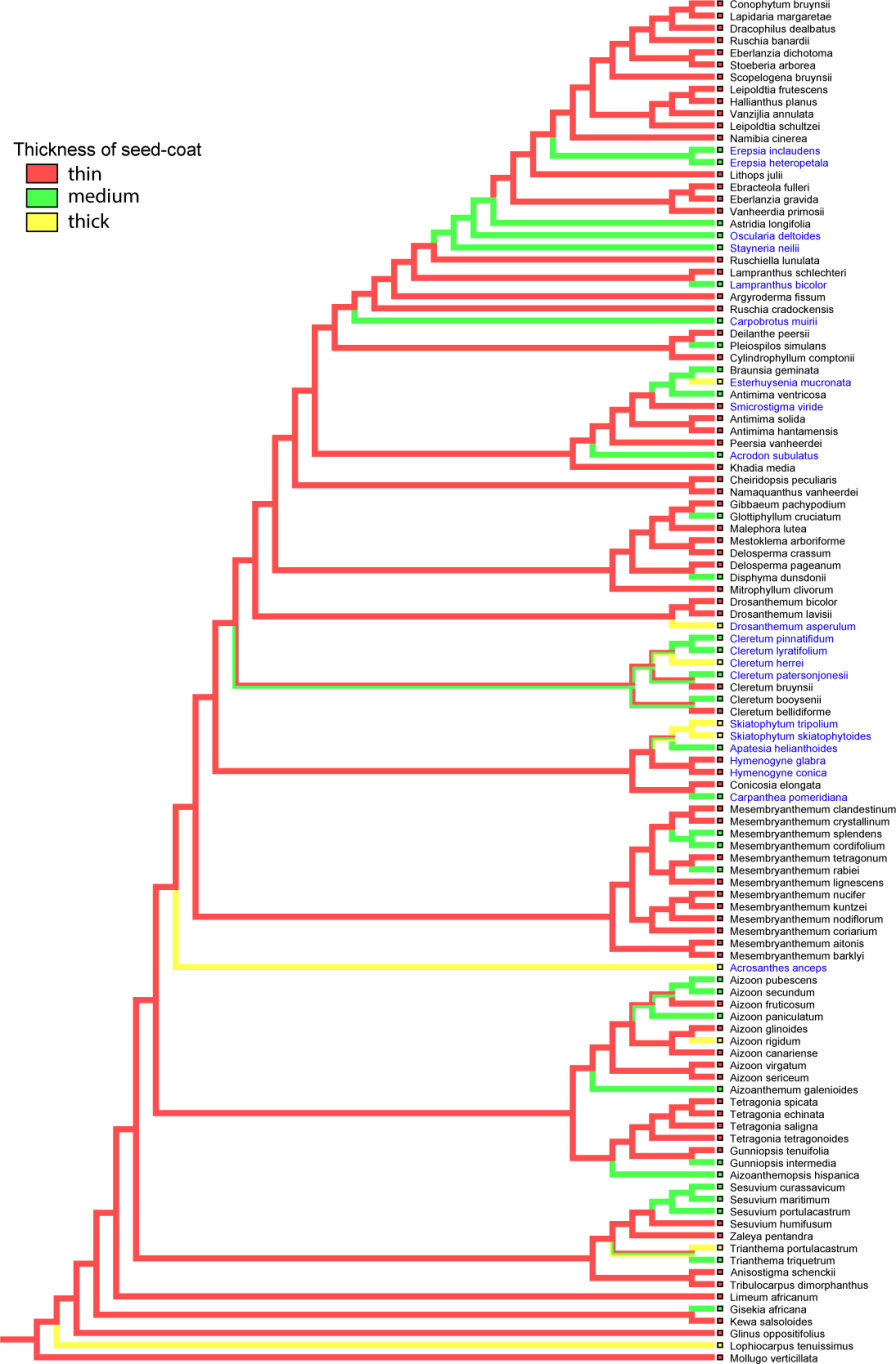
Ancestral character reconstruction of thickness of seed coat. Species names highlighted in blue indicate that they are endemic to a fire prone habitat (either *fynbos* or *renosterveld* vegetation).

Apatesieae constitutes a special case. Here, in several taxa (*Apatesia*, *Hymenogyne*, *Conicosia*, and *Skiatophytum skiatophytoides* (Leistner) Klak), fruits break up into two-seeded mericarps. In Ruschieae, there is only a single species, *Ruschianthemum gigas* (not sampled in this study), where the fruits also break up into one- to two-seeded mericarps. Thus, there have been shifts in all subfamilies towards a drastic reduction in the number of seeds per dispersal unit (fruit or mericarp).

c. Thickness of the seed coat. The minimum number of changes for this three-state character is 33 steps. The large number of steps indicates that this is a variable character (**Figure 12**). A thin seed coat is the ancestral state, with numerous shifts toward a thicker seed coat (medium or thick). In Ruschioideae, thicker seed coat is present in taxa of fire-prone environments, i.e., in *fynbos* and *renosterbos*. Exceptions are *Lampranthus explanatus* (L.Bolus) N.E.Br. and *L. reptans* (Aiton) N.E.Br., which also occur in *fynbos*, but have a thin seed coat.

### 3.3 Variation of morphological characteristics and a correlation with hygrochasy

**Figure 13:**
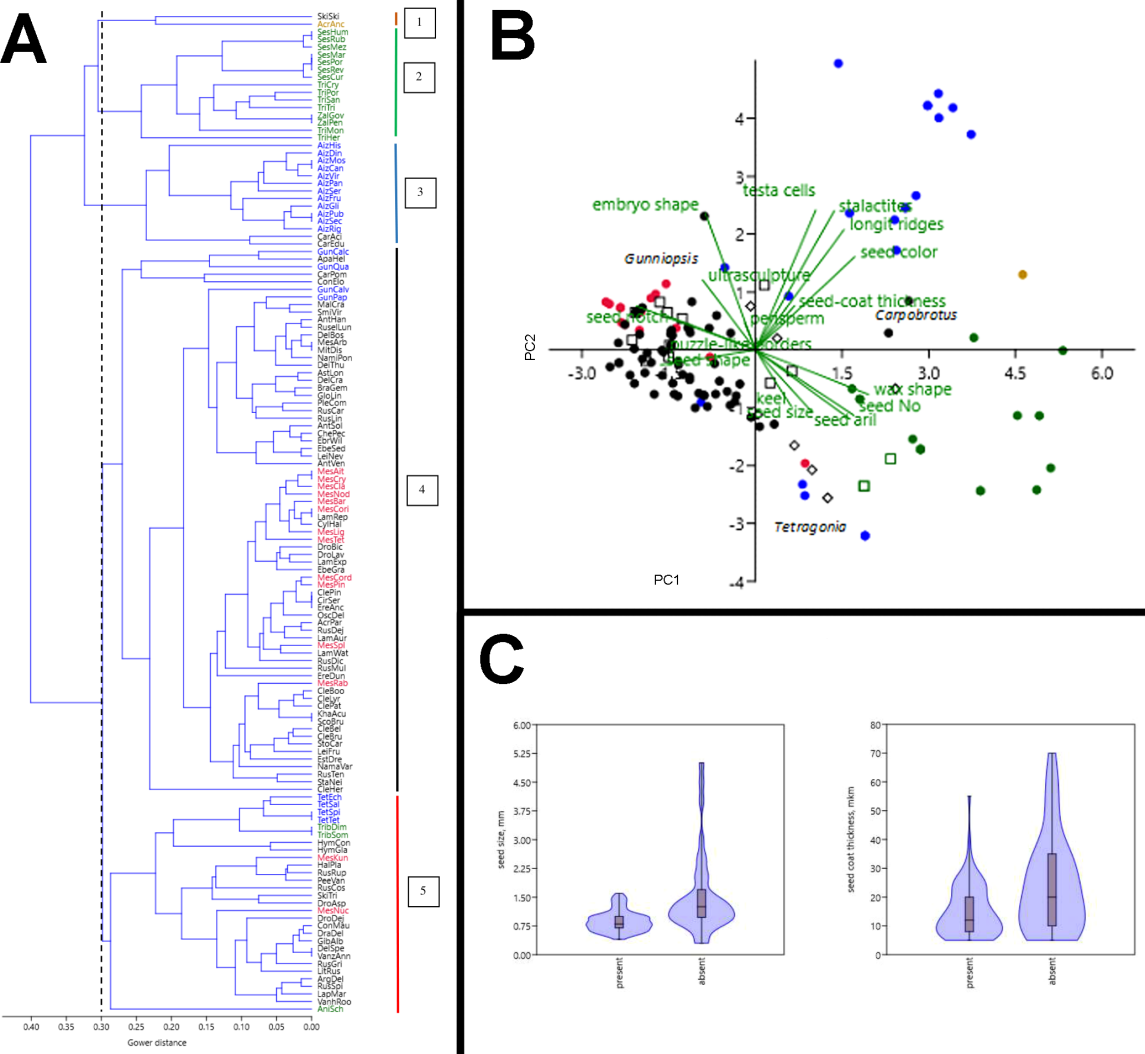
Multivariate analysis of the species examined. **(A)** Classification of Aizoaceae species by the paired linkage algorithm of cluster analysis based on 18 morphological characteristics. The dotted line indicates the highest level of group delimitation. Colored lines on the right mark subgroups (1–5) of species with common morphological seed and fruit characteristics. Name abbreviations are constructed from first letters of generic and species names. Acrosanthoideae are highlighted in brown, Aizooideae in blue, Mesembryanthemoideae in red, Ruschioideae in black, and Sesuvioideae in green. **(B)** Ordination of species based on 18 morphological seed and fruit characteristics by principal component analysis. Brown dot: Acrosanthoideae, blue dots: Aizooideae, red dots: Mesembryanthemoideae, black dots: Ruschieae, black squares: Drosanthemeae, black diamonds: Apatesieae, green dots: Sesuvieae, and green squares: Anisostigmateae. PC 1: first principal component (describes 26.1% of total variance of morphological characteristics of seeds and fruits), PC2: second principal component (13.8%). Genera with a position highly deviating from other representatives of their families are marked as follows: *Tetragonia* (blue dots), *Gunniopsis* (blue dots), and *Carpobrotus* (black dots). **(C)** Differences in seed size (left) and seed-coat thickness (right) between species with hygrochasy (present) and without hygrochasy (absent). Differences in both cases are significant according to the Mann–Whitney test (P < 0.001).

The results of our cluster analysis point to the existence of two major groups with respect to seed and fruit characteristics in Aizoaceae (**Figure 13A**). The largest group consists of two subgroups (subgroups 4 and 5) and includes most of Ruschioideae, all Mesembryanthemoideae, Anisostigmateae, *Tetragonia*, and *Gunniopsis*. Different *Mesembryanthemum* species are distributed among Ruschioideae. *Tetragonia* species group together. The smaller group is more heterogeneous and includes subgroup 3 represented by all species of *Aizoon* and all species of *Carpobrotus*, subgroup 2 with all species of Sesuvieae, and subgroup 1 with two species: *Acrosanthes anceps* and *Skiatophytum skiatophytoides*.

Two principal components explain 49.9% of total variance of species in their seed characters (**Figure 13B**). Ruschieae (black dots, squares, and diamonds) and Mesembryanthemoideae (red dots and squares) form a compact cluster on the left side of the space and correlate with a well-developed seed notch, the presence of waxes, puzzle-like borders of testa cells, and mostly D-shaped and pear-shaped seeds. Aizooideae (blue dots) and Sesuvioideae (green dots) are much more heterogeneous. Sesuvioideae correlates with the seed number, seed aril, and size and absence of waxes. Aizooideae ended up in several clusters where *Aizoanthemum* and *Aizoon* correlate with the seed color, presence of longitudinal ridges, stalactites in the outer wall of testa cells, and foveolate testa cells, whereas *Tetragonia* are linked with Sesuvioideae, and *Gunniopsis* with Ruschieae and Mesembryanthemoideae.

The results of univariate analysis of seed size and seed coat thickness in species with hygrochasy and species that lack hygrochasy are shown in **Figure 13C**. On average, the size of the seeds and the thickness of the seed coat are significantly greater in species that lack hygrochasy compared to those for which hygrochasy is typical. At the same time, the range of variations of seed size is high in those species with no hygrochasy, indicating that a number of species with small seeds lack hygrochasy. The range of variation of seed coat thickness is wide in both groups. This result suggests that there is a substantial number of species with a thick seed coat in which hygrochasy is present nevertheless. At the same time, there is a number of species with a thin seed coat in which hygrochasy is absent.

## 4 Discussion

### 4.1 Notable seed characteristics distinguishing the five subfamilies

#### 4.1.1 Sesuvioideae

a. Sesuvieae. All Sesuvieae have circumscissile capsules. The core genus *Sesuvium* is characterized by having 50 or even more seeds per capsule, except for *S. curassavicum* Sukhor., having only 6 to 10 seeds (Sukhorukov et al., 2021c). In three American species previously within *Cypselea* (*S. humifusum*, *S. rubriflorum*, and *S. mezianum*), the seeds are smaller and red and have a thinner testa. In the first two species, such a shift might be linked to their freshwater habitats on the Caribbean islands (Urban, 1929; Sukhorukov et al., 2021c). This is in contrast to other species of the genus growing under saline conditions: these species have a much thicker testa to protect the seed. The emergence of seeds with a thin testa in the Paraguayan endemic *S. mezianum* growing in saline substrates (Müller, 1909) is an exception.

Normally, two or four seeds are present in capsules of *Trianthema* and *Zaleya* (Jeffrey, 1960). In *Trianthema*, the seeds exhibit spatial heterospermy: uppermost ones have a horizontal embryo, whereas the lower seed is rotated by 90° to have a vertical embryo. Such positioning of the seeds in the capsule is unique within Aizoaceae and the entire Caryophyllales. Apart from the spatial heterospermy, the seeds do not show any difference in shape, ultrasculpture, and thickness of the testa. Some species have four to only one seed, possibly due to abortion of some ovules. Such variable numbers were discovered in *Trianthema salsoloides* Fenzl and *T. transvaalense* Schinz (Hartmann et al., 2011). We also noticed a deviation from the usual two-seeded fruits in *T. crystallinum* and *T. hereroense*, which can possess either one or two seeds per fruit. These changes in the number of seeds in some *Trianthema* members do not affect any fruit traits and dispersal modes.

b. Anisostigmateae. This tribe, represented by *Tribulocarpus* (three spp.) and the monotypic *Anisostigma*, is characterized by two different diaspore types (**Figures 1A, B**). These diaspores are indehiscent and constitute a single unit of dispersal.

The first type is winged nut-like diaspores (*Tribulocarpus retusus* (Thulin) Thulin & Liede and *Anisostigma schenckii* Schinz). This shift to winged diaspores in these two species from the deserts of Somalia and Namibia, respectively, is associated with anemochory (Thulin et al., 2012). *Tribulocarpus retusus* has two seeds per fruit (Thulin, 1993, as *Tetragonia retusa* Thulin), whereas each fruit of *A. schenckii* has a single seed. By contrast, (Schinz, 1894, as *Tetragonia schenckii*) stated that there are 2–3 ovular hollows in the fruit, but only one ovule develops into a seed. We also observed only a single fully developed seed with a thin (15–20 μm) smooth-to-slightly-mamillate coat tightly adhering to the lowermost pericarp layer. The embryo points upwards and pushes through the softer upper pericarp portion after germination. We noted only a small amount of the perisperm in contrast to previous observations (Pax, 1889; Schinz, 1894; Hartmann, 1993), and this trait is unique within Aizoaceae (see also **Supplementary File 2**).

The second type is the synaptospermic spiny burrs found in *Tribulocarpus dimorphanthus* and *T. somalensis*: species also confined to deserts of Namibia and Somalia, respectively (Thulin et al., 2012; Sukhorukov et al., 2019a). The exact number of seeds and their anatomy in these taxa remain unknown because it is very difficult to extract the seeds from indurated burr covers. This is probably the reason why many authors (e.g., Engler in Schellenberg, 1912, as *Tetragonia somalensis*; Thulin et al., 2012; Thiede, 2017) have not paid attention to internal morphology of the burrs. Moore (1921) reported a two-locular ovary in *Tribulocarpus dimorphanthus*, whereas Sukhorukov et al. (2019a) noted several ripe seeds in a burr. By electron tomography, we detected three to six seed hollows in the burrs of both species (**Figures 2O, P**). Some hollows are empty and three to four ripe seeds are usually present in each burr.

The burrs of *T. dimorphanthus* and *T. somalensis*, just as winged two-seeded diaspores of *T*. *retusus*, can be characterized as synaptospermic. Despite the relatively thin seed coat in all species of Anisostigmateae, the other hard diaspore covers perform at least two functions: (1) epizoochorous dispersal in the burrs and anemochorous dispersal in the winged diaspores (Thulin et al., 2012) and (2) protection of the seeds against unfavorable climatic conditions or predation as in other plants having similar synaptospermic diaspores (Quinn, 1987; Gutterman, 1994). The spines on the burrs in *Tribulocarpus* may also anchor the diaspores in the substrate. We did not examine the fruits of *Tribulocarpus retusus*, but there are several characteristic states that unite it with other Anisostigmateae (Thulin, 1993, as *Tetragonia retusa*; Thulin et al., 2012).

Diaspores are dispersed differently and have different numbers of seeds if we compare Sesuvieae and Anisostigmateae. In agreement with this observation, Anisostigmateae are distant from Sesuvieae in many features of their seeds. These include the absence of seed aril (state 3:0), seed shape (5:0), larger size (6:3), smooth or rugose surface ultrasculpture (9:0), and a thinner seed coat (17:0).

#### 4.1.2 Aizooideae

There are two major lineages in Aizooideae (Klak et al., 2017a); one includes *Aizoon* (including *Galenia* L. and *Plinthus* Fenzl) + *Aizoanthemum* and is a sister lineage to a clade including three other genera (*Aizoanthemopsis*, *Gunniopsis*, and *Tetragonia*). Seeds of three genera (closely related *Aizoon* and *Aizoanthemum* and phylogenetically distant *Aizoanthemopsis*) share many states of characters (**Supplementary File 2**). They can easily be distinguished from the rest of Aizoaceae by black, reniform, and longitudinally ridged seeds. Many species in these genera also share the presence of pits (foveolae) on the seed surface. Furthermore, all of them possess approximately thick seed coats with stalactites in the testal layer and a bent embryo. The merger of *Plinthus* and *Galenia* with *Aizoon* (Klak et al., 2017a) is supported by the seed characters because neither of these two former genera has unique features of their seeds. *Aizoon fruticosum* (formerly *Galenia fruticosa*) and *A. sericeum* (Pax) Klak (formerly *Plinthus sericeus* Pax) possess a single seed in the nut and variable (1–2) number of seeds per capsule, respectively, but such deviations are not seen in any other members of *Aizoon*.

Four *Gunniopsis* species studied here as well as other *Gunniopsis* members investigated earlier (Chinnock, 1983) differ drastically from *Aizoon* and *Aizoanthemum* (and also partially from *Aizoanthemopsis*). Nevertheless, both *Aizoanthemopsis* and *Gunniopsis* have waxes on seeds, whereas other genera are devoid of them. Some characters of *Gunniopsis* resemble those of Mesembryanthemoideae and Ruschioideae (puzzle-like borders of testa cells, and D-shaped outlines of some seeds) and are absent in all other Aizooideae. Such traits are remarkable homoplasy in Aizooideae, Mesembryanthemoideae, and Ruschioideae. Many distinct states of characters can be explained by geographical isolation of exclusively Australian *Gunniopsis* from South African genera *Aizoon*, *Aizoanthemum*, and *Aizoanthemopsis*. Within *Gunniopsis*, seeds are also quite different, especially between the annual species (*G. calva* Chinnock and *G. papillata* Chinnock) and shrubby species (*G. calcarea* Chinnock and *G. quadrifida* (F.Muell.) Pax). We note here that the seed coat of the annuals *G. calva* and *G. papillata* is thinner than that of the shrubs *G. calcarea* and *G. quadrifida*. In *G. papillata*, prismatic monocrystals were registered, which have not been observed in any other species of Aizoaceae. Our own observations (**Supplementary File 2**) and those of Chinnock (1983) suggest that the seeds in *Gunniopsis* are diverse and need further research from taxonomic and ecological points of view.

*Tetragonia* has ~50 species that occur in the Southern Hemisphere (Hartmann, 2017; Klak et al., 2017a). Many species are coastal, and their diaspores can stay afloat for a long time, allowing for dispersal by water currents (Bogle, 1970). *Tetragonia* has indehiscent multi- to one-seeded (synaptospermic) fruits, whereas all other genera in the subfamily have dehiscent capsules, with one-seeded fruits being rare exceptions (Hartmann, 2017). The number of seeds in each examined species is as follows: 3 or 4 seeds in *T. echinata* Aiton; two seeds and two empty hollows in *T. spicata* L.f. and *T. tetragonoides*, and one seed in *T. saligna* Fenzl. Thus, *Tetragonia* produces both synaptospermic and nut-like fruits. The seeds have an unusual pear-like shape. Although the hardened pericarp protects the entire diaspore, the seed coat in all species under study is transparent, very thin (5–8 μm), and without stalactites in the testal layer.

#### 4.1.3 Acrosanthoideae

This subfamily includes only *Acrosanthes* (six spp.) and is characterized by fruits with a parchment-like pericarp that is not found in any other Aizoaceae (Klak et al., 2017a). Basal stipitate ovules are an additional diagnostic feature for *Acrosanthes* (Klak et al., 2017a). The ovary is two-loculate with one seed in each locule, but sometimes only one seed in the capsule develops fully (Adamson, 1959). In several characteristics, *Acrosanthes* (*A. anceps* Sond. and *A*. *humifusa* Sond.) resembles many *Trianthema* members (Sesuvioideae), especially in depressed-roundish, large, ridged seeds with a colliculate surface and annular embryo. Perhaps for this reason, the species *A. anceps* and *A. humifusa* were described as *Trianthema* (Thunberg, 1794). The thick seed coat of pyrophytic *A. anceps* may provide protection from high temperatures during fires.

The foveolate surface of *Acrosanthes* is also typical for some Aizooideae. Other than that, there is little morphological resemblance between the seeds of *Acrosanthes* and those of Aizooideae (**Supplementary File 2**), where it was previously placed (e.g., Fenzl, 1840; Dietrich, 1843; Hartmann, 2017).

#### 4.1.4 Mesembryanthemoideae

All of approximately 100 species of *Mesembryanthemum* (Klak and Bruyns, 2012), with one exception (*M. nucifer*), have multi-seeded capsules. *Mesembryanthemum nucifer* has one- to two-seeded capsules and also deviates in several other traits of the seeds (shape, the absence of a notch and waxes, the smooth surface, and a straight or bent embryo). Seeds of the other species are all quite similar in their shape, the presence of a notch, puzzle-like borders of testa cells, waxes, and bent embryos. Some representatives have cristate (*M. lignescens* (L.Bolus) Klak and *M. tetragonum* Thunb.) or finger-like (*M. rabiei* (L.Bolus) Klak) shapes of the testa cells. The color varies from yellowish-brown to dark-brown and this characteristic seems to be suitable for the subdivision of *Mesembryanthemum* s.l. into major groups (Klak et al., 2007). In some species, however (e.g., *M. clandestinum* Haw., *M. crystallinum* L., and *M. nodiflorum* L.), the color of the seed coat can change from brown or dark brown in the area of the embryo to yellowish-brown in the part of the seed containing the perisperm. Waxes are present on the surface in many species (see also Ehler and Barthlott, 1978).

#### 4.1.5 Ruschioideae

a. Apatesieae. This tribe contains only 11 species (Klak et al., 2015; **Table 1**), which share many vegetative and reproductive features (Ihlenfeldt and Gerbaulet, 1990; Chesselet et al., 2002). They have relatively large, roundish or reniform, dark (brown to black) seeds (Ihlenfeldt and Gerbaulet, 1990). We investigated all genera (except *Saphesia* N.E.Br.), and all of them have seeds with annular embryos unlike those in any other Ruschioideae. The surface is smooth or mamillate, and larger outgrowths were not observed. A keel is present in many representatives (such as *Carpanthea*, *Conicosia*, and *Hymenogyne glabra*).

b. Dorotheantheae. The 14 species of this small tribe (**Table 1**) have seeds that are D-shaped or rarely depressed-roundish and are similar to group 2 (see below) of Ruschieae. Puzzle-like cell borders are found only in *Cleretum pinnatifidum* (L.f.) N.E.Br.

c. Drosanthemeae. This tribe contains two genera: *Drosanthemum* (ca. 120 spp.) and monotypic *Lemonanthemum* Klak (Klak et al., 2020). Species of *Drosanthemum* studied here are quite different in seed shape, color, dimensions, the presence of waxes, and embryo shape, but all species have seeds without a keel or longitudinal ridges and with puzzle-like cell borders. The thickness of the seed coat varies widely: the thinnest (5–8 μm) is seen in *D. dejagerae* L.Bolus, and the thickest (55–75 μm) in *D. asperulum* Schwantes.

d. Ruschieae. Its genera can be placed in three informal groups on the basis of their seeds:

(1) Those with pear-shaped seeds and a mostly very thin seed coat (*Antimima* N.E.Br., *Argyroderma* N.E.Br., *Cheiridopsis* N.E.Br., *Conophytum* N.E.Br., *Deilanthe* N.E.Br., *Delosperma* N.E.Br., *Disphyma* N.E.Br., *Dracophilus* Dinter & Schwantes, *Eberlanzia* Schwantes, *Ebracteola* Dinter & Schwantes, *Gibbaeum* Haw., *Glottiphyllum* Haw., *Hallianthus* H.E.K.Hartmann, *Lapidaria* Dinter & Schwantes, *Leipoldtia* L.Bolus, *Lithops* N.E.Br., *Mitrophyllum* Schwantes, *Namibia* Dinter & Schwantes, *Peersia* L.Bolus, *Vanheerdea* L.Bolus, and *Vanzijlia* L.Bolus). Almost all these genera have straight to r-shaped embryos.

(2) Those with D-shaped, ovate, or roundish seeds (*Circandra* N.E.Br., *Cylindrophyllum* Schwantes, *Erepsia* N.E.Br., *Esterhuysenia* L.Bolus, *Khadia* N.E.Br., *Lampranthus* N.E.Br., *Malephora* N.E.Br., *Mestoklema* N.E.Br., *Namaquanthus* L.Bolus, *Oscularia* Schwantes, *Pleiospilos* N.E.Br., *Ruschia* Schwantes, *Ruschiella* Klak, *Scopelogena* L.Bolus, *Smicrostigma* N.E.Br., *Stayneria* L.Bolus, and *Stoeberia* Dinter & Schwantes). Most of them have a thicker seed coat (states 17:1 and 17:2), but a thin coat (17:0) is also not rare. The embryo is r-shaped or bent.

(3) Those with ovate smooth seeds with stalactites in the testal layer (*Carpobrotus*). The alterations of many characteristics in this genus relate to specific features of its fruit, e.g., indehiscence and endozoochory (D’Antonio, 1990; Campoy et al., 2018). Although the seeds are smooth (very unusual for Ruschieae), puzzle-like cell borders are present in *C. edulis* (**Figure 6J**).

### 4.2 Comparison of seeds between Aizoaceae and other Caryophyllales

The seeds of many Caryophyllales are relatively well-studied morphologically and anatomically, thus enabling a comparison with the seeds of Aizoaceae. A funicular aril, which is found among Aizoaceae only in the tribe Sesuvieae (Sesuvioideae), was not mentioned in an earlier treatment of expanded Aizoaceae (Pax, 1889), but was discovered by Müller (1909) and subsequently confirmed by embryological research (e.g., Bhargava, 1935; Raghavan and Srinivasan, 1940). In contrast to other Caryophyllales having a funicular appendage (for more information, see Sukhorukov et al., 2018b), Sesuvieae members possess a parenchymatous one- or two-layered aril usually entirely covering the seed. Reports of the aril covering only up to half of the seed (Hassan et al., 2005) are an exception rather than a rule (Sukhorukov et al., 2018a). Seeds that are only partially covered with an aril have only occasionally been observed in *Sesuvium portulacastrum* and the related species *S. curassavicum* (Sukhorukov et al., 2021c). Claims that the aril is absent in *Trianthema portulacastrum* and *T. rhynchocalyptrum* F.Muell. (Sesuvioideae: Hassan et al., 2005; Fahmy et al., 2019) are erroneous, and the porous surface demonstrated by Fahmy et al. (2019) and Fakhr et al. (2022) is indeed the surface of the aril. The function of the aril in Sesuvieae remains unclear.

The aril (if present) in all other Caryophyllales is usually a small appendage located near the hilum (Gvinianidze and Fedotova, 1991; Minuto et al., 2006; Sukhorukov et al., 2015; Sukhorukov et al., 2018b; Sukhorukov et al., 2021b). This small appendage may be related to myrmecochory (e.g., Nilsson, 1971). Nonetheless, many Cactaceae have an aril that may cover the seed entirely thereby protecting the seed by its hardness (Barthlott and Voit, 1979; Bregman, 1988; Orozco-Segovia et al., 2007). In other Caryophyllales, a funicular aril is found in taxa with two- or multi-seeded capsules, whereas one-seeded fruits and fruits with one-seeded mericarps do not have it.

The phenomenon of fruits breaking into mericarps is rare in Caryophyllales but is characteristic of monotypic families Limeaceae and Gisekiaceae as well as for Phytolaccaceae s.str.

Seed colors seen in Aizoaceae are common within Caryophyllales, except for *Drosanthemum dejagerae*’s bicolored seeds, which are unique in Caryophyllales.

Depressed-roundish and kidney-shaped (including drop-like) seeds are widespread in Aizoaceae and are prevalent in many other Caryophyllales families, such as Caryophyllaceae (Gvinianidze and Fedotova, 1991), Portulacaceae (Ocampo, 2013), Amaranthaceae s.l. (Sukhorukov, 2014), Lophiocarpaceae (Sukhorukov and Kushunina, 2015), and some Petiveriaceae and Molluginaceae (Sukhorukov et al., 2018b). D-shaped seeds have been documented in some Molluginaceae (Sukhorukov and Kushunina, 2017). Elongated seeds (states 5:0 and 5:4) are not so common in Caryophyllales and are found in certain tribes of Caryophyllaceae (Gvinianidze and Fedotova, 1991) and Amaranthaceae s.l. (Sukhorukov, 2014). Pear-shaped seeds are known in *Anacampseros* L. of Anacampserotaceae (Nyananyo, 1988) and in Cactaceae (Parfitt and Gibson, 2003).

Keeled seeds are very rare in Aizoaceae and are more frequent in other groups of Caryophyllales, e.g., in Amaranthaceae s.l., especially in Chenopodioideae (Sukhorukov and Zhang, 2013) and the ‘Amaranthoids’ clade (Bojnanský and Fargašová, 2007), in some Cactaceae (Arroyo-Cosultchi et al., 2006), Caryophyllaceae (Gvinianidze and Fedotova, 1991), Molluginaceae (Sukhorukov et al., 2018b), Montiaceae (Nilsson, 1971; Elvebakk et al., 2015), and *Hilleria* Vell., Petiveriaceae (Sukhorukov et al., 2015).

Certain families in Caryophyllales also lack a prominent seed notch, e.g., Chenopodiaceae (Sukhorukov and Zhang, 2013; Sukhorukov, 2014), Montiaceae (*Calandrinia* Kunth: Elvebakk et al., 2015), Molluginaceae, and Kewaceae (Sukhorukov and Kushunina, 2017; Sukhorukov et al., 2018b). A short notch is present in some *Portulaca* L. (Portulacaceae: Ocampo, 2013). In general, this trait has not been searched for exhaustively across Caryophyllales.

The ridged seed surface of *Aizoon*, *Aizoanthemum*, and *Aizoanthemopsis* resembles that of some American *Mollugo* L. members (Molluginaceae) (Sukhorukov and Kushunina, 2017). This trait seems to be rare in Caryophyllales, where most species have smooth or mamillate surfaces (e.g., Gvinianidze and Fedotova, 1991; Hassan et al., 2005; Ocampo, 2013; Sukhorukov et al., 2015; Sukhorukov et al., 2018b). In the Chenopodiaceae–Amaranthaceae alliance, however, smooth and rugose sculpturing has evolved (Sukhorukov and Zhang, 2013; Sukhorukov, 2014). Finger-like outgrowths of the seed coat are also present in some *Spergula* L. (Caryophyllaceae: Gvinianidze and Fedotova, 1991), *Anacampseros* (Anacampserotaceae: Nyananyo, 1988), and *Portulaca*, Portulacaceae (Ocampo, 2013).

Netolitzky (1926) reported that puzzle-like borders of testa cells in *Mesembryanthemum* resemble those of Caryophyllaceae. In addition, they are found in *Portulaca* (Portulacaceae: Matthews and Levins, 1986; Danin et al., 2008) and in *Calandrinia* s.l. [*Rumicastrum* s.str.] (Montiaceae: Obbens, 2006) as well as in many Caryophyllaceae (Barthlott, 1981; Wofford, 1981; Abdel-Maksoud and Fawzi, 2016; Pirani et al., 2019).

Foveolate pits on the seed surface appear to be rare in Caryophyllales. They are common only in (almost all) *Paramollugo* Thulin and *Trigastrotheca molluginea* F.Muell. (Molluginaceae: Hassan et al., 2005, as *Mollugo nudicaulis* Lam.; Sukhorukov and Kushunina, 2017, as *Mollugo* spp.) as well as in some *Chenopodium* members (e.g., *C. bryoniifolium* Bunge) and *Chenopodiastrum hybridum* (L.) S.Fuentes, Uotila & Borsch (Uotila, 2001).

In core Caryophyllales, waxes on the seed surface are present in *Simmondsia* (Simmondsiaceae: Gülz and Hangst, 1983), some Cactaceae (Barthlott and Voit, 1979), Montiaceae (Obbens, 2006), Caryophyllaceae (Barthlott, 1981; Minuto et al., 2006; Romanova and Kravtsova, 2019), rarely in *Portulaca* (Portulacaceae: Danin et al., 2008; Santos et al., 2015), and in Molluginaceae (Sukhorukov and Kushunina, 2017; Sukhorukov et al., 2018b). In the species researched to date, waxes are found on seeds in multi-seeded fruits. Nonetheless, in some genera with multi-seeded fruits, i.e., *Talinum* Adans. (Talinaceae: Veselova et al., 2012; Moises Mendoza and Wood, 2013) and *Corbichonia* Scop. (Lophiocarpaceae: Sukhorukov and Kushunina, 2015), waxes are absent. Seeds from nut-like or synaptospermic fruits also lack waxes on their surface (Aizoaceae: *Mesembryanthemum nucifer*, *Anisostigma schenckii*, and *Tribulocarpus* spp.; Chenopodiaceae-Amaranthaceae: Sukhorukov et al., 2019b).

The perisperm, which is a special type of seed nutritive tissue, is regarded as an important characteristic in Caryophyllales (Rocen, 1927; Eckardt, 1976; Takhtajan, 1991). Its presence or absence usually characterizes an entire family, but in the large Chenopodiaceae clade, some subfamilies (Suaedoideae and Salsoloideae) are devoid of it in mature seeds, although it is present in other subfamilies (Betoideae, Chenopodioideae, and Corispermoideae). In Aizoaceae, character 15 is not applicable to systematics of large intrafamilial groups but can rather be considered an additional trait characterizing a shift to one-seeded fruits.

The embryo shape serves as an additional distinguishing feature of major groups within Aizoaceae. This is not surprising because other large and diverse families within Caryophyllales, such as Cactaceae, Chenopodiaceae-Amaranthaceae, and Caryophyllaceae, also differ in embryo shape (Martin, 1946; Sukhorukov et al., 2015; Baskin and Baskin, 2019; Vandelook et al., 2021).

Thickness of the seed coat is highly variable both in Aizoaceae and in the entire Caryophyllales (see Sukhorukov and Zhang, 2013; Sukhorukov et al., 2015; Sukhorukov et al., 2018b). As in other groups (Sukhorukov et al., 2018b), brighter (yellow and brown) seeds have a thinner testa.

In contrast to Aizoaceae, darker stalactites in cell walls of testa cells occur frequently among Amaranthaceae s.l. members, especially in Chenopodioideae and the ‘Amaranthoids’ clade (Sukhorukov, 2014), Lophiocarpaceae, Montiaceae, Nyctaginaceae-Boldoeae (Netolitzky, 1926; Wunderlich, 1967; Sukhorukov et al., 2015), Talinaceae (Veselova et al., 2012), some Cactaceae (Vyshenskaya, 1991) and Molluginaceae (Sukhorukov et al., 2018b), and in all Kewaceae (Sukhorukov et al., 2018b).

### 4.3 Evolution of hygrochasy coupled to dispersal by rain

Hygrochasy is the process of opening of fruits (capsules) upon moistening by rain, with a possible release of some seeds, followed by closure of the capsules afterwards as they dry out (Parolin, 2001). This is known to happen in some plants in temperate climates (Steinbrinck, 1883; Nakanishi, 2002) but is especially advantageous for the dispersal of seeds in arid environments. In most cases, hygrochasy in drought-tolerant plants denotes ombrohydrochory: the capsules open when rain drops fall on them (Berger, 1908; Parolin, 2006). Among Caryophyllales, hygrochasy is found in all *Glinus* (Molluginaceae: Sukhorukov et al., 2021b), some *Sagina* L. (Caryophyllaceae: Gibbs, 1950; Nakanishi, 2002), and in many Aizoaceae, predominantly of Ruschioideae and Mesembryanthemoideae (e.g., Schmid, 1925; Garside and Lockyer, 1930; Ihlenfeldt and Gerbaulet, 1990; Croizat, 1993; Parolin, 2001; Parolin, 2006; Kurzweil and Burgoyne, 2009). It is also present in some Aizooideae: in all *Gunniopsis* (Chinnock, 1983) and in all *Aizoanthemum* (Klak et al., 2017a).

The hygroscopic tissue consists of expanding keels and/or sheets inside valves and is responsible for the rapid opening of the valves (Garside and Lockyer, 1930; Schwantes, 1952; Chinnock, 1983; Kurzweil and Burgoyne, 2009; Harrington et al., 2011). Klak et al. (2017b) suggested that the expanding keels in the capsules arose after the split of Sesuvioideae from the remainder of Aizoaceae and were subsequently reduced or lost repeatedly in the family. In Aizooideae, where fruits open only slightly, the expanding keels are strongly shortened or absent (Bittrich, 1990). Despite reports of hygrochasy in *Aizoon canariense* L. (Ellner and Shmida, 1981; Brown and Mies, 2012; Hartmann, 2017), we cannot confirm the rapid opening after moistening in capsules of any species of *Aizoon*. The capsules of *Aizoanthemum*, *Aizoanthemopsis*, and *Gunniopsis* (Aizooideae) open rapidly with moisture. We confirmed rapid hygrochasy (within one to several minutes) in all the Ruschioideae and Mesembryanthemoideae species where it has been reported (Garside and Lockyer, 1930; Ihlenfeldt and Gerbaulet, 1990; Kurzweil and Burgoyne, 2009; see also **Supplementary File 2**), except in one-seeded *Mesembryanthemum nucifer* (Gerbaulet in Hartmann, 2017, as *Brownanthus nucifer* (Ihlenf. & Bittrich) S.M.Pierce & Gerbaulet), which has non-hygrochastic fruits. Reduction or loss of the expanding tissue has occasionally occurred also in Ruschioideae (e.g., most species in Apatesieae, *Ruschianthemum gigas* (Dinter) Friedrich, and *Carpobrotus* spp.). Although the expanding tissue has been lost several times independently in Ruschioideae and once in Mesembryanthemoideae (in *Mesembryanthemum nucifer*), no reversals to hygrochasy have been documented, suggesting that its loss is irreversible (Figure 3 in Klak et al., 2017b). The shift to xerochastic fruits is coupled with a reduction of seed number to one or two (*Mesembryanthemum nucifer*), a change of the dispersal mode to endozoochory (*Carpobrotus*: D’Antonio, 1990; Campoy et al., 2018), or drastic changes in the seeds as in Apatesieae.

### 4.4 Evolution of one-seeded or compound fruits associated with switching to anemochory and epizoochory

Multi-seeded fruits are seen in many Caryophyllales, especially in Cactaceae, Caryophyllaceae, Molluginaceae, Montiaceae, Phytolaccaceae, Talinaceae, and some other smaller families. The emergence of one-seeded fruits in the order is mostly connected with a reduction in the number of seeds, but the reverse situation is known in some Amaranthaceae s.str. (Celosieae) and seemingly in Caryophyllaceae (Sukhorukov et al., 2015). In Aizoaceae, the drastic reduction of the number of seeds toward nuts is quite rare (**Figure 11**) and is known in a few unrelated genera across the family: *Anisostigma* (Sesuvioideae-Anisostigmateae), *Trianthema* (Sesuvioideae-Sesuvieae), *Aizoon fruticosum*, *Tetragonia saligna* (Aizooideae), and *Mesembryanthemum nucifer* (Mesembryanthemoideae). On the other hand, synaptospermic fruits (indehiscent fruits with two or several seeds: as in *Tribulocarpus*, Anisostigmateae; and *Tetragonia*, Aizooideae) have also evolved in Aizoaceae. The emergence of one-seeded or synaptospermic fruits is often linked with completely different types of dispersal, especially anemochory in desertic taxa *Tribulocarpus retusus* and *Anisostigma* (Thulin et al., 2012; Klak et al., 2017a; Sukhorukov et al., 2021a), epizoochory in two other *Tribulocarpus* members (Thulin et al., 2012; Sukhorukov et al., 2019a), hydrochory in *Tetragonia* (Taylor, 1994), and autochory (probably) in *Mesembryanthemum nucifer*. It is coupled with further alterations of structure in the perianth, pericarp, and seeds. Reversal to multi-seeded fruits is not known in Aizoaceae.

The unique mericarpic fruits where the parts (mericarps) are marginally elongated into papery wings occur in *Hymenogyne*. In *Skiatophytum skiatophytoides*, the fruits also consist of mericarps but are woody. *Conicosia* has schizocarps, which also break up into somewhat winged mericarps after drying. This feature is found only in Apatesieae and in *Ruschianthemum gigas* (Ruschieae), all in Ruschioideae.

### 4.5 Seed coat thickness as an adaptation to environmental conditions

#### 4.5.1 A thin seed coat as a means for fast germination in arid environments

Across Aizoaceae, there is remarkable variation of the thickness of the seed coat (see **Supplementary File 7**). A very thin seed coat up to 20 μm is mostly known in Mesembryanthemoideae and Ruschioideae (except for Apatesieae). This trait may allow for higher germination rates and is found in many Caryophyllales (Baar, 1913; Williams and Harper, 1965; Maiti et al., 1994; Parsons, 2012; Sukhorukov and Zhang, 2013; Parsons et al., 2014) and in other orders (Parsons, 2012; Steinbrecher and Leubner-Metzger, 2017, with references therein). Germination rates of seeds can also depend on their position in the capsule, as in annual *Mesembryanthemum nodiflorum*, where the seeds located in the uppermost part of the capsule have much better germination rates than those coming from lower parts of the fruit (Gutterman, 1980). Different germination rates of the seeds between central and peripheral capsules were also found in perennial *Glottiphyllum linguiforme* N.E.Br. (Gutterman, 2012). Although the seeds within the capsule (Gutterman, 1980) or within one individual (Gutterman, 2012) have no morphological differences, they may have dissimilar levels of physiological dormancy (Baskin and Baskin, 2004; Visscher et al., 2018; Visscher et al., 2022). On the other hand, slightly delayed germination in Mesembryanthemoideae and Ruschioideae is connected with insufficient amounts of water during the beginning of the rainy season (Bittrich and Ihlenfeldt, 1984), although many of their members have thin seed coat.

Data about seed germination are insufficient for many Aizoaceae. Rapid germination of at least some seeds within one year after dispersal is confirmed in *Mesembryanthemum nodiflorum* (Gutterman, 1980) and *M. crystallinum* (Adams et al., 1998). Several species of Ruschioideae (*Drosanthemum asperulum*, *Ruschia spinosa*, *R. tenella*) possess seeds with different germination rates (Esler and Cowling, 1995; Schmiedel et al., 2021). The seeds of *Ruschia spinosa* and *Drosanthemum asperulum* germinate fast, whereas *R. tenella* seeds germinate gradually. Delayed germination has been reported in *Sesuvium portulacastrum* (Martínez et al., 1992; Lonard and Judd, 1997), *Trianthema portulacastrum* (Tanveer et al., 2013), *Zaleya pentandra* (Munawar et al., 2015), and all of them have a thick seed coat.

#### 4.5.2 Thick seed coats protect from fires

A different case is the combination of hygrochastic fruits and black seeds with a medium to thick coat, as in certain species of pyrophytic habitats such as *fynbos* and *renosterveld*. Taxa endemic to such habitats are members of the tribes Dorotheantheae and Ruschieae: several members of *Cleretum, Circandra*, *Stayneria*, *Drosanthemum asperulum*, *Esterhuysenia drepanophylla* (Schltr. & A.Berger) H.E.K.Hartmann, and *Erepsia* (**Supplementary File 2**). Besides, *Acrosanthes* and the members of Apatesieae that occur in *fynbos* (albeit without hygrochastic capsules) are characterized by seeds with a thick testa. Exceptions are two species of *Lampranthus* (*L. reptans* (Aiton) N.E.Br. and *L. explanatus* (L.Bolus) N.E.Br.) and *Smicrostigma*, which have thin seed coats. It is possible that survival after a fire is low for these species and that populations from burnt areas need to be replenished from regions not damaged by fires.

#### 4.5.3 The relation between hygrochasy and seed coat thickness

The hygrochastic opening of capsules is a necessary but insufficient step for the establishment of offspring (Friedman et al., 1978) and should correlate with such seed characteristics as seed coat thickness and eventually dormancy release. Together with temperature, light, salinity, depth of sowing, physiological dormancy, and other parameters, the anatomy of the seed coat has proven to be an important factor accelerating or delaying germination (Parsons, 2012; Smýkal et al., 2014). The relation between seed coat thickness and germination can be evaluated as different levels of physical dormancy (Baskin and Baskin, 2004). *Anabasis aretioides* Moq. & Coss. (Chenopodiaceae/Amaranthaceae) from the Sahara Desert serves as a good example of this correlation between fruit and seed structure and germination capability. The fruit and seed covers of *A. aretioides* are very thin, reaching only several micrometers (Sukhorukov, 2008, as *Fredolia aretioides* (Moq. & Coss.) Ulbr.) and enable rapid unfolding of the embryo within 10 min of saturation with water (Grenot, 1974). Fast germination is also known in various Chenopodiaceae-Salsoloideae members and other often unrelated families from dry habitats (Parsons, 2012).

Our own data show for the first time that there is also a correlation between hygrochasy and seed structure. Our multivariate analysis suggests that hygrochasy in Aizoaceae correlates with a smaller seed size and thinner seed coat. Almost all Sesuvioideae and Aizooideae with a thick seed coat do not have hygrochastic fruits, whereas many species of Mesembryanthemoideae and Ruschioideae – with diverse life forms and from different habitats – combine hygrochasy and a thin seed coat (**Supplementary File 2**). Both characteristics, namely a rapid response of capsules to moisture and a thin seed coat, facilitate rapid seed germination, which has been reported e.g. in *Mesembryanthemum*, *Glottiphyllum linguiforme, Ruschia spinosa* (L.) Dehn, and *Drosanthemum asperulum* (Esler and Cowling, 1995; Gutterman, 2012). The simultaneous presence of hygrochasy and a relatively thick seed coat with stalactites (in *Aizoanthemum*, *Aizoanthemopsis* [Aizooideae], and some Ruschioideae) is still unexplained but may present an adaptation to saline and fire-prone conditions in their habitats.

In other Caryophyllales, the correlation between hygrochasy and seed structure is evident in *Sagina* species (Caryophyllaceae), which have small seeds with a thin coat (Gvinianidze and Fedotova, 1991). In *Glinus* (Molluginaceae), the seeds do not exceed 1 mm, but the drought-adapted species (*G. lotoides* L., *G. orygioides* F.Muell., and *G. setiflorus* Forssk.) have a medium or thick seed coat, whereas mesophytic species (e.g., *G. oppositifolius* (L.) Aug.DC. and *G. radiatus* (Ruiz & Pav.) Rohrb.) are characterized by a thin seed coat (Sukhorukov et al., 2021b).

## 5 Conclusions

This work revealed a high diversity of characteristics of seeds across Aizoaceae. There are almost no characteristics specific to each subfamily, and the characteristics are highly convergent across subfamilies. Reduction to one-seeded or synaptospermic fruits is accompanied by drastic changes to the structure of these seeds and their mode of dispersal, when compared to close relatives having multi-seeded capsules. In many species, hygrochasy is combined with a thin seed coat, thereby playing a crucial role in rapid germination of these seeds under semiarid conditions.

## Data availability statement

The datasets presented in this study can be found in online repositories. The names of the repository/repositories and accession number(s) can be found in the article/**Supplementary Material**.

## Author contributions

Conceived and designed the manuscript, AS and CK. Field sampling, AS and CK. Data analysis, AS, CK, and YM. Writing and figures, AS, MK, MN, YM, and CK. Final proofreading and editing, AS, MK, and CK. All authors contributed to the article and approved the submitted version.

## Supplementary material

Supplementary File 1 Material used for the carpological examination.

Supplementary File 2 List of species and coding of the carpological characters.

Supplementary File 3 List of species with voucher information and GenBank accession numbers. All vouchers are kept at BOL, if not indicated otherwise.

Supplementary File 4 SEM photographs or tomograms of the seeds of investigated Aizoaceae species.

Supplementary File 5 SEM micrographs of seeds of selected Ruschioideae species. **(A, B)** *Disphyma crassifolium*, **(C, D)** *Eberlanzia gravida*, **(E, F)** *Erepsia dunensis*, **(G, H)** *Esterhuysenia drepanophylla*, **(I, J)** *Glottiphyllum linguiforme*, **(K, L)** *Hallianthus planus*, (M, N) *Khadia acutipetala*, **(O, P)** *Lampranthus bicolor*, **(Q, R)** *Namaquanthus vanheerdei*, and **(S, T)** *Ruschia dejagerae*. Magnification: A, C, E, G, I, K, M, O, Q, and S: 70×; B, D, F, H, J, L, N, P, R, and T: 300×.

Supplementary File 6 Seed coat cross-sections of selected Ruschioideae species. **(A)** *Acrodon parvifolius*, **(B)** *Antimima ventricosa*, **(C)** *Astridia longifolia*, **(D)** *Braunsia geminata*, **(E)** *Carpobrotus acinaciformis*, **(F)** *Cheiridopsis peculiaris*, **(G)** *Circandra serrata*, **(H)** *Cylindrophyllum hallii*. Scale bar – 100 µm.

Supplementary File 7 Seed coat cross-sections of investigated Aizoaceae species.

Supplementary File 8 Maximum likelihood tree based on four chloroplast markers showing subfamiliar relationships in the Aizoaceae. Numbers indicate bootstrap node support.
