# Supplementary material for: Evolution of seed characters and of dispersal modes in Aizoaceae
Source: Front Plant Sci. 2023 Mar 22;14:1140069. doi: 10.3389/fpls.2023.1140069 (PMC10073613; doi:10.3389/fpls.2023.1140069)
Supplement: Supplementary File 1 — Material used for the carpological examination. [file DataSheet_1.zip › Data Sheet/Supplementary file 4.docx]

Supplementary Material

Evolution of seed characters and dispersal modes in Aizoaceae

**Alexander P. Sukhorukov*, Maya V. Nilova, Yuri Mazei, Maria Kushunina, Cornelia Klak**

*** Correspondence:** Corresponding Author: [suchor@mail.ru](mailto:suchor@mail.ru)

# Supplementary file 4. SEM photographs or tomograms of the seeds of investigated Aizoaceae species.

**

**

**Figure S1.** Tomogram of *Anisostigma* *schenckii* fruit.





**Figure S2.** Seed of *Sesuvium* *humifusum*. (A) 70× magnification, (B) 300× magnification.





**Figure S3.** Seed of *Sesuvium* *portulacastrum*. (A) 70× magnification, (B) 300× magnification.





**Figure S4.** Seed of *Sesuvium* *verrucosum*. (A) 70× magnification, (B) 300× magnification.





**Figure S5.** Seed of *Sesuvium* *rubriflorum*. (A) 70× magnification, (B) 300× magnification.





**Figure S6.** Seed of *Trianthema monogynum*. (A) 50× magnification, (B) 300× magnification.



 **Figure S7.** Seed of *Trianthema portulacastrum*. (A) 70× magnification, (B) 300× magnification.





**Figure S8.** Seed of *Trianthema sanguineum*. (A) 70× magnification, (B) 300× magnification.





**Figure S9.** Seed of *Trianthema triquetrum*. (A) 70× magnification, (B) 300× magnification.





**Figure S10.** Seed of *Trianthema rhynchocalyptrum*. (A) 50× magnification, (B) 300× magnification.





**Figure S11.** Seed of *Zaleya govindia*. (A) 50× magnification, (B) 300× magnification.





**Figure S12.** Seed of *Gunniopsis calcarea*. (A) 70× magnification, (B) 300× magnification.





**Figure S13.** Seed of *Gunniopsis quadrifida*. (A) 70× magnification, (B) 300× magnification.





**Figure S14.** Seed of *Aizoanthemum dinteri*. (A) 70× magnification, (B) 300× magnification.

**

**

**Figure S15.** Seed of *Aizoon* *canariense*. (A) 70× magnification, (B) 300× magnification.





**Figure S16.** Seed of *Aizoon fruticosum*. (A) 70× magnification, (B) 300× magnification.





**Figure S17.** Seed of *Aizoon glinoides*. (A) 70× magnification, (B) 300× magnification.





**Figure S18.** Seed of *Aizoon pubescens*. (A) 70× magnification, (B) 300× magnification.





**Figure S19.** Seed of *Aizoon rigidum*. (A) 70× magnification, (B) 300× magnification.





**Figure S20.** Seed of *Aizoon secundum*. (A) 70× magnification, (B) 300× magnification.





**Figure S21.** Seed of *Aizoon sericeum*. (A) 70× magnification, (B) 300× magnification, (C) 5000× magnification.





**Figure S22.** Seed of *Aizoon virgatum*. (A) 70× magnification, (B) 300× magnification.





**Figure S23.** Seed of *Mesembryanthemum barklyi*. (A) 70× magnification, (B) 300× magnification, (C) 5000× magnification.





**Figure S24.** Seed of *Mesembryanthemum clandestinum*. (A) 70× magnification, (B) 300× magnification, (C) 5000× magnification.





**Figure S25.** Seed of *Mesembryanthemum cordifolium*. (A) 70× magnification, (B) 300× magnification, (C) 5000× magnification.





**Figure S26.** Seed of *Mesembryanthemum coriarium*. (A) 70× magnification, (B) 300× magnification.





**Figure S27.** Seed of *Mesembryanthemum crystallinum*. (A) 70× magnification, (B) 300× magnification, (C) 5000× magnification.





**Figure S28.** Seed of *Mesembryanthemum kuntzei*. (A) 70× magnification, (B) 300× magnification.





**Figure S29.** Seed of *Mesembryanthemum splendens*. (A) 50× magnification, (B) 300× magnification, (C) 5000× magnification.





**Figure S30.** Seed of *Mesembryanthemum tetragonum*. (A) 70× magnification, (B) 300× magnification, (C) 5000× magnification.





**Figure S31.** Seed of *Acrosanthes humifusa*. (A) 50× magnification, (B) 300× magnification, (C) 5000× magnification.





**Figure S32.** Seed of *Hymenogyne conica*. (A) 50× magnification, (B) 300× magnification.





**Figure S33.** Seed of *Skiatophytum skiatophytoides*. (A) 30× magnification, (B) 300× magnification.





**Figure S34.** Seed of *Cleretum booysenii*. (A) 70× magnification, (B) 300× magnification, (C) 5000× magnification.





**Figure S35.** Seed of *Cleretum herrei*. (A) 70× magnification, (B) 300× magnification, (C) 5000× magnification.





**Figure S36.** Seed of *Cleretum lyratifolium*. (A) 70× magnification, (B) 300× magnification, (C) 5000× magnification.





**Figure S37.** Seed of *Cleretum paterson-jonesii*. (A) 70× magnification, (B) 300× magnification, (C) 5000× magnification.





**Figure S38.** Seed of *Cleretum pinnatifidum*. (A) 70× magnification, (B) 300× magnification, (C) 5000× magnification.





**Figure S39.** Seed of *Drosanthemum asperulum*. (A) 70× magnification, (B) 300× magnification.





**Figure S40.** Seed of *Drosanthemum bicolor*. (A) 70× magnification, (B) 300× magnification, (C) 5000× magnification.





**Figure S41.** Seed of *Drosanthemum dejagerae*. (A) 70× magnification, (B) 300× magnification, (C) 5000× magnification.





**Figure S42.** Seed of *Drosanthemum lavisii*. (A) 70× magnification, (B) 300× magnification, (C) 5000× magnification.





**Figure S43.** Seed of *Antimima hantamensis*. (A) 70× magnification, (B) 300× magnification, (C) 5000× magnification.





**Figure S44.** Seed of *Antimima solida*. (A) 70× magnification, (B) 300× magnification, (C) 5000× magnification.





**Figure S45.** Seed of *Argyroderma delaetii*. (A) 70× magnification, (B) 300× magnification, (C) 5000× magnification.





**Figure S46.** Seed of *Carpobrotus acinaciformis*. (A) 70× magnification, (B) 300× magnification.





**Figure S47.** Seed of *Cheiridopsis alba-oculata*. (A) 70× magnification, (B) 300× magnification.





**Figure S48.** Seed of *Conophytum maughanii*. (A) 70× magnification, (B) 300× magnification.





**Figure S49.** Seed of *Deilanthe thudichumii*. (A) 70× magnification, (B) 300× magnification, (C) 5000× magnification.





**Figure S50.** Seed of *Delosperma bosseranum*. (A) 70× magnification, (B) 300× magnification.





**Figure S51.** Seed of *Dracophilus delaetianum*. (A) 70× magnification, (B) 300× magnification, (C) 5000× magnification.





**Figure S52.** Seed of *Eberlanzia sedoides*. (A) 70× magnification, (B) 300× magnification, (C) 5000× magnification.





**Figure S53.** Seed of *Ebracteola wilmaniae*. (A) 70× magnification, (B) 300× magnification, (C) 5000× magnification.





**Figure S54.** Seed of *Erepsia anceps*. (A) 70× magnification, (B) 300× magnification, (C) 5000× magnification.





**Figure S55.** Seed of *Gibbaeum album*. (A) 70× magnification, (B) 300× magnification.





**Figure S56.** Seed of *Lampranthus explanatus*. (A) 70× magnification, (B) 300× magnification, (C) 5000× magnification.





**Figure S57.** Seed of *Lampranthus reptans*. (A) 70× magnification, (B) 300× magnification, (C) 5000× magnification.





**Figure S58.** Seed of *Lampranthus watermeyeri*. (A) 70× magnification, (B) 300× magnification, (C) 5000× magnification.





**Figure S59.** Seed of *Lapidaria margaretae*. (A) 70× magnification, (B) 300× magnification, (C) 5000× magnification.



**Figure S60.** Seed of *Leipoldtia frutescens*. (A) 70× magnification, (B) 300× magnification, (C) 5000× magnification.





**Figure S61.** Seed of *Leipoldtia nevillei*. (A) 70× magnification, (B) 300× magnification.





**Figure S62.** Seed of *Lithops ruschiorum*. (A) 70× magnification, (B) 300× magnification, (C) 5000× magnification.





**Figure S63.** Seed of *Malephora crassa*. (A) 70× magnification, (B) 300× magnification, (C) 5000× magnification.





**Figure S64.** Seed of *Mestoklema arboriforme*. (A) 70× magnification, (B) 300× magnification, (C) 5000× magnification.





**Figure S65.** Seed of *Mitrophyllum dissitum*. (A) 70× magnification, (B) 300× magnification, (C) 5000× magnification.





**Figure S66.** Seed of *Namibia ponderosa*. (A) 70× magnification, (B) 300× magnification, (C) 5000× magnification.





**Figure S67.** Seed of *Oscularia deltoides*. (A) 70× magnification, (B) 300× magnification, (C) 5000× magnification.





**Figure S68.** Seed of *Peersia vanheerdei*. (A) 70× magnification, (B) 300× magnification, (C) 5000× magnification.





**Figure S69.** Seed of *Pleiospilos compactus*. (A) 70× magnification, (B) 300× magnification.





**Figure S70.** Seed of *Ruschia caroli*. (A) 70× magnification, (B) 300× magnification, (C) 5000× magnification.





**Figure S71.** Seed of *Ruschia costata*. (A) 70× magnification, (B) 300× magnification, (C) 5000× magnification.





**Figure S72.** Seed of *Ruschia dichroa*. (A) 70× magnification, (B) 300× magnification, (C) 5000× magnification.





**Figure S73.** Seed of *Ruschia grisea*. (A) 70× magnification, (B) 300× magnification.





**Figure S74.** Seed of *Ruschia lineolata*. (A) 100× magnification, (B) 500× magnification, (C) 5000× magnification.





**Figure S75.** Seed of *Ruschia multiflora*. (A) 70× magnification, (B) 300× magnification, (C) 5000× magnification.





**Figure S76.** Seed of *Ruschia rupicola*. (A) 70× magnification, (B) 300× magnification, (C) 5000× magnification.





**Figure S77.** Seed of *Ruschia spinosa*. (A) 70× magnification, (B) 300× magnification.





**Figure S78.** Seed of *Ruschia tenella*. (A) 70× magnification, (B) 300× magnification, (C) 5000× magnification.





**Figure S79.** Seed of *Ruschiella lunulata*. (A) 70× magnification, (B) 300× magnification, (C) 5000× magnification.





**Figure S80.** Seed of *Scopelogena bruynsii*. (A) 70× magnification, (B) 300× magnification, (C) 5000× magnification.





**Figure S81.** Seed of *Smicrostigma viride*. (A) 70× magnification, (B) 300× magnification, (C) 5000× magnification.





**Figure S82.** Seed of *Stayneria neilii*. (A) 70× magnification, (B) 300× magnification, (C) 5000× magnification.





**Figure S83.** Seed of *Stoeberia carpii*. (A) 70× magnification, (B) 300× magnification, (C) 5000× magnification.





**Figure S84.** Seed of *Vanheerdia roodiae*. (A) 70× magnification, (B) 300× magnification, (C) 5000× magnification.





**Figure S85.** Seed of *Vanzijlia annulata*. (A) 70× magnification, (B) 300× magnification, (C) 5000× magnification.
