# Supplementary material for: Evolution of seed characters and of dispersal modes in Aizoaceae
Source: Front Plant Sci. 2023 Mar 22;14:1140069. doi: 10.3389/fpls.2023.1140069 (PMC10073613; doi:10.3389/fpls.2023.1140069)
Supplement: Supplementary File 1 — Material used for the carpological examination. [file DataSheet_1.zip › Data Sheet/Supplementary file 7.docx]

**Evolution of seed characters and of dispersal modes in Aizoaceae**

**Alexander P. Sukhorukov*, Maya V. Nilova, Yuri Mazei, Maria Kushunina, Cornelia Klak**

*** Correspondence:** Corresponding author: suchor@mail.ru

Supplementary file 5

# Images of the seed-coat of investigated Aizoaceae species


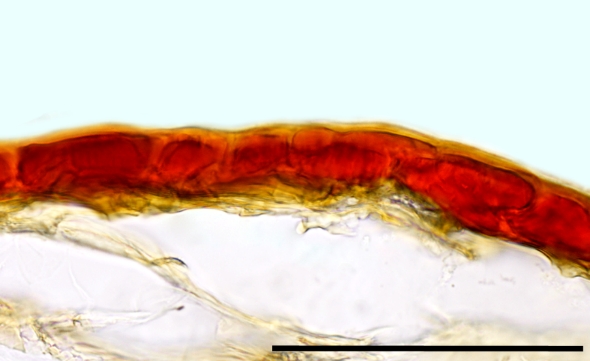


**Figure S1.** Seed-coat of *Anisostigma schenckii*. Scale bar – 100 µm.


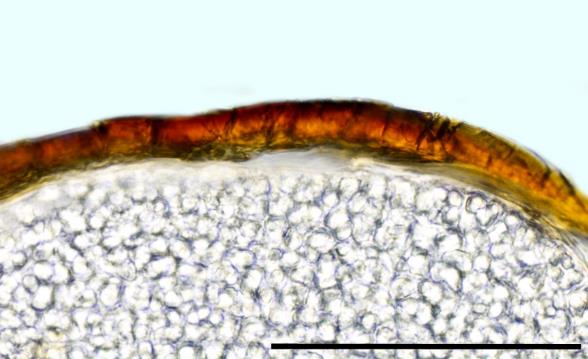


**Figure S2.** Seed-coat of *Sesuvium humifusum*. Scale bar – 100 µm.


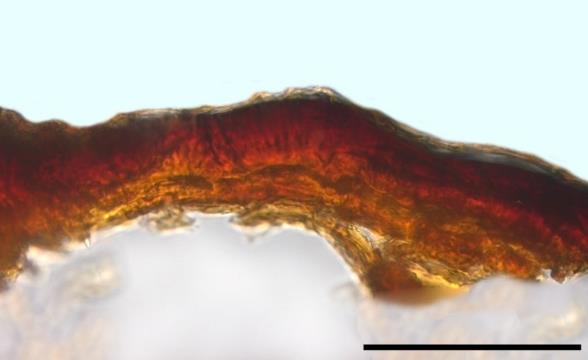


**Figure S3.** Seed-coat of *Sesuvium portulacastrum*. Scale bar – 100 µm.


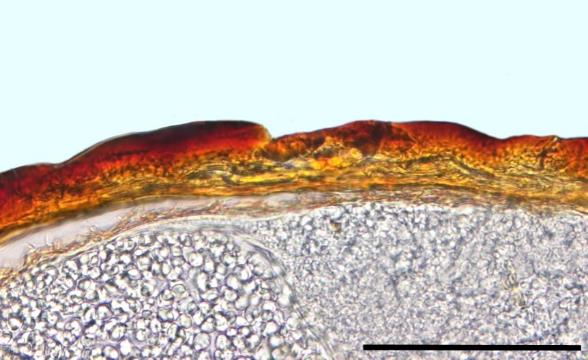


**Figure S4.** Seed-coat of *Sesuvium revolutifolium*. Scale bar – 100 µm.


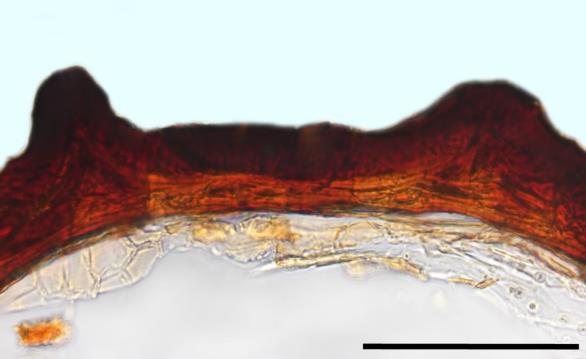


**Figure S5.** Seed-coat of *Trianthema triquetrum*. Scale bar – 100 µm.


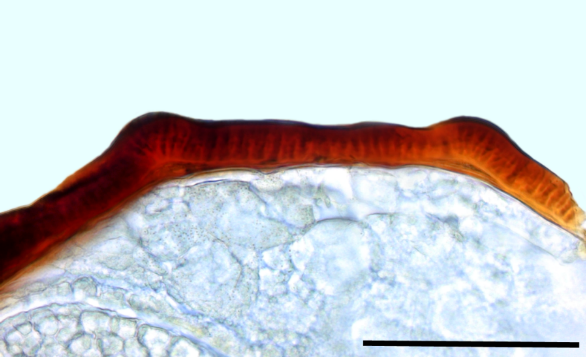


**Figure S6.** Seed-coat of *Aizoon canariense*. Scale bar – 100 µm.


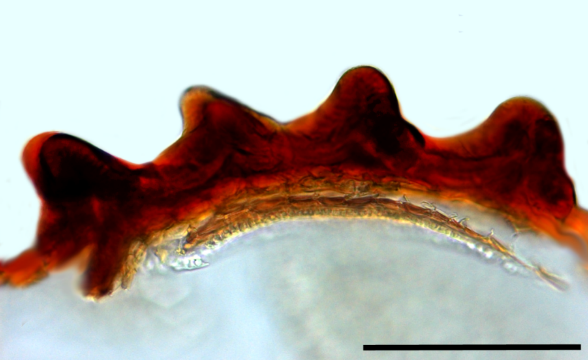


**Figure S7.** Seed-coat of *Aizoon glinoides*. Scale bar – 100 µm.


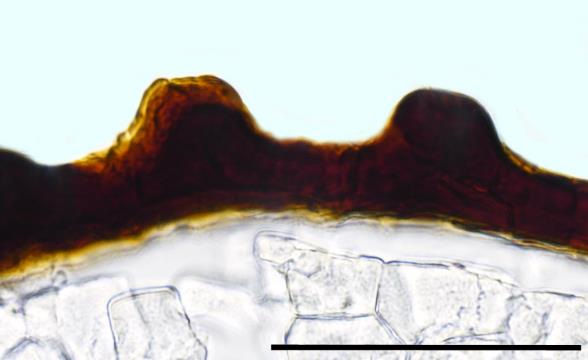


**Figure S8.** Seed-coat of *Aizoon pubescens*. Scale bar – 100 µm.


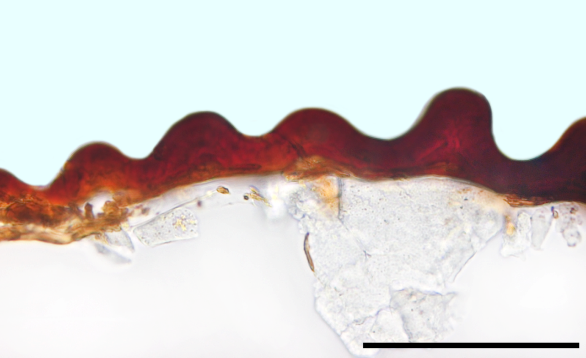


**Figure S9.** Seed-coat of *Aizoon rigidum*. Scale bar – 100 µm.


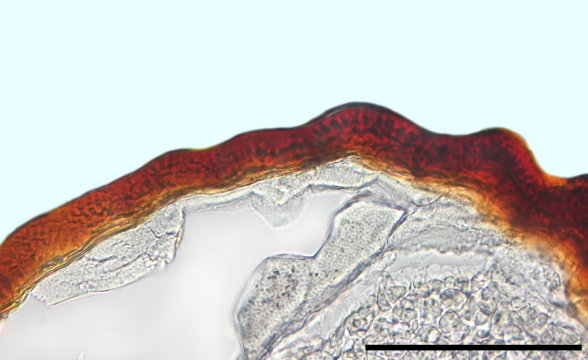


**Figure S10.** Seed-coat of *Aizoon sericeum*. Scale bar – 100 µm.


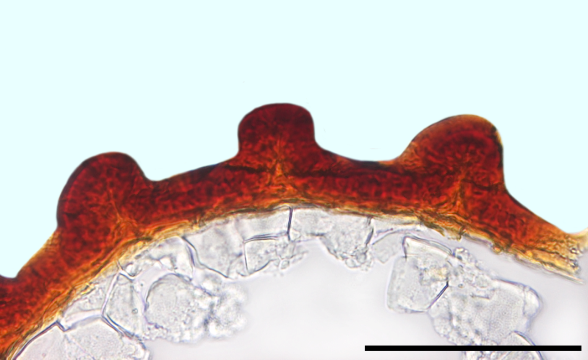


**Figure S11.** Seed-coat of *Aizoon virgatum*. Scale bar – 100 µm.


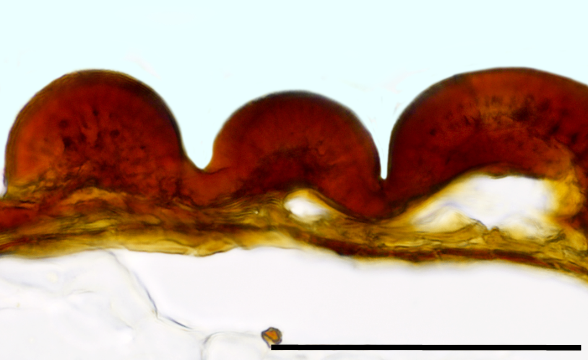


**Figure S12.** Seed-coat of *Gunniopsis calcarea*. Scale bar – 100 µm.


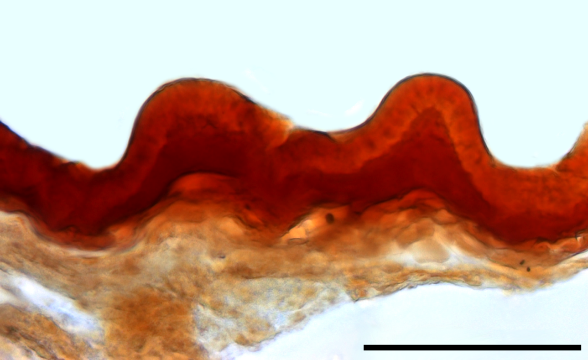


**Figure S13.** Seed-coat of *Gunniopsis quadrifida*. Scale bar – 100 µm.


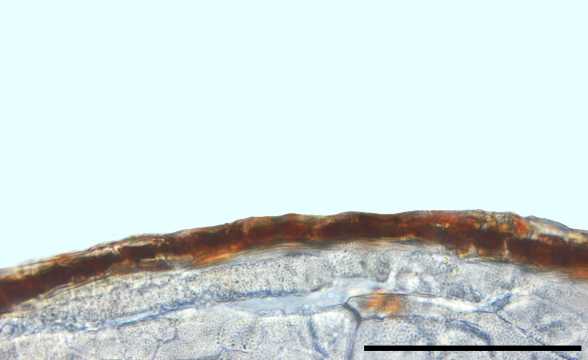


**Figure S14.** Seed-coat of *Tetragonia tetragonoides*. Scale bar – 100 µm.


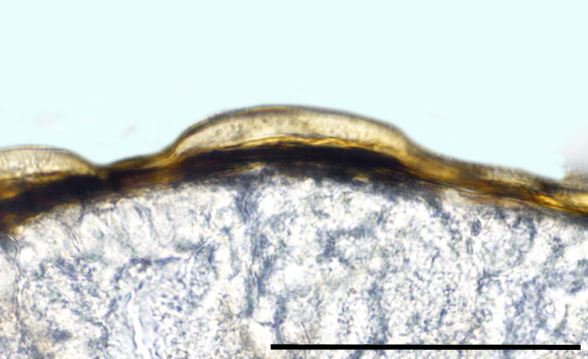


**Figure S15.** Seed-coat of *Mesembryanthemum barklyi*. Scale bar – 100 µm.

_
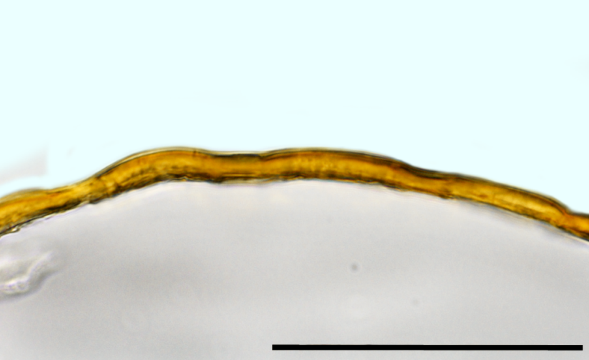
_

**Figure S16.** Seed-coat of *Mesembryanthemum clandestinum*. Scale bar – 100 µm.


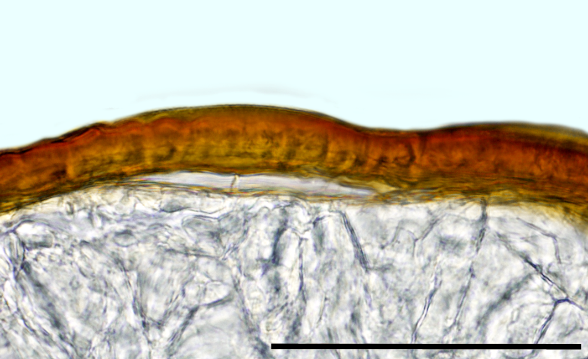


**Figure S17.** Seed-coat of *Mesembryanthemum coriarium*. Scale bar – 100 µm.


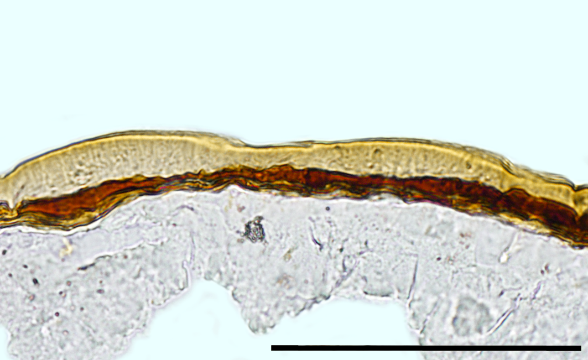


**Figure S18.** Seed-coat of *Mesembryanthemum kuntzei*. Scale bar – 100 µm.


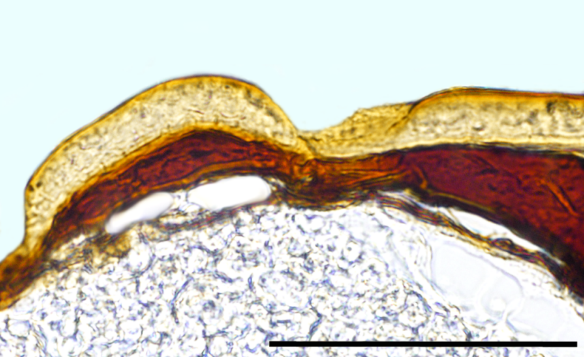


**Figure S19.** Seed-coat of *Mesembryanthemum splendens* ssp. *splendens*. Scale bar – 100 µm.


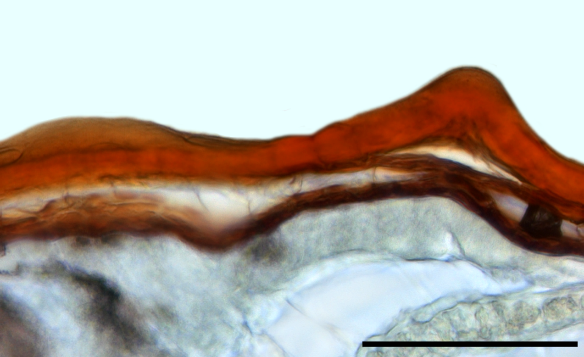


**Figure S20.** Seed-coat of *Mesembryanthemum tetragonum*. Scale bar – 100 µm.


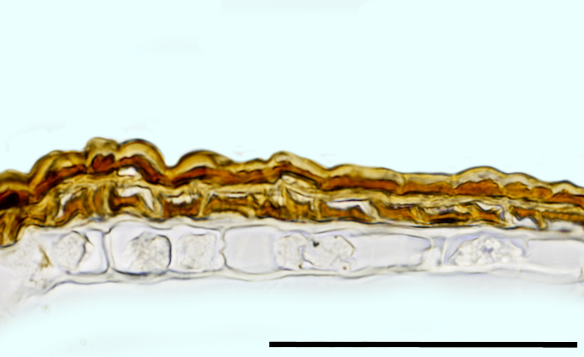


**Figure S21.** Seed-coat of *Hymenogyne conica*. Scale bar – 100 µm.


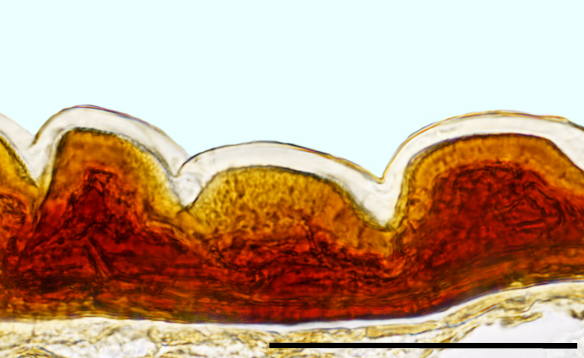


**Figure S22.** Seed-coat of *Skiatophytum skiatophytoides*. Scale bar – 100 µm.


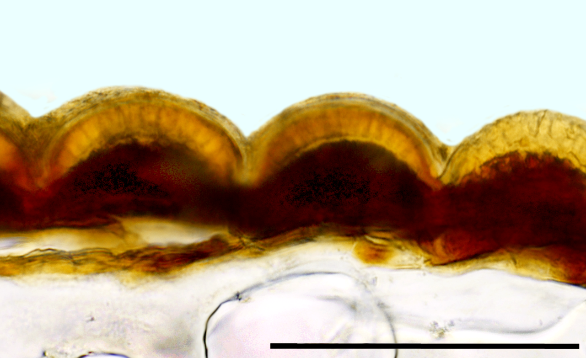


**Figure S23.** Seed-coat of *Cleretum booysenii*. Scale bar – 100 µm.


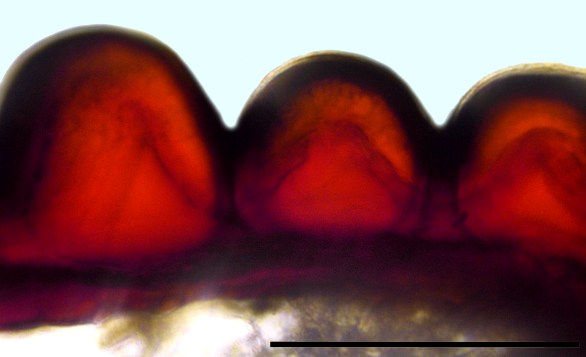


**Figure S24.** Seed-coat of *Cleretum herrei*. Scale bar – 100 µm.


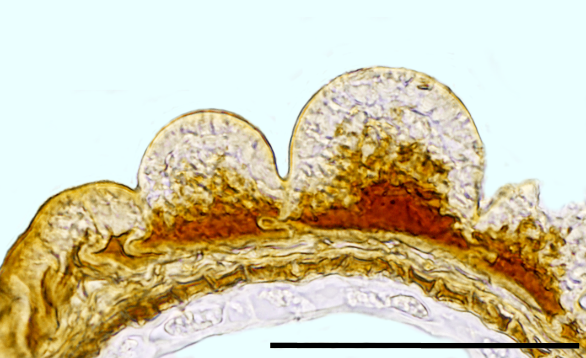


**Figure S25.** Seed-coat of *Cleretum lyratifolium*. Scale bar – 100 µm.


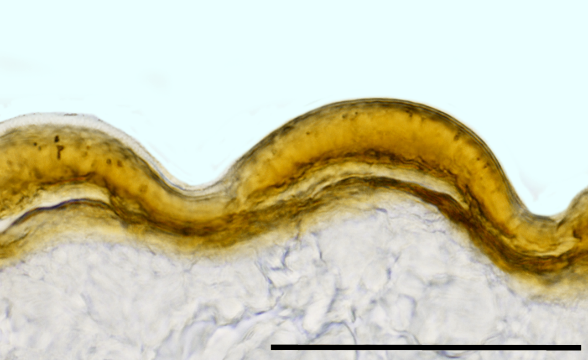


**Figure S26.** Seed-coat of *Cleretum paterson-jonesii*. Scale bar – 100 µm.


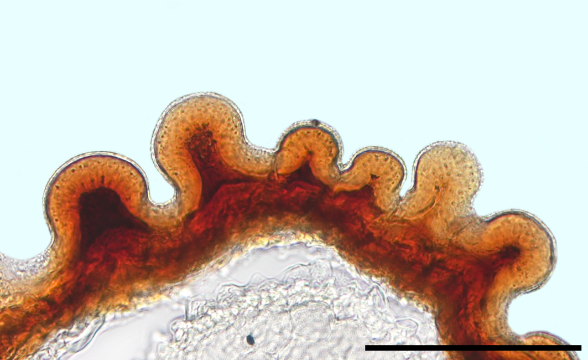


**Figure S27.** Seed-coat of *Cleretum pinnatifidum*. Scale bar – 100 µm.


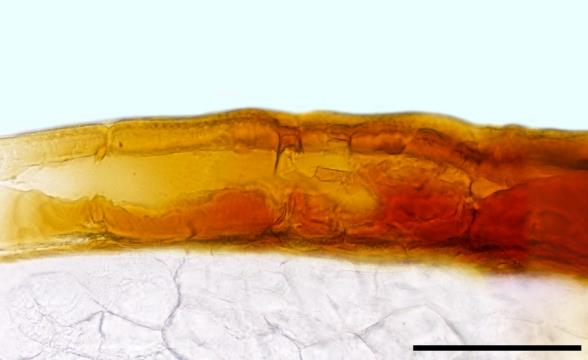


**Figure S28.** Seed-coat of *Drosanthemum asperulum*. Scale bar – 100 µm.


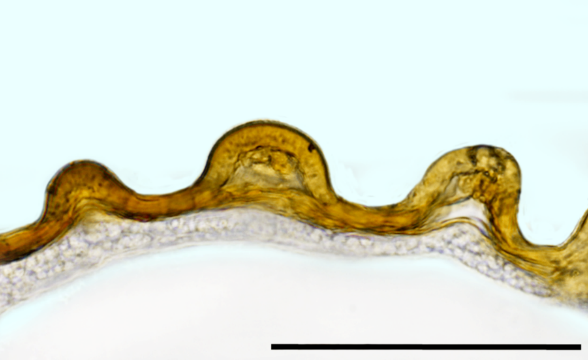


**Figure S29.** Seed-coat of *Drosanthemum bicolor*. Scale bar – 100 µm.


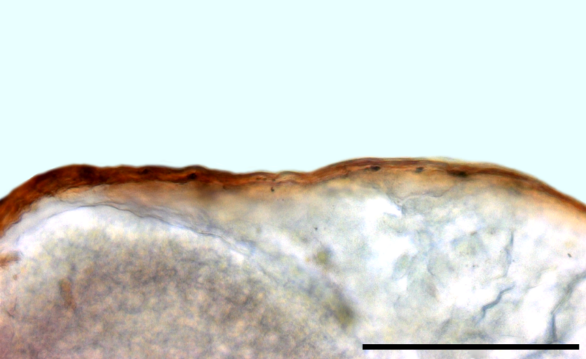


**Figure S30.** Seed-coat of *Drosanthemum dejagerae*. Scale bar – 100 µm.


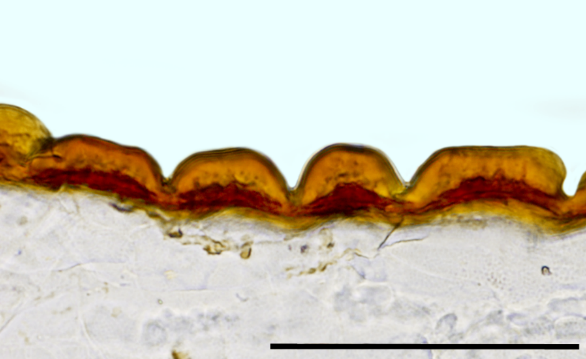


**Figure S31.** Seed-coat of *Drosanthemum lavisii*. Scale bar – 100 µm.


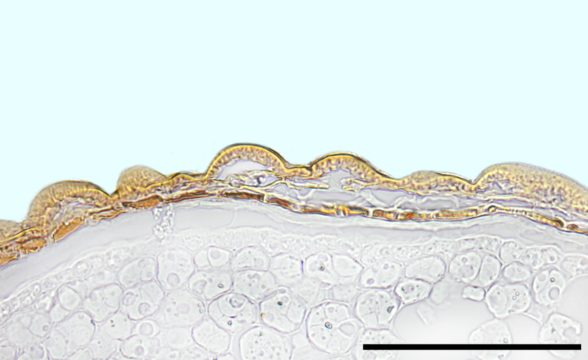


**Figure S32.** Seed-coat of *Antimima hantamensis*. Scale bar – 100 µm.


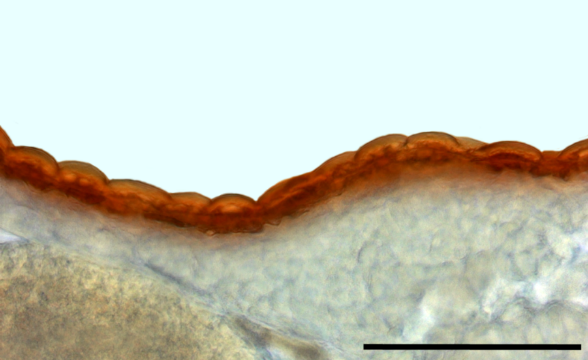


**Figure S33.** Seed-coat of *Antimima solida*. Scale bar – 100 µm.


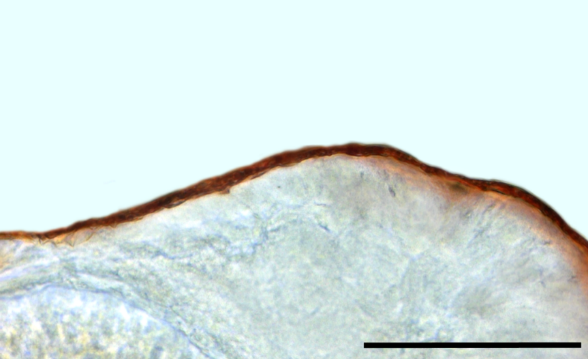


**Figure S34.** Seed-coat of *Argyroderma delaetii*. Scale bar – 100 µm.


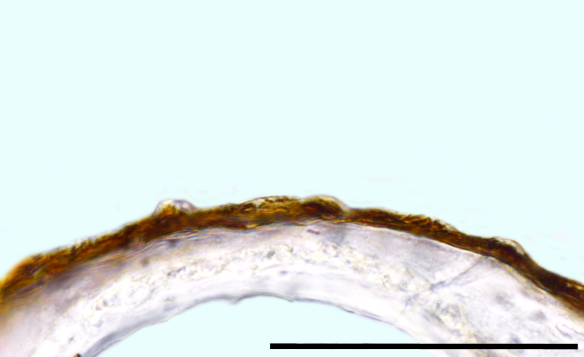


**Figure S35.** Seed-coat of *Conophytum maughanii*. Scale bar – 100 µm.


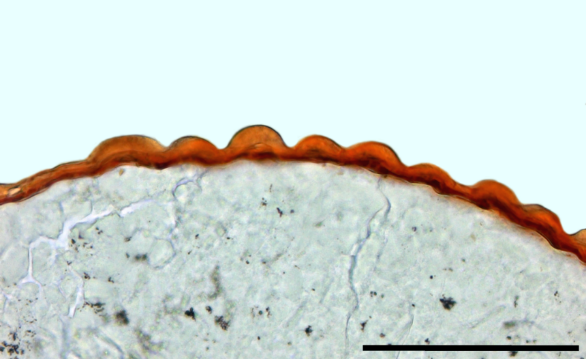


**Figure S36.** Seed-coat of *Deilanthe thudichumii*. Scale bar – 100 µm.


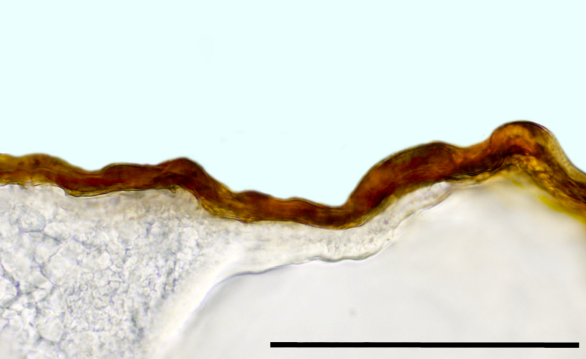


**Figure S37.** Seed-coat of *Delosperma* sp. Scale bar – 100 µm.


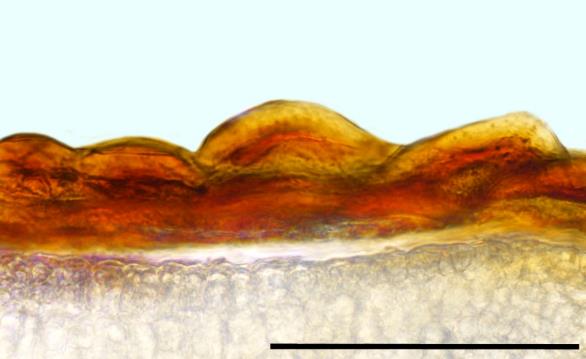


**Figure S38.** Seed-coat of *Delosperma bosseranum*. Scale bar – 100 µm.


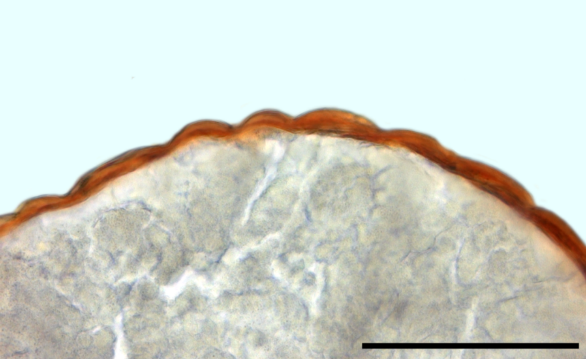


**Figure S39.** Seed-coat of *Dracophilus delaetianum*. Scale bar – 100 µm.


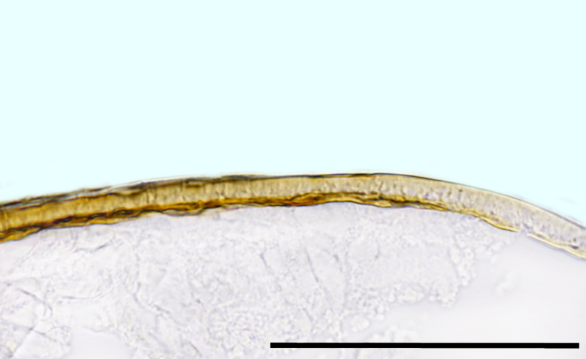


**Figure S40.** Seed-coat of *Eberlanzia sedoides*. Scale bar – 100 µm.


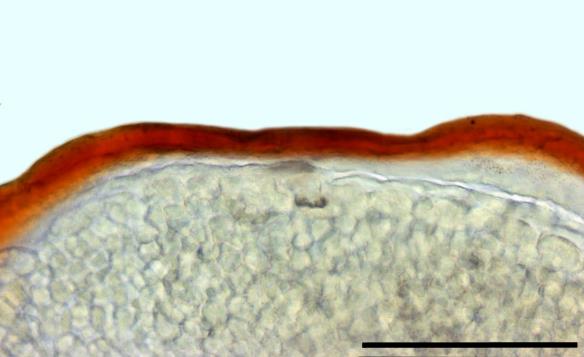


**Figure S41.** Seed-coat of *Ebracteola wilmaniae*. Scale bar – 100 µm.


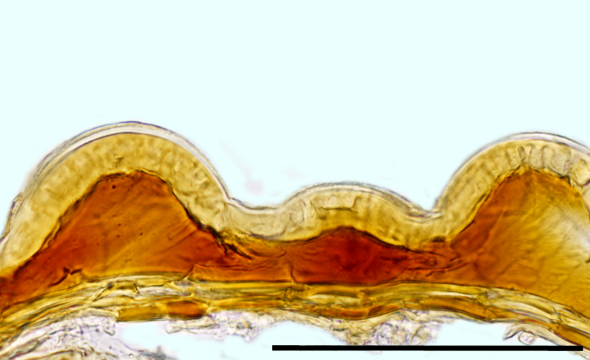


**Figure S42.** Seed-coat of *Erepsia anceps*. Scale bar – 100 µm.


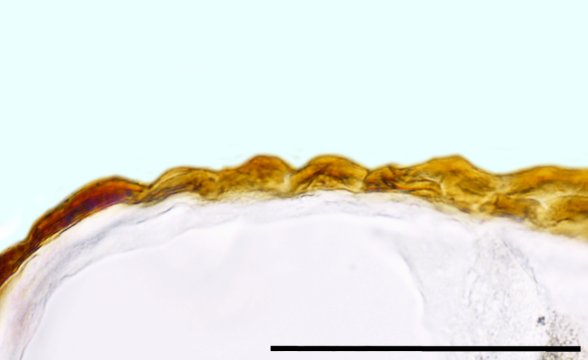


**Figure S43.** Seed-coat of *Gibbaeum album*. Scale bar – 100 µm.


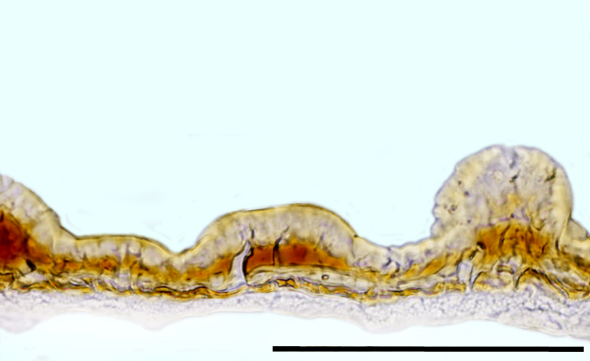


**Figure S44.** Seed-coat of *Lampranthus explanatus*. Scale bar – 100 µm.


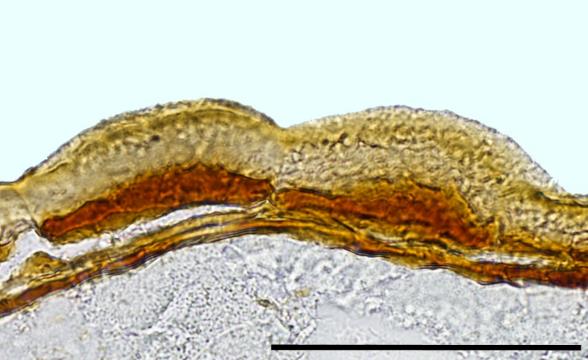


**Figure S45.** Seed-coat of *Lampranthus reptans*. Scale bar – 100 µm.


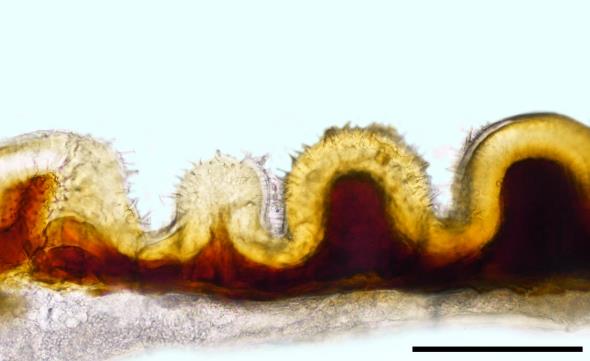


**Figure S46.** Seed-coat of *Lampranthus watermeyeri*. Scale bar – 100 µm.


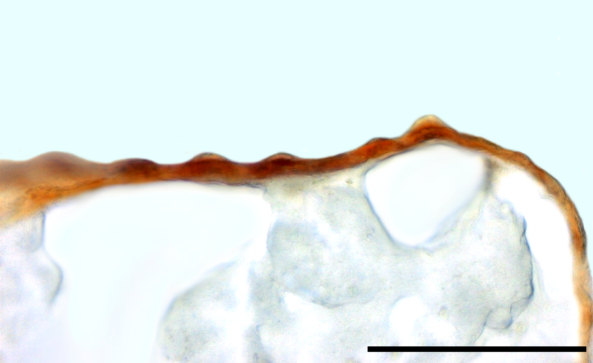


**Figure S47.** Seed-coat of *Lapidaria margaretae*. Scale bar – 100 µm.


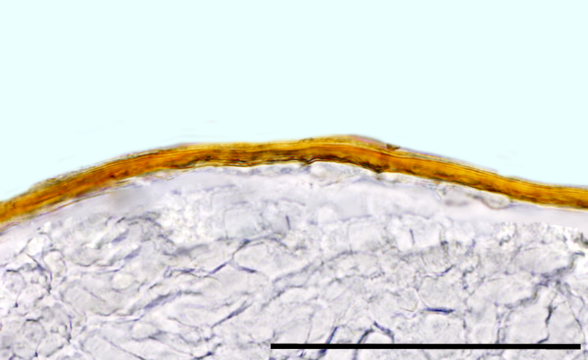


**Figure S48.** Seed-coat of *Leipoldtia frutescens*. Scale bar – 100 µm.


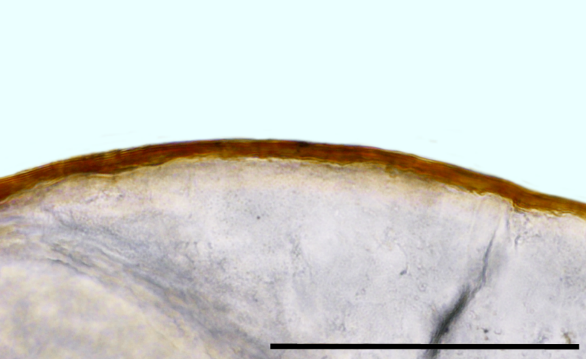


**Figure S49.** Seed-coat of *Leipoldtia nevillei*. Scale bar – 100 µm.


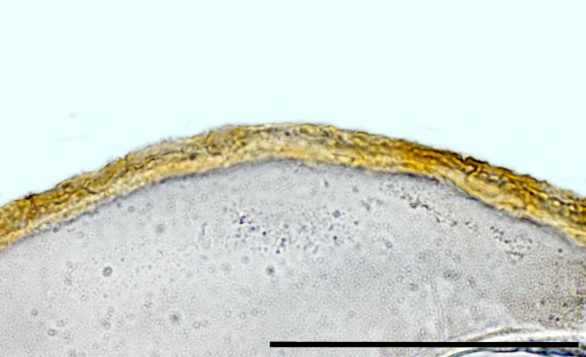


**Figure S50.** Seed-coat of *Lithops ruschiorum*. Scale bar – 100 µm.


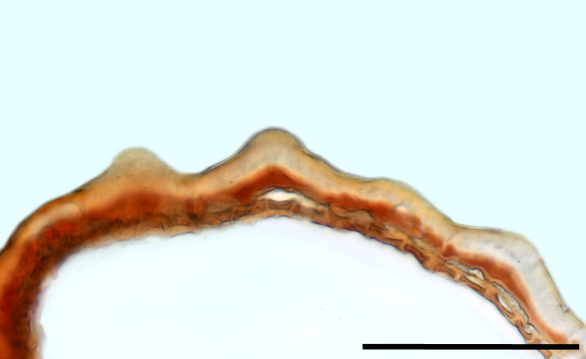


**Figure S51.** Seed-coat of *Malephora crassa*. Scale bar – 100 µm.


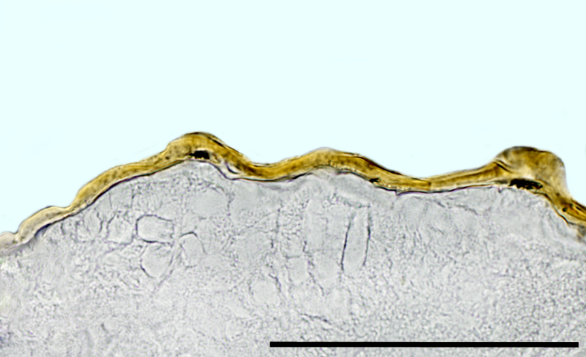


**Figure S52.** Seed-coat of *Mestoklema arboriforme*. Scale bar – 100 µm.


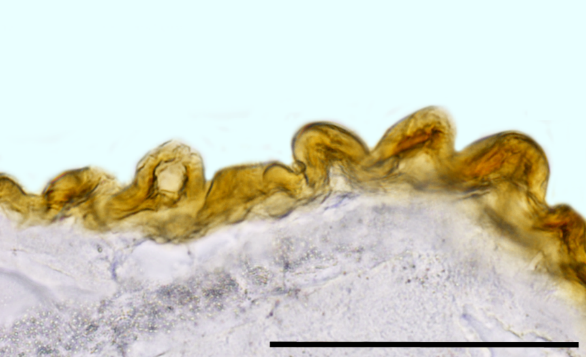


**Figure S53.** Seed-coat of *Mitrophyllum dissitum*. Scale bar – 100 µm.


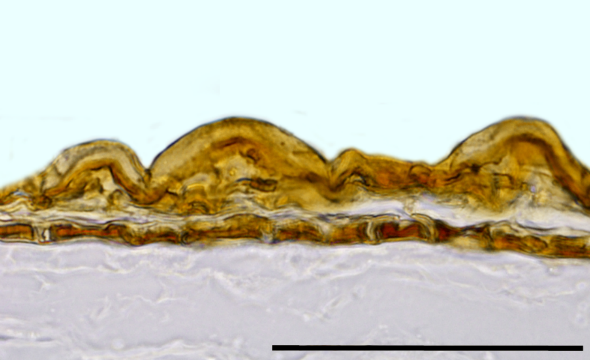


**Figure S54.** Seed-coat of *Namibia ponderosa*. Scale bar – 100 µm.


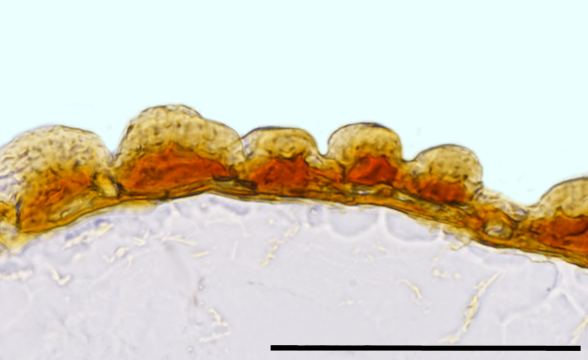


**Figure S55.** Seed-coat of *Oscularia deltoides*. Scale bar – 100 µm.


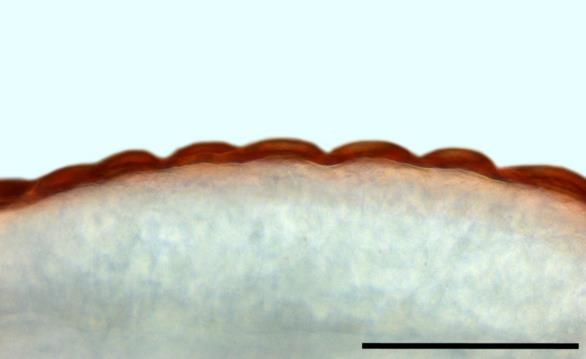


**Figure S56.** Seed-coat of *Peersia vanheerdei*. Scale bar – 100 µm.


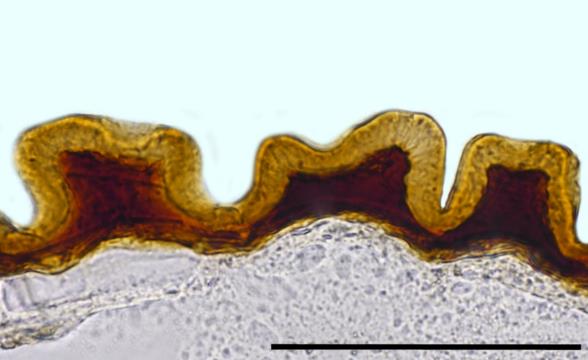


**Figure S57.** Seed-coat of *Pleiospilos compactus* ssp. *canus*. Scale bar – 100 µm.


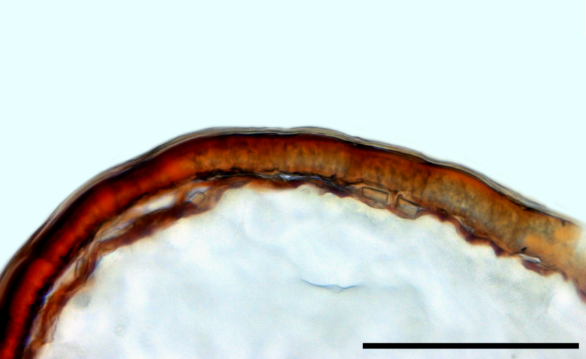


**Figure S58.** Seed-coat of *Ruschia caroli*. Scale bar – 100 µm.


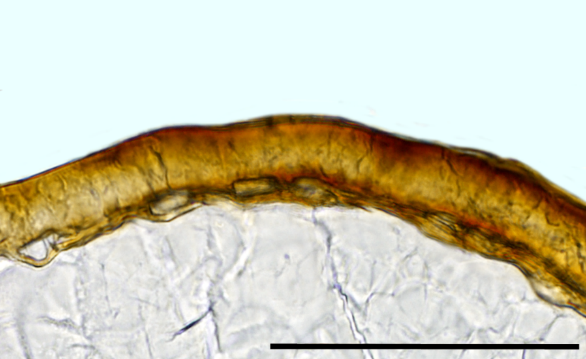


**Figure S59.** Seed-coat of *Ruschia costata*. Scale bar – 100 µm.


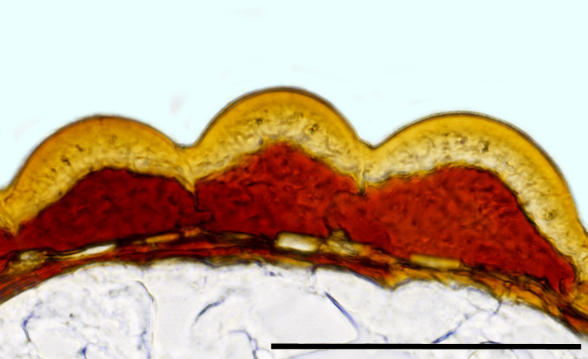


**Figure S60.** Seed-coat of *Ruschia dichroa*. Scale bar – 100 µm.


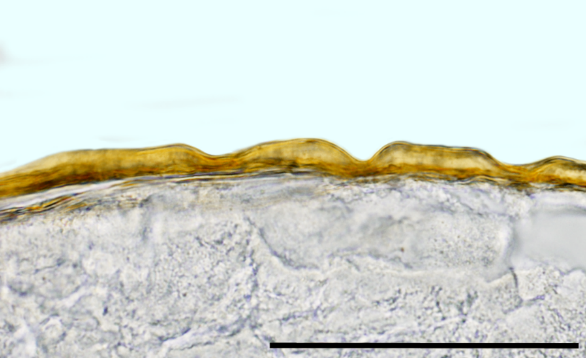


**Figure S61.** Seed-coat of *Ruschia grisea*. Scale bar – 100 µm.


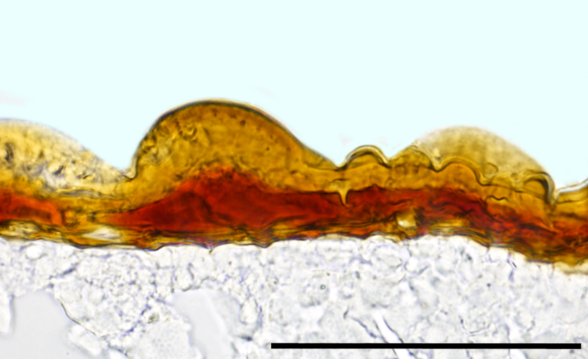


**Figure S62.** Seed-coat of *Ruschia lineolata*. Scale bar – 100 µm.


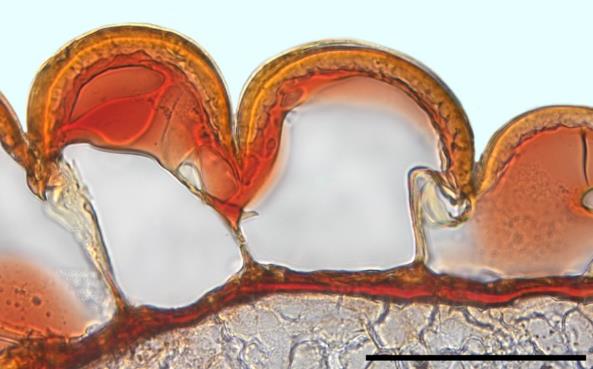


**Figure S63.** Seed-coat of *Ruschia multiflora*. Scale bar – 100 µm.


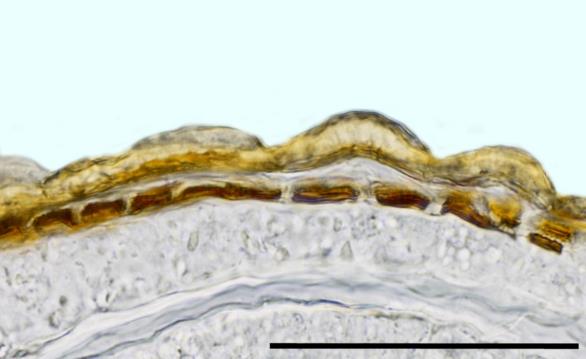


**Figure S64.** Seed-coat of *Ruschia rupicola*. Scale bar – 100 µm.


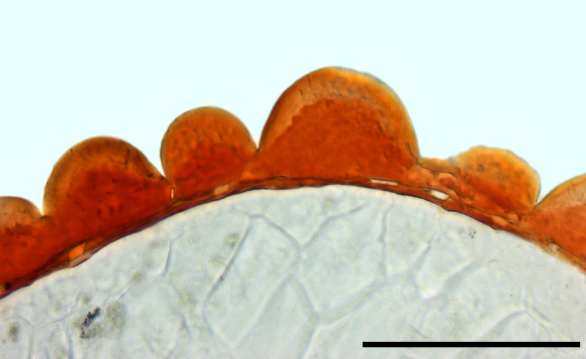


**Figure S65.** Seed-coat of *Ruschia tenella*. Scale bar – 100 µm.


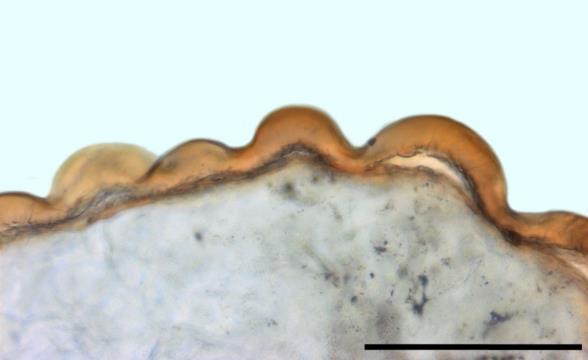


**Figure S66.** Seed-coat of *Ruschiella lunulata*. Scale bar – 100 µm.


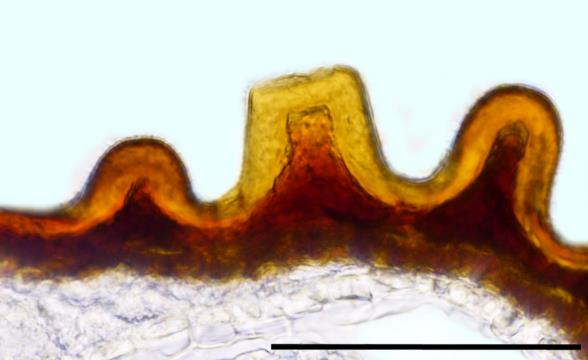


**Figure S67.** Seed-coat of *Scopelogena bruynsii*. Scale bar – 100 µm.


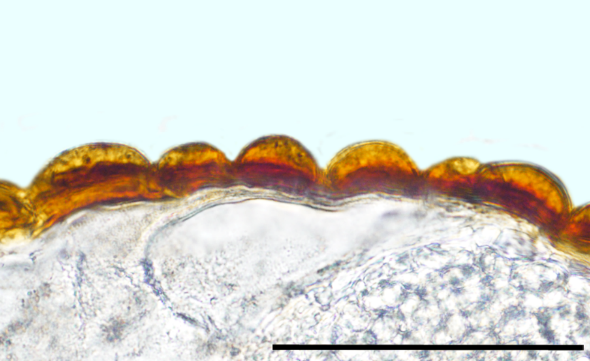


**Figure S68.** Seed-coat of *Smicrostigma viride*. Scale bar – 100 µm.


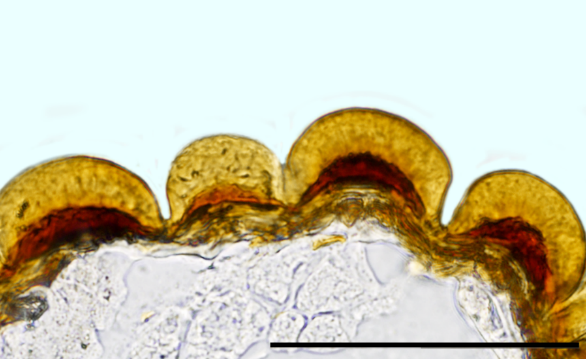


**Figure S69.** Seed-coat of *Stayneria neilii*. Scale bar – 100 µm.


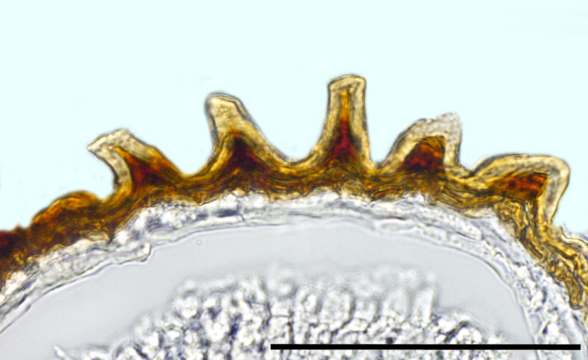


**Figure S70.** Seed-coat of *Stoeberia carpii*. Scale bar – 100 µm.


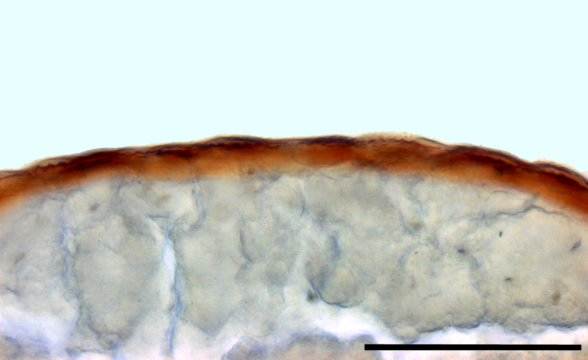


**Figure S71.** Seed-coat of *Vanheerdia roodiae*. Scale bar – 100 µm.


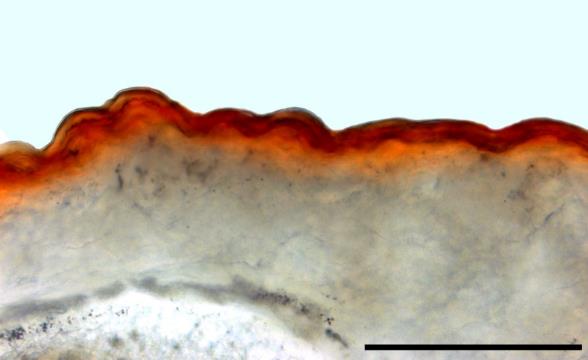


**Figure S72.** Seed-coat of *Vanzijlia annulata*. Scale bar – 100 µm.
