# Supplementary figures and images for: Evolution of seed characters and of dispersal modes in Aizoaceae
Source: Front Plant Sci. 2023 Mar 22;14:1140069. doi: 10.3389/fpls.2023.1140069 (PMC10073613; doi:10.3389/fpls.2023.1140069)

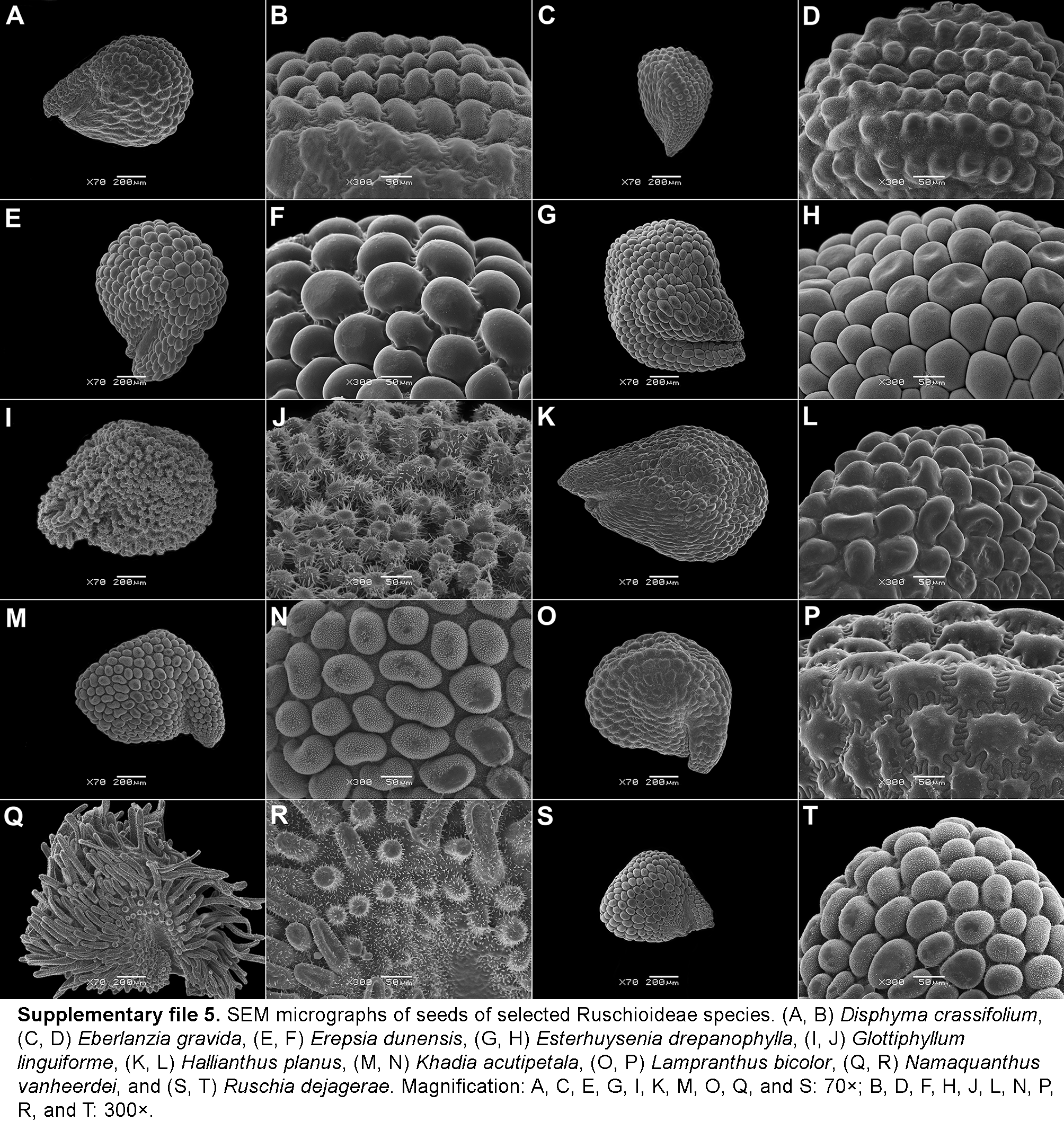

Supplement: Supplementary File 1 — Material used for the carpological examination. [file DataSheet_1.zip › Data Sheet/Supplementary file 5.tif]

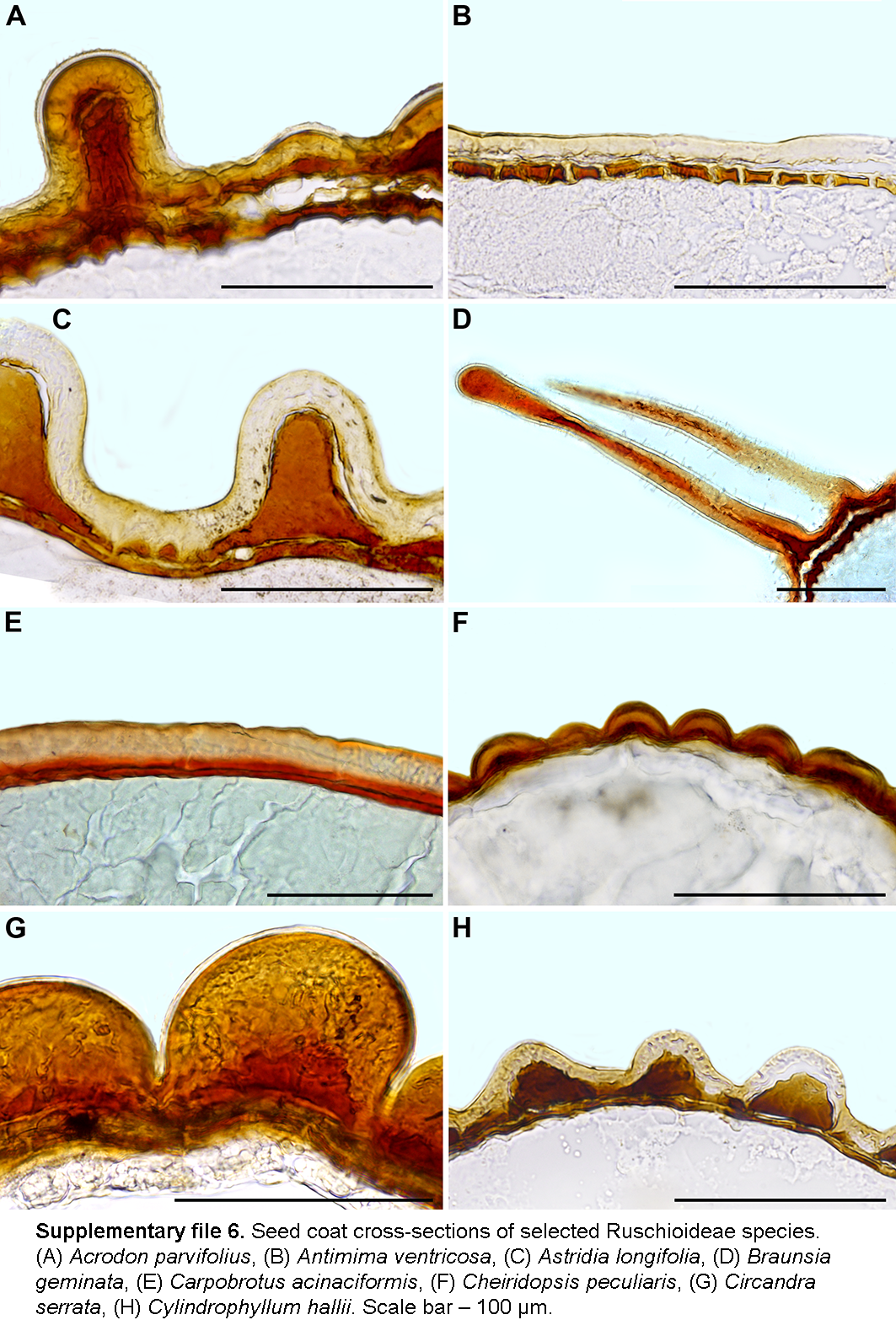

Supplement: Supplementary File 1 — Material used for the carpological examination. [file DataSheet_1.zip › Data Sheet/Supplementary file 6.tif]

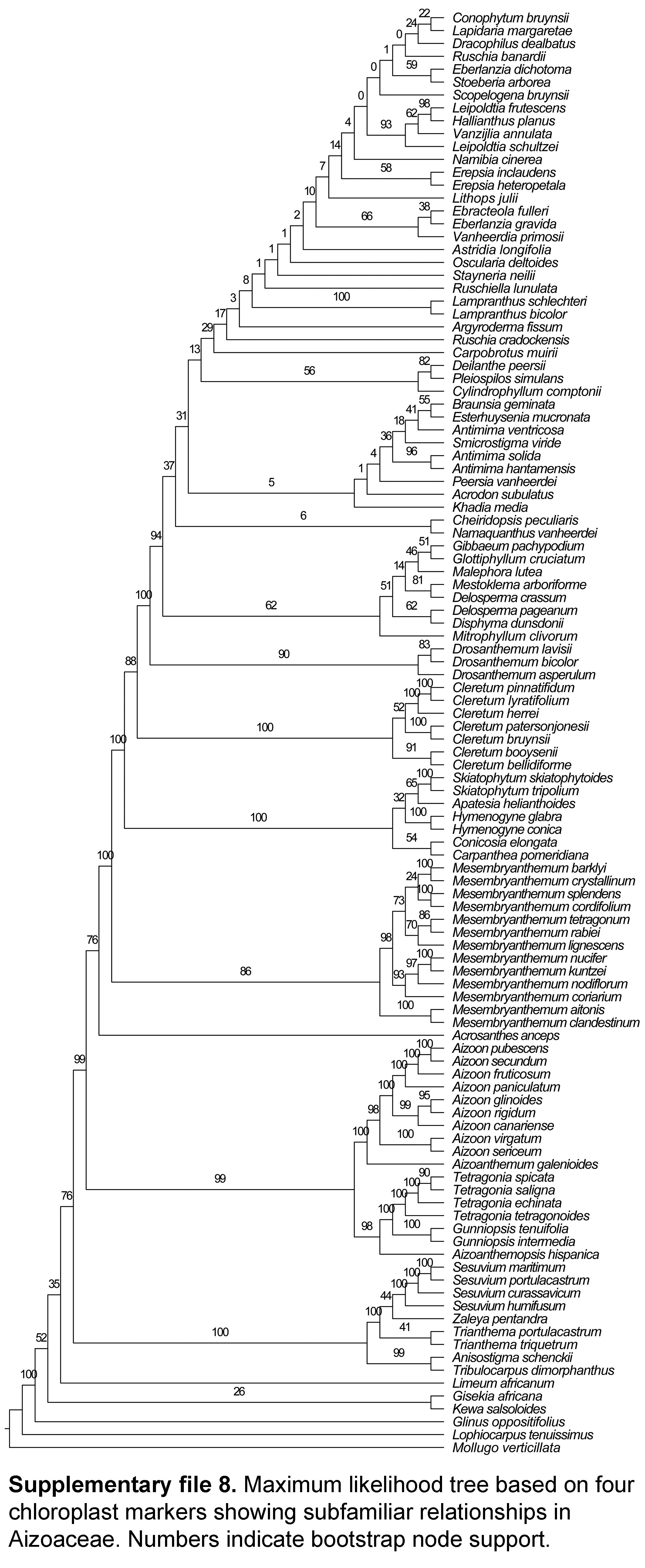

Supplement: Supplementary File 1 — Material used for the carpological examination. [file DataSheet_1.zip › Data Sheet/Supplementary file 8.tif]
