# Supplementary material for: Evolution of seed characters and of dispersal modes in Aizoaceae
Source: Front Plant Sci. 2023 Mar 22;14:1140069. doi: 10.3389/fpls.2023.1140069 (PMC10073613; doi:10.3389/fpls.2023.1140069)
Supplement: Supplementary File 1 — Material used for the carpological examination. [file DataSheet_1.zip › Data Sheet/Supplementary file 1.doc]

# *Supplementary Material*

# Evolution of seed characters and of dispersal modes in Aizoaceae

Alexander P. Sukhorukov*, Maya V. Nilova, Yuri Mazei, Maria Kushunina, Cornelia Klak

*** Correspondence:** Corresponding author: suchor@mail.ru

| **Supplementary file 1.** Material used for the carpological examination | | |
| --- | --- | --- |
| **Species** | **Country and Province** | **Voucher** |
| **Sesuvioideae: Tribe Anisostigmateae** |  |  |
| *Anisostigma schenckii* Schinz | 1) Namibia, 2) Namibia, Hardap Region | 1) *C. Klak 2634* (BOL); 2) *A. Sukhorukov 164* (MW0975142) |
| *Tribulocarpus dimorphanthus* (Pax) S.Moore | Namibia | *C. Klak 2441* (BOL) |
| *Tribulocarpus somalensis* (Engl.) Sukhor. | Somalia | *C.F. Hemming 3020* (K) |
| **Sesuvioideae: Tribe Sesuvieae** |  |  |
| *Sesuvium curassavicum* Sukhor. | Kingdom of the Netherland, Curaçao | *A. Sukhorukov 100* (MW0595676) |
| *Sesuvium humifusum* (Turpin) Bohley & G.Kadereit | Kingdom of the Netherland, Bonaire | *F. Arnoldo 987* (U1057838) |
| *Sesuvium maritimum* (Walter) Britton, Sterns & Poggenb. | 1) Cuba. Havana; 2) Cayman Islands, Grand Cayman Island | 1) *E.L. Ekman 13261* (G); 2) G.R. Proctor 15115 (BM); |
| *Sesuvium mezianum* (K.Müll.) Bohley &G.Kadereit | Paraguay | *B. Balansa 2263* (P04583791) |
| *Sesuvium portulacastrum* (L.) L. | India, Goa State | *A. Sukhorukov s.n.* (M) |
| *Sesuvium revolutifolium* Ortega | Cape Verde, São Vicente Island | *A. Sukhorukov 398* (MW0975135) |
| *Sesuvium rubriflorum* (Urb.) Bohley & G.Kadereit | Cuba, Matanzas Prov. | *E.L. Ekman 17016* (G) |
| *Trianthema crystallinum* (Forssk.) Vahl | Yemen, Abiyan | *L. Boulos 16803* (BM) |
| *Trianthema hereroense* Schinz | Namibia, Hardap Region | *A. Sukhorukov 315* (MW0975136) |
| *Trianthema monogynum* L. | Grenada | *A. Sukhorukov s.n.* (MW) |
| *Trianthema portulacastrum* L. | Cape Verde, Sal Island | *A. Sukhorukov s.n.* (MW0582876) |
| *Trianthema sanguineum* Volkens & Irmsch. | Tanzania, Arusha Region | *A. Sukhorukov s.n.* (MW) |
| *Trianthema triquetrum* Willd. | Sudan, Northern Prov. | *T. Ahti 16279* (H1078914) |
| *Zaleya govindia* (Buch.-Ham.) N.C.Nair | Pakistan, Sind | *K.H. Rechinger 28708* (W) |
| *Zaleya pentandra* (L.) C.Jeffrey | Namibia, Karas Region | *A. Sukhorukov 45* (MW0582874) |
| **Aizooideae** |  |  |
| *Aizoanthemopsis hispanica* (L.) Klak | Spain, Canary Islands | 1) *A. Juan & M. Juan AJ104-2* (SALA 129722 barcode, SALA); 2) E. Bourgeau 413 (P04621162) |
| *Aizoanthemum dinteri* (Schinz) Friedrich | Namibia | *K. Dinter s.n*. (BM) |
| *Aizoanthemum mossamedense* (Welw. ex Oliv.) Friedrich | Angola, Namibe prov. | *C. Klak 2551a* (BOL) |
| *Aizoon canariense* L. (South Africa) | 1) South Africa, Northern Cape, 2) Cape Verde, Sal Island | 1) *C. Klak 2636* (BOL), 2) *A*. *Sukhorukov 83* (MW0582190) |
| *Aizoon fruticosum* L.f. | Namibia | *T. Ahti s.n*. (H) |
| *Aizoon glinoides* Eckl. & Zeyh. | South Arica, Eastern Cape | *R.A. Dyer2129* (K) |
| *Aizoon paniculatum* L. | South Africa, Western Cape | *L. Nurish & O. Pekeur 1934* (K) |
| *Aizoon pubescens* Eckl. & Zeyh. | Australia, Flinders Ranges | *L. Haegi 595* (H114511) |
| *Aizoon rigidum* L.f. | South Africa, Eastern Cape | *F.W. Malantha & L.A. Nkuna 2450* (K) |
| *Aizoon secundum* L.f. | South Africa, Cape | *C. Moss 18039* (BM) |
| *Aizoon sericeum* (Pax) Klak | Namibia, Bodenhausen Camp | *R. Seydel 2712* (K) |
| *Aizoon virgatum* Welw. | Namibia, Karibib | *P. Olivier 2508* (K) |
| *Gunniopsis calcarea* Chinnock | West Australia | *A. Strid 21954* (K) |
| *Gunniopsis calva* Chinnock | Australia, Nonning Station | *R.L. Specht & B.B. Carrodus* (K) |
| *Gunniopsis papillata* Chinnock | South Australia, Eyre Lake | *J.Z. Weber 8825* (MHA) |
| *Gunniopsis quadrifida* (F.Muell.) Pax | Australia, Upper Eyre Peninsula | *R.L. Specht & B.B. Carrodus 109* (K) |
| *Tetragonia echinata* Aiton | South Africa, Western Cape | *C. Klak 2480* (BOL) |
| *Tetragonia saligna* Fenzl | South Africa, Western Cape | *C. Klak 2460* (BOL) |
| *Tetragonia spicata* L.f. | South Africa, Western Cape | *C. Klak 2325* (BOL) |
| *Tetragonia tetragonoides* (Pall.) Kuntze | 1) Chile, Valparaiso; 2) Israel, Esdraelon Plain | 1) *Mertens s.n.* (LE); 2) *A. Sukhorukov* s.n. (MW) |
| **Mesembryanthemoideae** |  |  |
| *Mesembryanthemum aitonis* Jacq. | South Africa, Western Cape | *C. Klak 2604* (BOL) |
| *Mesembryanthemum barklyi* N.E.Br. | South Africa, Northern Cape | *C. Klak 2569* (BOL) |
| *Mesembryanthemum clandestinum* Haw. | South Africa, Northern Cape | *C. Klak 2150* (BOL) |
| *Mesembryanthemum cordifolium* L.f. | Portugal, Madeira Island | *M. Isoviita s.n.* (H1090378) |
| *Mesembryanthemum coriarium* Burch. | South Africa, Northern Cape | *M.J.A. Werger 119* (U1057678) |
| *Mesembryanthemum crystallinum* L. | Israel, Netania | *A. Sukhorukov s.n.* (MW) |
| *Mesembryanthemum kuntzei* Schinz | Namibia | *M. Gerbaulet 50072* (BOL) |
| *Mesembryanthemum lignescens* (L.Bolus) Klak | South Africa, Northern Cape | *C. Klak 2637* (BOL) |
| *Mesembryanthemum nodiflorum* L. | 1) Egypt, Alexandria; 2) Israel, Lower Jordan valley | 1) *Feinbrun & Zohary s.n.* (HUJ); 2) *N. Naftolsky 337* (HUJ) |
| *Mesembryanthemum nucifer* (Ihlenf. & Bittrich) Klak | South Africa, Northern Cape | *S. Pierce 64* (BOL) |
| *Mesembryanthemum rabiei* (L.Bolus) Klak | South Africa, Free State | *C.A. Smith 4099* (BOL) |
| *Mesembryanthemum splendens* L. ssp. *splendens* | South Africa, Western Cape | *C. Klak 2566* (BOL) |
| *Mesembryanthemum tetragonum* Thunb. | Namibia, Aus | *K. Dinter 1106* (B100153179) |
| **Ruschioideae: Acrosanthoideae** |  |  |
| *Acrosanthes anceps* Sond. | South Africa, Western Cape | *C.L. Zeyher 721* (LE) |
| *Acrosanthes humifusa* Sond. | South Africa, Western Cape | *R. Adamson s.n.* (K) |
| **Ruschioideae: Tribe Apatesieae** |  |  |
| *Apatesia helianthoides* N.E.Br. | South Africa, Western Cape | *M.G. Lavis sub BOL 23023* (BOL) |
| *Carpanthea pomeridiana* N.E.Br. | South Africa, Western Cape | *C. Klak 2471* (BOL) |
| *Conicosia elongata* (Haw.) N.E.Br. | South Africa, Northern Cape | *BOL sub STE13847* (BOL) |
| *Hymenogyne conica* L.Bolus | South Africa, Western Cape | *C. Klak 2044* (BOL) |
| *Hymenogyne glabra* (Aiton) Haw. [ssp. *namaquensis* Klak] | South Africa, Northern Cape | *C. Klak 2035* (BOL) |
| *Skiatophytum skiatophytoides* (Leistner) Klak | South Africa, Western Cape | *H. Herre sub STE 28514* (BOL) |
| *Skiatophytum tripolium* L.Bolus | South Africa, Western Cape | *O. Leistner sub STE 28402* (BOL) |
| **Ruschioideae: Tribe Dorotheantheae** |  |  |
| *Cleretum bellidiforme* (Burm.f.) G.D.Rowley | South Africa, Western Cape | Without collector, *sub STE 27801* (BOL) |
| *Cleretum booysenii* (L.Bolus) Klak | South Africa, Northern Cape | *H-D. Ihlenfeldt & M. Struck 13705* (BOL) |
| *Cleretum bruynsii* Klak | South Africa, Northern Cape | *C. Klak 2026* (BOL) |
| *Cleretum herrei* (Schwantes) Ihlenf. & Struck | South Africa, Western Cape | *P. Emms 256* (BOL) |
| *Cleretum lyratifolium* Ihlenf. & Struck | South Africa, Northern Cape | *C. Klak 2022*(BOL) |
| *Cleretum paterson-jonesii* Klak | South Africa, Northern Cape | *C. Klak 2021* (BOL) |
| *Cleretum pinnatifidum* N.E.Br. | South Africa, Western Cape | *J.F. Drèges.n.* (P04600919) |
| **Ruschioideae: Tribe Drosanthemeae** |  |  |
| *Drosanthemum asperulum* Schwantes | South Africa, Western Cape | *P.V. Bruyns 7157* (BOL) |
| *Drosanthemum bicolor* L.Bolus | South Africa, Western Cape | *P.V. Bruyns 9042* (BOL) |
| *Drosanthemum dejagerae* L.Bolus | South Africa, Northern Cape | *C. Klak 2667* (BOL) |
| *Drosanthemum lavisii* L.Bolus | South Africa, Western Cape | *P.V. Bruyns 7639* (BOL) |
| **Ruschioideae: Tribe Ruschieae** |  |  |
| *Acrodon parvifolius* R. du Plessis | South Africa, Western Cape | *C. Klak 2457* (BOL) |
| *Antimima hantamensis* (Engl.) H.E.K.Hartmann & Stüber | South Africa, Western Cape | *C. Klak 947* (BOL) |
| *Antimima solida* (L.Bolus) H.E.K.Hartmann | South Africa, Western Cape | *C. Klak 2619* (BOL) |
| *Antimima ventricosa* (L.Bolus) H.E.K.Hartmann | South Africa, Western Cape | *L.Bolus sub NBG 1946/32* (BOL) |
| *Argyroderma delaetii* C.A.Maass | South Africa, Western Cape | Without collector, *sub STE27638* (BOL) |
| *Astridia longifolia* (L.Bolus) L.Bolus | South Africa, Northern Cape | *P.V. Bruyns 7302* (BOL) |
| *Braunsia geminata* (Haw.) L.Bolus | South Africa, Western Cape | Without collector, *sub STE27840* (BOL) |
| *Carpobrotus acinaciformis* (L.) L.Bolus | South Africa, Western Cape | *C. Klak 317a* (BOL) |
| *Carpobrotus edulis* (L.) N.E.Br. | Israel, Tel-Aviv | *A. Sukhorukov* (living material) |
| *Cheiridopsis peculiaris* N.E.Br. | South Africa, Northern Cape | *C. Klak 2349* (BOL) |
| *Circandra serrata* (L.) N.E.Br. | South Africa, Western Cape | *C. Klak 1528* (BOL) |
| *Conophytum maughanii* N.E.Br. | South Africa, Northern Cape | *P.V. Bruyns 8913* (BOL) |
| *Cylindrophyllum hallii* L.Bolus | South Africa, Northern Cape | *P.V. Bruyns 7874* (BOL) |
| *Deilanthe thudichumii* (L.Bolus) S.A.Hammer | South Africa, Northern Cape | *C. Klak 2238* (BOL) |
| *Delosperma sp.* | South Africa, Western Cape | *C. Klak 2709* (BOL) |
| *Delosperma bosseranum* Marais | Madagascar, Ambovombe | *M. Decary 3532* (P04600048) |
| *Disphyma crassifolium* (L.) L.Bolus | South Africa, Western Cape | *C. Klak 1576* (BOL) |
| *Dracophilus delaetianum* (Dinter) Dinter & Schwantes | Namibia | *M. Otzen s.n.* (BOL) |
| *Eberlanzia gravida* (L.Bolus) H.E.K.Hartmann | South Africa, Northern Cape | IPC tour *274b* (BOL) |
| *Eberlanzia sedoides* (Dinter & A.Berger) Schwantes | South Africa, Northern Cape | *C. Klak 769* (BOL) |
| *Ebracteola wilmaniae* (L.Bolus) Glen | South Africa, North-West | *R. Broom sub NBG 2631/33* (BOL) |
| *Erepsia anceps* (Haw.) Schwantes | South Africa, Western Cape | *C. Klak 2608* (BOL) |
| *Erepsia dunensis* (Sond.) Klak | South Africa, Western Cape | *C. Klak 453* (BOL) |
| *Esterhuysenia drepanophylla* (Schltr. & A.Berger) H.E.K.Hartmann | South Africa, Western Cape | *E. Esterhuysen 32854* (BOL) |
| *Gibbaeum album* N.E.Br. | South Africa, Western Cape | *C. Klak 1102* (BOL) |
| *Glottiphyllum linguiforme* N.E.Br. | South Africa, Western Cape | Without collector, *sub STE 27899* (BOL) |
| *Hallianthus planus* (L.Bolus) H.E.K.Hartmann | South Africa, Western Cape | Without collector, *sub STE 28023* (BOL) |
| *Khadia acutipetala* (N.E.Br.) N.E.Br. | South Africa, Gauteng | Without collector, *sub STE 27977* (BOL) |
| *Lampranthus bicolor* (L.) N.E.Br. | South Africa, Western Cape | *E. Werdermann & E. Oberdieck 312* (B) |
| *Lampranthus explanatus* (L.Bolus) N.E.Br. | South Africa, Western Cape | *C. Klak 315* (BOL) |
| *Lampranthus reptans* (Aiton) N.E.Br. | South Africa, Western Cape | *C. Klak 317* (BOL) |
| *Lampranthus watermeyeri* (L.Bolus) N.E.Br. | South Africa, Western Cape | *C. Klak 466* (BOL) |
| *Lapidaria margaretae* Dinter & Schwantes | Namibia | Without collector, *sub STE 28017* (BOL) |
| *Leipoldtia frutescens* (L.Bolus) H.E.K.Hartmann | South Africa, Northern Cape | *P. Carrick s.n.* (BOL) |
| *Leipoldtia nevillei* Klak | South Africa, Northern Cape | *C. Klak 2032* (BOL) |
| *Lithops ruschiorum* (Dinter & Schwantes) N.E.Br. | Namibia, Swakopmund | *C. Klak s.n.* (BOL) |
| *Malephora crassa* (L.Bolus) H.Jacobsen & Schwantes | South Africa, Western Cape | Without collector, *sub STE 27953* (BOL) |
| *Mestoklema arboriforme* (Burch.) N.E.Br. ex Glen | South Africa | *K. Dahlstrand 3580* (H1463926) |
| *Mitrophyllum dissitum* Schwantes | South Africa, Northern Cape | *H.-D. Ihlenfeldt & H. Hartmann 4713* (LE) |
| *Namaquanthus vanheerdei* L.Bolus | South Africa, Northern Cape | *C. Klak 493* (BOL) |
| *Namibia ponderosa* (Dinter) Dinter & Schwantes ex H.Jacobsen | Namibia | *C. Klak 613* (BOL) |
| *Oscularia deltoides* Schwantes | Israel, Jerusalem Botanical Garden | *A. Sukhorukov* (living material) |
| *Peersia vanheerdei* (L.Bolus) H.E.K.Hartmann | South Africa, Northern Cape | *C. Klak 2662* (BOL) |
| *Pleiospilos compactus* Schwantes ssp. *canus* (Haw.) H.E.K.Hartmann & Liede | South Africa, Western Cape | *E. Esterhuysen s.n.,* BOL28108 (barcode) (BOL) |
| *Ruschia caroli* (L.Bolus) Schwantes | South Africa, Western Cape | *C. Klak 2686* (BOL) |
| *Ruschia costata* L.Bolus | South Africa, Western Cape | *C. Klak 2674* (BOL) |
| *Ruschia dejagerae* L.Bolus | South Africa, Western Cape | *C. Klak 2650* (BOL) |
| *Ruschia dichroa* (Rolfe) L.Bolus | South Africa, Western Cape | *C. Klak 2346* (BOL) |
| *Ruschia grisea* (L.Bolus) Schwantes | South Africa, Northern Cape | *C. Klak 2646* (BOL) |
| *Ruschia lineolata* Schwantes | Israel, Jerusalem Botanical Garden | *A. Sukhorukov* (living material) |
| *Ruschia multiflora* Schwantes | Israel, Jerusalem Botanical Garden | *A. Sukhorukov* (living material) |
| *Ruschia rupicola* (Engler) Schwantes | Namibia, Aus | *C. Klak 2631* (BOL) |
| *Ruschia spinosa* (L.) Dehn | South Africa, Northern Cape | *H.M. Steyn 701* (WAG1408368) |
| *Ruschia tenella* (Haw.) Schwantes | South Africa, Western Cape | *C. Klak 2669* (BOL) |
| *Ruschiella lunulata* (A.Berger) Klak | South Africa, Northern Cape | *C. Klak 2691* (BOL) |
| *Scopelogena bruynsii* Klak | South Africa, Northern Cape | *C. Klak 424* (BOL) |
| *Smicrostigma viride* (Haw.) N.E.Br. | South Africa, Western Cape | *C. Klak 180* (BOL) |
| *Stayneria neilii* (L.Bolus) L.Bolus | South Africa, Western Cape | *P.V. Bruyns 11774* (BOL) |
| *Stoeberia carpii* Friedrich | Namibia | *P.V. Bruyns 8848* (BOL) |
| *Vanheerdia roodiae* (N.E.Br.) L.Bolus | South Africa, Northern Cape | Without collector, *sub STE 28451* (BOL) |
| *Vanzijlia annulata* L.Bolus | South Africa, Western Cape | *H. Herre sub STE28456* (BOL) |
|  |  |  |
| **Outgroups** |  |  |
| *Gisekia africana* (Lour.) Kuntze (Gisekiaceae) | Namibia, Otjozondjupa Region | *A. Sukhorukov 110* (MW0582866) |
| *Glinus oppositifolius* (L.) Aug.DC. (Molluginaceae) | India, Goa State | *A. Sukhorukov 31* (MW0756339) |
| *Mollugo verticillata* L. (Molluginaceae) | Kingdom of the Netherland, Curaçao | *A. Sukhorukov* 100a (MW0982221) |
| *Kewa salsoloides* (Burch.) Christenh. (Kewaceae) | Namibia, Karas Region | *A. Sukhorukov 58* (MW0583304) |
| *Limeum obovatum* Vicary (Limeaceae) | Oman | *K.S. Price 165* (E) |
| *Lophiocarpus tenuissimus* Hook.f. (Lophiocarpaceae) | South Africa, Pretoria | *I.B. Pole-Evans s.n*. (H1738443) |
