# Supplementary material for: Evolution of seed characters and of dispersal modes in Aizoaceae
Source: Front Plant Sci. 2023 Mar 22;14:1140069. doi: 10.3389/fpls.2023.1140069 (PMC10073613; doi:10.3389/fpls.2023.1140069)
Supplement: Supplementary File 1 — Material used for the carpological examination. [file DataSheet_1.zip › Data Sheet/Supplementary file 3.doc]

***Supplementary Material***

**Evolution of seed characters and of dispersal modes in Aizoaceae**

**Alexander P. Sukhorukov*, Maya V. Nilova, Yuri Mazei, Maria Kushunina, Cornelia Klak**

*** Correspondence:** Corresponding author: suchor@mail.ru

**Supplementary file 3**. List of species with voucher information and GenBank accession numbers. All vouchers are kept at BOL, if not indicated otherwise.

| Species | *Voucher* | *trnS-G* | *rpl16* | *rps16* | *trnL-F* |
| --- | --- | --- | --- | --- | --- |
| **Outgroups** |  |  |  |  |  |
| **Molluginaceae** |  |  |  |  |  |
| *Mollugo verticillata* L. |  | - | - | FJ404927 | FJ405003 |
| *Glinus oppositifolius* (L.) Aug.DC. |  | - | - | - | MK187070 |
| **Lophiocarpaceae** |  |  |  |  |  |
| *Lophiocarpus tenuissimus* Hook.f. |  | - | - | NC040951 | - |
| **Limeaceae** |  |  |  |  |  |
| *Limeum africanum* L. | *Trinder-Smith 279* | - | - | AJ532730 | AJ558034 |
| **Gisekiaceae** |  |  |  |  |  |
| *Gisekia africana* Kuntze |  | - | - | HE585062 | HE585085 |
| **Kewaceae** |  |  |  |  |  |
| *Kewa salsoloides* (Burch.) Christenh. | *Bruyns 13053* | - | KX197710 | KX197799 | KX197538 |
| **Aizoaceae** |  |  |  |  |  |
| **Sesuvioideae** |  |  |  |  |  |
| *Anisostigma schenckii* Schinz | *Mannheimer 4920* | - | KX197790 | KX197873 | KX197613 |
| *Sesuvium curassavicum* Sukhor. | *Sukhorukov 100a* (MW) | - | - | MN906325 | MN912722 |
| *S. humifusum* (Turpin) Bohley & G.Kadereit | *L Ahart 9922* | - | - | AM161196 | AM161379 |
| *S. maritimum* Britton, Sterns & Poggenb. | - | - | - | KJ848227 | KJ848275 |
| *S. portulacastrum* (L.) L. | - | - | - | MN906333 | MG209779 |
| *S. sesuvioides* (Fenzl) Verdc. | *Klak 2431* | - | - | AJ532734 | KX197539 |
| *Trianthema portulacastrum* L. | - | - | - | KJ848216 | KJ848262 |
| *T. triquetrum* Willd. | - | - | - | KJ848226 | KJ848273 |
| *Tribulocarpus dimorphanthus* (Pax) S.Moore | *Klak 2441* | - | KX197713 | AJ532745 | KX197541 |
| *Zaleya pentandra* (L.) Jeffrey | - | - | - | HE585083 | KJ848256 |
| **Mesembryanthemoideae** |  |  |  |  |  |
| *Mesembryanthemum aitonis* Jacq. | *Bruyns 9563* | - | KX197714 | AM161197 | KM272631 |
| *M. barklyi* N.E.Br. | *Klak 767* | - | - | AJ532751 | AJ558054 |
| *M. clandestinum* Haw. | *Klak 2150* | - | - | KJ815090 | KJ815087 |
| *M. cordifolium* L. | *Klak 1019* | - | - | AM161176 | AM161373 |
| *M. coriarium* Burch. ex N.E.Br. | *Klak 990* | - | - | AM161252 | AM161434 |
| *M. crystallinum* L. | *Klak 1020* | - | - | AM161200 | AM161384 |
| *M. kuntzei* Schinz | *Bruyns 8072* | - | - | AM161188 | AY993969 |
| *M. lignescens* L.Bolus | *Bruyns 9453* | - | - | AM161223 | AM161406 |
| *M. nodiflorum* L. | *Klak 982* | - | - | AM161208 | AM161391 |
| *M. nucifer* (Ihlenf. & Bittrich) Klak | *Klak 248* | - | - | AM161193 | AY993974 |
| *M. rabiei* L.Bolus | *Klak 589b* | - | - | AJ532756 | AJ438999 |
| *M. splendens* L. subsp *splendens* | *Bruyns 9561* | - | - | AM161236 | AM161418 |
| *M. tetragonum* Thunb. | *Bruyns 8866a* | - | - | AJ532759 | AJ558061 |
| **Acrosanthoideae:** |  |  |  |  |  |
| *Acrosanthes anceps* (Thunb.) Sond. | *I Jardine 2260* | - | KX197716 | KX197800 | KX197542 |
| **Aizooideae:** |  |  |  |  |  |
| *Aizoanthemum galenioides* (Fenzl. ex Sond.) Friedrich 1 | *Mannheimer 1728* | - | KX197724 | AJ532737 | AJ558041 |
| *Aizoanthemopsis hispanica*  (L.) Klak 1 | *A.Juan & M.Juan AJ104-2* | - | KX422606 | KX422613 | KX422628 |
| *Aizoon canariense* L. 1 | *Klak 1793* | - | KX197730 | KX197814 | KX197553 |
| *Aizoon glinoides* L.f. 1 | *Dold 15008* | - | KX197732 | KX197816 | KX197555 |
| *Aizoon virgatum* Welw. ex Oliver | *Mannheimer 4913* | - | KX197742 | KX197826 | KX197565 |
| *Aizoon paniculatum* L. | *Bruyns 13188* | - | - | KY115207 | KY115204 |
| *Aizoon fruticosum* L.f. | *Klak 2432* | - | - | KX197832 | KX197571 |
| *Galenia pubescens* (Eckl. & Zeyh.) Druce 1 | *Klak 2446* | - | KX197757 | KX197841 | KX197580 |
| *Galenia secunda* Fenzl ex Sond. 1 | *Klak 2447* | - | KX197760 | KX197844 | KX197583 |
| *Gunniopsis intermedia* Diels | *RJC Chinnock s.n.* | - | KX197763 | AJ532741 | AJ558045 |
| *Gunniopsis tenuifolia* Chinnock | *RJC Chinnock 9532* | - | KX197764 | AJ532742 | AJ558046 |
| *Plinthus sericeus* Pax 1 | *Glen 1447* | - | KX197767 | KX197850 | KX197589 |
| *Tetragonia spicata* L.f. 1 | *Klak 2325* | - | KX197792 | KX197875 | KX197615 |
| *Tetragonia tetragonoides* (Pallas) O.Kuntze 1 | *Buys 1586* | - | KX197794 | KX197877 | KX197617 |
| **Ruschioideae** |  |  |  |  |  |
| **Apatesieae** |  |  |  |  |  |
| *Apatesia helianthoides* (Aiton) N.E.Br. | *Klak 800* | KP070947 | KP070988 | AJ532762 | AJ558064 |
| *Conicosia elongata* (Haw.) N.E.Br. | *Klak 752* | KP070961 | - | AJ532765 | AJ558067 |
| *Carpanthea pomeridiana* (L.) N.E.Br. | *Klak 1543* | JN896388 | KP070993 | - | - |
| *Hymenogyne conica* | *Klak 2044* | KP070939 | KP070980 | - | - |
| *Hymenogye glabra* | *Klak 2035* | KP070949 | KP070990 | - | - |
| *Skiatophytum skiatophytoides* | *Klak 805* | KP070958 | KP071000 | AJ532764 | AJ558066 |
| *Skiatophytum tripolium* | *Klak 1030* | KP070963 | KP071004 | AM161268 | AM161451 |
| **Dorotheantheae** |  |  |  |  |  |
| *Cleretum bellidiforme* (Burm.f.) G.D.Rowley | *Klak 627* | JN896383 | KF132145 | AJ532769 | AJ439000 |
| *Cleretum booysenii* | *Bruyns 10880* | JM896406 | - | - | - |
| *Cleretum bruynsii* Klak | *Klak 2026* | KP070950 | KP070991 | - | - |
| *Cleretum herrei* | *Bruyns 10628* | JN896407 | - | - | - |
| *Cleretum lyratifolium* Ihlenf. & Struck | *Klak 2022* | KP070948 | KP070989 | - | - |
| *Cleretum patersonjonesii* Klak | *Klak 2012* | KP070924 | KP070965 | - | - |
| *Cleretum pinnatifidum* | *Klak 1555* | JN896393 | - | - | - |
| **Drosanthemeae** |  |  |  |  |  |
| *Drosanthemum asperulum* (Salm-Dyck) Schwantes | *Bruyns 9005* | KF133114 | KF132147 | AJ532774 | AJ558075 |
| *D. bicolor* L.Bolus | - | LR030885 | - | - | - |
| *D. lavisii* L.Bolus | - | LR030921 | - | - | - |
| **Ruschieae** |  |  |  |  |  |
| *Acrodon subulatus* (Miller) N.E.Br. | *CREW 144* | KF133124 | KF132157 | KF132438 | KF132781 |
| *Antimima hantamensis* (Engler) H.E.K.Hartmann & Stüber | *Klak 947* | KF133129 | KF132161 | KF132441 | KF132784 |
| *Antimima solida* (L.Bolus) H.E.K.Hartmann | *Klak 1637* | KF133130 | KF132162 | KF132442 | KF132785 |
| *Antimima ventricosa* (L.Bolus) H.E.K.Hartmann | *Klak 475* | JN896381 | KF132163 | AJ532820 | AJ439015 |
| *Argyroderma fissum* (Haw.) L.Bolus | *Klak 1635* | KF133131 | KF132164 | KF132443 | KF132786 |
| *Astridia longifolia* (L.Bolus) L.Bolus | *Bruyns 7302* | KF133132 | KF132165 | AJ532816 | AJ439010 |
| *Braunsia geminata* (Haw.) L.Bolus | *Klak 205* | KF133135 | KF132168 | AJ532803 | AJ439018 |
| *Carpobrotus muirii* (L.Bolus) L.Bolus | *Klak 706* | KF133137 | KF132170 | AJ532804 | AJ439021 |
| *Cheiridopsis peculiaris* N.E.Br. | *Powell 15* | KY635352 | KY635031 | KY635111 | KY635192 |
| *Conophytum maughanii* N.E.Br. | *Bruyns 8913* | KF133148 | KF132181 | KF132452 | KF132794 |
| *Cylindrophyllum comptonii* L.Bolus | *Klak 369* | KF133150 | KF132183 | KF132454 | KF132796 |
| *Deilanthe peersii* (L.Bolus) N.E.Br. | *Klak 1694* | KF133151 | KF132184 | KF132455 | KF132797 |
| *Delosperma echinatum* (Lam.) Schwantes | *Klak 344* | KF133153 | KF132186 | AJ532775 | AJ439001 |
| *Disphyma dunsdonii* L.Bolus | *Klak 808* | KF133162 | KF132194 | AJ532771 | AJ558072 |
| *Dracophilus dealbatus* (N.E.Br.) Walgate | *Mannheimer 963B* | KF133163 | KF132195 | KF132462 | KF132804 |
| *Eberlanzia dichotoma* (L.Bolus) H.E.K.Hartmann | *Klak 497* | KF133164 | KF132196 | AJ532815 | AJ439014 |
| *Eberlanzia gravida* (L.Bolus) H.E.K.Hartmann | *Klak 1885* | KF133165 | KF132197 | KF132463 | KF132805 |
| *Ebracteola fulleri* (L.Bolus) Glen | *Klak 1650* | KF133166 | - | KF132464 | KF132806 |
| *Enarganthe octonaria* (L.Bolus) N.E.Br. | *Klak 491* | JN896380 | KF132198 | AJ532805 | AJ439023 |
| *Erepsia heteropetala* (Haw.) Schwantes | *Bruyns 9063* | KF133167 | KF132199 | KF132465 | KF132807 |
| *Erepsia inclaudens* (Haw.) Schwantes | *Bruyns 6847* | KF133168 | KF132200 | KF132466 | AJ439025 |
| *Esterhuysenia mucronata* (L.Bolus) Klak | *Klak 709* | KF133169 | KF132201 | AJ532809 | AJ439047 |
| *Gibbaeum pachypodium* (Kensit) L.Bolus | *Klak 380* | KF133175 | KF132206 | AJ532784 | AJ558082 |
| *Glottiphyllum cruciatum* (Haw.) N.E.Br. | *Bruyns 8207* | KF133176 | KF132207 | KF132470 | KF132811 |
| *Hallianthus planus* (L.Bolus) H.E.K.Hartmann | *Van Jaarsveld 16258* | KF133177 | KF132208 | KF132471 | KF132812 |
| *Khadia media* Winter & Hahn | *Klak 1960* | KF133189 | KF132220 | KF132484 | KF132825 |
| *Lampranthus bicolor* (L.) N.E.Br. | *Klak 543* | JN896384 | KF132221 | AJ532807 | AJ439042 |
| *Lampranthus schlechteri* (Zahlbruckner) L.Bolus | *Klak 844* | KF133191 | KF132223 | KF132485 | KF132826 |
| *Lapidaria margaretae* (Schwantes) Dinter & Schwantes | *Klak 1806* | KF133192 | KF132224 | KF132486 | KF132827 |
| *Leipoldtia frutescens* (L.Bolus) H.E.K.Hartmann | *Klak 1847* | KF133193 | KF132225 | KF132487 | KF132828 |
| *Leipoldtia schultzei* (Schlechter & Diels) Friedrich | *Klak 1037* | KF133194 | KF132226 | KF132488 | KF132829 |
| *Lithops julii* (Dinter & Schwantes) N.E.Br. ssp. *fulleri* (N.E.Br.) Fearn | *Klak 701* | KF133195 | KF132227 | AJ532793 | AJ439007 |
| *Machairophyllum albidum* (L.) Schwantes | *Klak 182* | KF133196 | KF132228 | AJ532801 | AJ558096 |
| *Malephora lutea* (Haw) Schwantes | *Klak 664* | KF133197 | KF132229 | AJ532785 | AJ558083 |
| *Mestoklema arboriforme* (Burchell) N.E.Br. ex Glen | *Klak 1805* | KF133199 | KF132231 | KF132490 | KF132831 |
| *Mitrophyllum clivorum* Schwantes | *Klak 1987* | KF133202 | KF132234 | - | KF132834 |
| *Namaquanthus vanheerdei* L.Bolus | *Klak 251* | KF133205 | - | AJ532810 | AJ439049 |
| *Namibia cinerea* (Marloth) Dinter & Schwantes | *Klak 615* | KF133206 | KF132237 | AJ532796 | AJ558091 |
| *Oscularia deltoides* (L.) Schwantes | *Klak 215* | KF133216 | KF132246 | AJ532786 | AJ439004 |
| *Peersia vanheerdei* (L.Bolus) H.E.K.Hartmann | *Klak 1693* | KF133218 | KF132248 | KF132500 | KF132841 |
| *Pleiospilos simulans* (Marloth) N.E.Br. | *Bruyns 4988* | KF133220 | KF132250 | AJ532824 | AJ558102 |
| *Ruschia cradockensis* (O.Kuntze) H.E.K.Hartmann | *Klak 1920* | KF133228 | KF132255 | KF132507 | KF132848 |
| *Ruschia barnardii* L.Bolus | *Klak 1808* | KF133229 | KF132256 | KF132508 | KF132849 |
| *Ruschiella lunulata* (A.Berger) Klak | *Klak 1751* | KF133241 | KF132267 | KF132519 | KF132858 |
| *Scopelogena bruynsii* Klak | *Klak 462* | KF133244 | KF132269 | AJ532811 | AJ439050 |
| *Smicrostigma viride* (Haw.) N.E.Br. | *Klak 180* | JN896379 | KF132270 | AJ532812 | AJ439051 |
| *Stayneria neilii* (L.Bolus) L.Bolus | *Bruyns 11774* | KF133245 | KF132271 | KF132522 | KF132861 |
| *Stoeberia arborea* Van Jaarsveld | *Bruyns 8363* | KF133246 | KF132272 | KF132523 | KF132862 |
| *Vanheerdia primosii* (L.Bolus) L.Bolus ex H.E.K.Hartmann | *Klak 1682* | KF133252 | KF132277 | KF132527 | KF132866 |
| *Vanzijlia annulata* (A.Berger) L.Bolus | *Klak 1822* | KF133253 | KF132278 | KF132528 | KF132867 |
